# Exome and Genome Sequencing Reveals Novel Variants for Severe Diabetic Retinopathy in Type 1 Diabetes

**DOI:** 10.1167/iovs.66.13.36

**Published:** 2025-10-23

**Authors:** Nadja Vuori, Anni A. Antikainen, Jani K. Haukka, Valma Harjutsalo, Per-Henrik Groop, Niina Sandholm, on behalf of the FinnDiane Study Group

**Affiliations:** 1 Folkhälsan Research Center, Helsinki, Finland; 2 Department of Nephrology, University of Helsinki and Helsinki University Hospital, Helsinki, Finland; 3 Research Program for Clinical and Molecular Metabolism, Faculty of Medicine, University of Helsinki, Helsinki, Finland; 4 Department of Diabetes, Central Clinical School, Monash University, Melbourne, Victoria, Australia; 5 Baker Heart and Diabetes Institute, Melbourne, Victoria, Australia

**Keywords:** diabetic retinopathy, type 1 diabetes, genetics, exome- and genome sequencing, rare variants

## Abstract

**Purpose:**

Diabetic retinopathy affects a substantial proportion of individuals with diabetes and, if not treated, may lead to acquired visual impairment or even blindness. An improved comprehension of the genetics of diabetic retinopathy (DR) is crucial in understanding the disease mechanisms. We aimed to identify rare and low-frequency variants predisposing to severe DR (SDR) in type 1 diabetes.

**Methods:**

Whole exome sequencing (WES) and whole genome sequencing (WGS) were performed for SDR in 1071 individuals with type 1 diabetes from the FinnDiane study (WES *n* = 490, WGS *n* = 581), altogether 800 with and 271 without SDR. We analyzed the genome using single variant, gene aggregate, sliding window, and regulatory regions analyses. Replication was sought in the FinnDiane genome-wide genotyping data and the UK Biobank summary statistics for WES.

**Results:**

The strongest association in the WGS data was found for an intergenic variant rs9940767 near the *IRF8* gene (*P* = 5.7 × 10^−7^), while the meta-analysis of WES and WGS found a 3′ untranslated region variant rs1239218274 on *ZNF367* (*P* = 2.31 × 10^−6^). Gene aggregate analysis results were enriched for genes expressed in the retina and identified seven genes with suggestive association with SDR (*P* < 1 × 10^−4^), with evidence of replication for *AFAP1L1*, *SLC30A9*, *HPS3*, and *PELI1*. Sliding window analyses revealed a significant association between SDR and the *CSMD2* gene (*P* = 6.84 × 10^−8^). In aggregate analyses for regulatory regions, the strongest association was found for the *PCBP4* gene promoter (*P* = 1.22 × 10^−5^).

**Conclusions:**

This study suggests rare and low-frequency variants and genes associated with SDR in type 1 diabetes, in particular *AFAP1L1*, *SLC30A9*, *HPS3*, *PELI1*, and *CSMD2*. However, further validation is required to confirm their roles and mechanisms.

Diabetic retinopathy (DR) is one of the most common complications of diabetes. The global point prevalence of DR among individuals with diabetes is estimated around 27%.^1^^,^^2^ However, more than 90% of individuals with type 1 diabetes (T1D) develop some form of DR over their lifetime, and approximately 50% to 60% of those with type 2 diabetes are also affected.^2^^–^^4^ The most severe stage of DR, that is, proliferative DR (PDR), threatens the eyesight, but can be halted by laser treatment.^5^ The pathogenesis of PDR is not completely understood, but major risk factors include long duration of diabetes and poor glycemic control, as measured by hemoglobin A1c (HbA1c); high blood pressure also increases the risk.^6^^,^^7^

Furthermore, PDR appears to accumulate in certain families, and our previous study suggested that inherited factors contribute to the onset of PDR (heritability *h^2^* = 0.52 ± 0.31).^8^ However, only a few predisposing hereditary factors have been identified for PDR. Genome-wide association studies (GWASs) on DR or severe DR (SDR)—defined as a proxy for PDR including also history of laser treatment—have suggested certain variants^9^^–^^11^ with most robust evidence of replication for variants near the *GRB2* gene.^12^ A recent study revealed a significant association of a genome-wide polygenic risk score with DR in type 2 diabetes, reinforcing the genetic background of DR.^13^ Of note, most GWASs have been performed in individuals with type 2 diabetes, with only a few studies including individuals with T1D.^10^^,^^12^^,^^14^

The number of identified rare or low-frequency variants associated with complex diseases has increased with the advances in sequencing technology. Kim et al. (2022)^15^ demonstrated that the heritability of DR is higher for the more severe forms of retinopathy and stated that rare variants account for much of the heritability of DR. Previous small whole exome sequencing (WES) studies (*n* = 15–107) found potential genes involved in DR in individuals with T1D or type 2 diabetes.^16^^,^^17^^,^^18^^,^^19^

In this study, we performed whole exome sequencing (WES) and whole genome sequencing (WGS) in a total of 1071 individuals with T1D to identify rare and low-frequency variants, either individually or aggregated within genes and genomic regions, predisposing to SDR.

## Methods

### Study Design

This study is part of the ongoing nationwide Finnish Diabetic Nephropathy (FinnDiane) Study with the aim to identify risk factors for diabetes complications. The study setting has been described previously (Supplementary Methods).^20^ The study protocol was approved by the Ethics Committee of Helsinki and Uusimaa Health District (491/E5/2006, 238/13/03/00/2015, and HUS-3313-2018), and the participants gave their written informed consent before participation. The study was conducted in accordance with the Declaration of Helsinki as revised in 2000.

Low-frequency and rare variants were analyzed in 1071 individuals with T1D from the FinnDiane study using WES and WGS to identify single variants and genes predisposing to SDR. To improve statistical power also in the noncoding region, we performed regional variant aggregate tests, including sliding window analyses, and aggregate analyses of the regulatory regions (promoters and enhancers) (Fig.). These findings were further assessed for replication in the FinnDiane GWAS and UK Biobank WES summary statistics data.^21^

**Figure.**
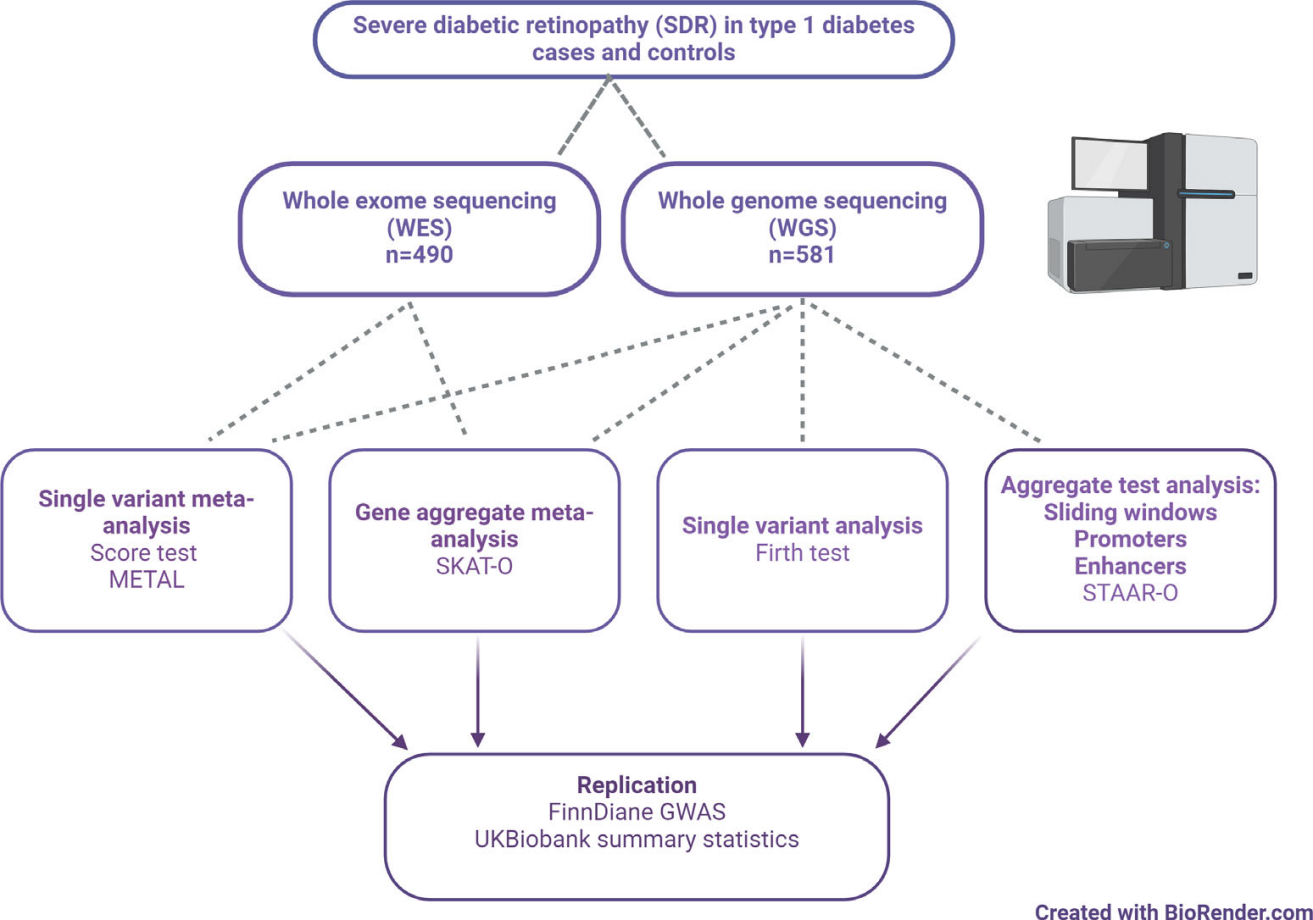
Study design.

### Study Participants

The study included 490 nonrelated individuals with WES data and 581 with WGS data (Table 1). First-degree relatives were excluded from the WES and WGS datasets. SDR was defined as the first record of PDR, laser treatment (retinal photocoagulation), or a vitrectomy event. Data on SDR were collected from multiple sources, including ophthalmologist's records and/or scored fundus photographs (available for 38% of participants), with PDR defined as an Early Treatment Diabetic Retinopathy Study score of 60 or greater; medical records and study questionnaires regarding the first year of laser treatment; and the Finnish Care Register for Health Care using the *International Classification of Diseases* codes for PDR, and Nordic Classification of Surgical Procedure codes for laser treatment or combined vitrectomy and retinal procedure by the end of 2017 (Supplementary Table S1; Supplementary Methods). Among SDR cases, 33% also had ophthalmologist's records indicating PDR (Early Treatment Diabetic Retinopathy Study score of ≥60). Participants with other retinal diseases (Supplementary Table S2) were not excluded from the analysis.

**Table 1.** Clinical Characteristics of Participants in the WES and WGS Sequencing Data

	WES			WGS		
	Cases	Controls	*P* Value	Cases	Controls	*P* Value
No	393	97		407	174	
Sex (male/female)	200/190	24/76	6.3 × 10^−6^	248/159	73/103	9.4 × 10^−6^
Age at T1D onset (years)	12.36 [8.56–17.60]	12.33 [8.25–16.71]	0.42	10.84 [6.41–15.79]	13.92 [9.07–18.79]	0.001
Calendar year of diabetes onset	1970 [1964–1976]	1964 [1959–1967]	1.2 × 10^−12^	1968 [1963–1974]	1969 [1965–1972]	0.706
Age at baseline (years)	43.49 [34.73–51.41]	51.24 [46.50–55.87]	1.5 × 10^−11^	44.4 [37.4–51.7]	48.1 [42.0–55.3]	8.0 × 10⁻⁶
Duration of T1D at baseline	29.40 [22.90–35.90]	37.60 [33.64–41.56]	<2.2 × 10^−16^	31.70 [26.40–37.00]	30.80 [27.48–34.12]	0.866
Age at follow-up/event (years)	31.81 [25.05–42.63]	65.84 [61.59–71.56]	2.2 × 10^−16^	34.29 [26.82–43.91]	62.31 [57.30–67.17]	2.2 × 10^−16^
Duration of T1D at follow-up/event (years)	18.00 [14.00–25.00]	52.50 [49.50–57.50]	2.2 × 10^−16^	22.00 [16.90–30.64]	48.50 [43.97–50.51]	2.2 × 10^−16^
Weighted mean HbA1c (%)	8.80 [8.00–10.00]	8.35 [7.80–8.78]	1.2 × 10^−5^	9.10 [8.00–10.51]	7.93 [7.45–8.45]	2.2 × 10^−16^
DKD	253 (64)	4 (4)		270 (66)	20 (11)	

DKD, participants who developed diabetic kidney disease (severe albuminuria or end-stage renal disease) during follow-up.^22^

Values are given as median [interquartile range] or number (%).

Controls were required to have at least a 20-year T1D duration without any records of SDR. Participants were originally chosen for the sequencing studies based on their diabetic kidney disease status (cases vs controls, 50% each).^22^

### Sequencing Material

WES was performed for 498 individuals using the Illumina HiSeq2000 platform, with an average requirement of 20× target capture with 80% coverage.^22^^,^^23^ WGS was performed for 599 individuals with the Illumina HiSeq X platform (Macrogen, Rockville, MD, USA) with a requirement of more than 30× average coverage for mapped reads (Supplementary Methods).^22^ The WES and WGS data were processed following the Genome Analysis Toolkit 4 Golden Standard pipeline (GATK4) as described elsewhere.^22^ The variants with a greater than 98% call rate and Hardy–Weinberg equilibrium *P* value of less than 10 × 10^−10^ were excluded, and all variants were annotated with SnpEff v5.0e.

### Statistical Methods

#### Single Variant Analyses

The single variant associations were tested with rvtests (v.2.1.0) using an additive model adjusted for sex, age at diabetes onset, and the two first genomic principal components, or, alternatively, with an additional adjustment for weighted mean HbA1c. Variants that were found only in the WGS data were analyzed with Firth regression (minor allele count [MAC] ≥5) and variants available in WES and WGS were analyzed using score test, followed by meta-analysis using fixed-effect inverse variance based meta-analysis (total MAC ≥ 5 and MAC ≥ 2 both in WES and WGS) implemented in the metal software v2011-03-25.^24^^,^^25^ Variants with *P* value of less than 5 × 10^−8^ were considered genome-wide significant, *P* values of less than 1 × 10^−7^ exome-wide significant, and *P* values of less than 1 × 10^−5^ suggestive.

#### Gene Aggregate Analyses

We performed gene aggregate analyses with a sequence kernel association omnibus test^26^ meta-analysis across WES and WGS using MetaSKAT version 0.81 package, adjusting for the same covariates as for the single variant analysis. Protein-altering variants (PAVs) and protein-truncating variants (PTVs) were analyzed separately using two minor allele frequency (MAF) thresholds of 5% (“low frequency”) and 1% (“rare”) (Supplementary Methods). Bonferroni corrected significance threshold in the PAV analyses was a *P* value of less than 3 × 10^−6^ for both rare and low frequency variants (MAF ≤1%: 15,052 genes; MAF ≤5%: 15,570 genes), and a *P* value of less than 3 × 10^−5^ for rare PTVs (1662 genes) and a *P* value of less than 1 × 10^−5^ for low-frequency PTVs (3754 genes). Gene aggregate results with a *P* value of less than 1 × 10^−4^ were considered suggestive.

#### Enrichment of Inherited Retinal Diseases

We studied whether known “inherited retinal disease-causing genes” (*N* = 458, 24 gene sets) listed by RetNet (the Retinal Information Network) and “genes with elevated expression in the retina compared to other tissues” (*N* = 785) listed by The Human Protein Atlas were significantly enriched in our gene-based meta-analysis for SDR using binomial test for proportion of genes with a *P* value of less than 0.05 in the gene sets vs all genes.

#### Sliding Window Analyses and Regulatory Regions

We performed regional variant aggregate tests on the noncoding region with the functional annotation weighted sliding window analyses using WGS data and the STAAR R package 0.9.6.^27^ Variants were weighted with MAF, combined annotation-dependent depletion (v1.6 GRCh38)^28^ score, and the first annotation principal components from multiple annotation classes as described elsewhere.^29^ Aggregate analysis of low frequency (MAF < 5%) variants was performed using an omnibus test^27^ for overlapping 4000-bp windows with a shift of 2000 bp between the window start sites across the genome (*N*_variant_ ≥ 2, cumulative MAC > 5) and covering 1,322,789 regions. Regions with a *P* value of less than 2.6 × 10^−7^ were considered significant.

To study the known gene regulatory regions, we performed a STAAR analysis to detect low-frequency variants (MAF < 5%) within transcribed enhancer and promoter regions (*N*_variant_ ≥ 2) based on FANTOM5 CAGE sequencing data on any cell or tissue type to capture the maximum of potential regulatory regions, with promoters defined as the transcription start site extended to 1000 bp. Altogether 184,515 promoters and 22,864 enhancers resulted in multiple testing correction thresholds of 2.7 × 10^−7^ and 2.2 × 10^−6^ for promoters and enhancers, respectively. Both analyses were adjusted for sex, age at diabetes onset, and the two first genomic principal components.

### Replication

The lead variants were tested for replication in the FinnDiane GWAS data consisting of 6449 individuals (Supplementary Methods).^30^^,^^31^ SDR was defined similarly as for sequencing participants, and controls were required to have at least a 30-year T1D duration without SDR. The WES and WGS participants were excluded, resulting in 2827 SDR cases and 2507 controls with T1D (Supplementary Table S1). Association was tested with rvtests using score test and genotype dosages, adjusted for age at diabetes onset, sex, genotyping batch, and kinship matrix, and additionally for weighted mean HbA1c.

Furthermore, replication for the lead variants and gene aggregate discoveries was sought from the UK Biobank summary statistics for WES on 1748 DR cases vs 386,182 non-DR controls of European ancestry (with and without diabetes).^21^

#### In Silico Variant and Gene Annotations

To identify putative target genes, we used expression quantitative trait loci (eQTL) from a retinal transcriptomic database, Eye Genotype Expression (EyeGEx; downloaded from the GTEXportal.org in 8.7.2019),^32^ and from the eQTLGen Consortium (https://eqtlgen.org/cis-eqtls.html).^33^ The genomic context of lead variants was assessed using annotations from the UCSC Genome Browser (http://genome.ucsc.edu) (hg38),^34^ including RepeatMasker Segmental Duplications, and Mappability tracks. Lead gene expression was queried in single cell RNA sequencing (scRNA-seq) for the eye from the Human Protein Atlas (https://www.proteinatlas.org/).

#### Pathway Enrichment Analysis

Pathway enrichment of lead genes from the gene aggregate tests and promoter analysis, as well as the closest genes from the enhancer analyses and sliding window regions, were tested using PAN-GO v1.0 PANTHER Overrepresentation Test^35^^–^^37^ and g:Profiler (https://biit.cs.ut.ee/gprofiler/gost).^38^ Lead genes from all analyses were jointly evaluated with STRING protein-protein interaction network analysis (https://string-db.org/).^39^

## Results

### Study Cohort

Altogether, 75% of participants had SDR (Table 1). Owing to the stringent participant selection criteria, in the WES cohort, controls had significantly longer baseline T1D duration than SDR cases (37.6 vs. 29.4 years; *P* < 2.2 × 10^−16^). No significant difference was observed in the WGS cohort (30.8 vs. 31.7 years; *P* = 0.87) (Table 1).

### Single Variant Analyses

We had 80% statistical power in the combined WES-WGS data set to detect a suggestive association (*P* < 1 × 10^−5^) with a low-frequency risk variant with MAF = 5% and an odds ratio of 5.2, or a protective variant with an odds ratio of 0.34 (Supplementary Methods and Supplementary Fig. S1).

The single variant association test for WGS data identified 11 variants within 8 independent loci suggestively associated with SDR (*P* < 1 × 10^−5^), with the strongest association for an intergenic variant rs9940767 near *IRF8* gene (MAF = 22%, *P* = 5.72 × 10^−7^) (Table 2). This variant had the lowest *P* value in the analysis adjusted for the weighted mean HbA1c (*P* = 2.53 × 10^−7^) (Supplementary Table S3). The WES–WGS meta-analysis resulted in an association with a rare deletion variant rs1239218274 on the 3′ untranslated region of *ZNF367* (Zinc Finger Protein 367) (MAF = 0.5%; *P* = 2.31 × 10^−6^) (Table 2, Supplementary Fig. S2).

**Table 2.** Suggestive Single Variant Association Results for SDR (*P* < 1 × 10^−5^), Showing Only the Lead Variant for Each Locus

Variant	Position	Data/Direction	REF/ALT	ALT AF	Potential Consequence	Gene	beta	SE	*P* Value	GWAS *P* Value	UKBB DR WES^21^ *P* Value	RegulomeDB
rs9940767	16:86117778	WGS	C/T	0.78	Intergenic variant	*IRF8*	0.77	0.15	5.72 × 10^−7^	0.25	NA	2b
rs138502562	2:21928283	WGS	G/GA	0.54	Intron variant/downstream gene variant	*ENSG00000233005 (TDRD15*, *APOB)*	−0.68	0.14	1.40 × 10^−6^	0.54	0.77	4
rs71873280	2:21928284	WGS	C/CT	0.54	Intron variant/downstream gene variant	ENSG00000233005 (*TDRD15*, *APOB*)	−0.68	0.14	1.40 × 10^−6^	0.54	NA	2b
rs59623545	7:155883231	WGS	T/C	0.18	Intergenic variant	*SHH*,*RBM33*	−0.79	0.17	2.10 × 10^−6^	0.40	NA	4
rs1239218274	9:96386550	WGS+ WES/ –^*^	CATTACAAAAAAATTCTTAGACCATTACAAATATAAATTCTCTT/C	0.005	3′ UTR variant	*ZNF367*	−3.47	0.73	2.31 × 10^−6^	0.41	NA	4
rs72660173	1:33551522	WGS	A/G	0.019	Intron variant	*CSMD2*	−2.60	0.57	5.78 × 10^−6^	0.72	NA	5
rs1186847209	8:102217827	WGS	T/TA	0.0001	Intron variant	*RRM2B*	0.79	0.18	7.22 × 10^−6^	NA	NA	3a
rs11117448	16:86115575	WGS	A/G	0.75	Intergenic variant	*IRF8*	0.65	0.15	7.66 × 10^−6^	0.96	NA	5
rs7498190	16:86115812	WGS	T/C	0.75	Intergenic variant	*IRF8*	0.65	0.15	7.66 × 10^−6^	0.92	NA	5
rs9739164	12:73395400	WGS	T/C	0.59	NA	*TRHDE*	0.59	0.13	8.00 × 10^−6^	0.19	NA	7
rs1427435362	1:123347043	WGS	G/C	NA	NA	*SRGAP2C*	−1.30	0.29	8.80 × 10^−6^	NA	NA	7
rs1511181	2:79690075	WGS	G/A	0.55	Intron variant	*CTNNA2*	−0.58	0.13	9.51 × 10^−6^	0.60	NA	7

ALT, alternative allele; ALT AF, alternative allele frequency; Gene, Underlying or closest (protein coding) gene/genes; NA, not applicable; REF, reference allele; SE, standard error; UKBB DR WES, UK Biobank Whole Exome Sequencing results for DR based on Backman et al.^21^; UTR, untranslated region.

Chromosomal position given as chromosome:base pairs using the GRCh38 genome build.

RegulomeDB scores range from 1a (strongest evidence for regulatory function) to 7 (minimal evidence). See Supplementary Table S3 for full scoring criteria.

* No heterogeneity (heterogeneity *I^2^* = 0.0, χ^2^ = 0.087, *P* = 0.768).

We attempted replication of the suggestive single variant SDR associations in the GWAS data for nonoverlapping FinnDiane individuals with T1D and in the UK Biobank WES summary statistics for DR.^21^ However, only 1 of the variants was detected in the UK Biobank, 10 in the FinnDiane study, and none of the variants replicated in these datasets.

### Gene Aggregate Analyses

Gene aggregate tests for PAVs and PTVs were performed to increase the statistical power to identify low-frequency and rare variants. Altogether, seven genes were suggestively associated with SDR (*P* < 1 × 10^−4^), the strongest association being with the *AFAP1L1* gene, including nine rare PAVs with an MAF of less than 1% (*P* = 1.56 × 10^−5^) (Table 3). Of the nine PAVs, rs766943760 was the most strongly associated with SDR (*P* = 1.65 × 10^−5^) (Supplementary Table S4) and was predicted deleterious or possibly damaging by the SIFT and PolyPhen algorithms.

**Table 3.** Suggestive Gene Aggregate Analysis Results for SDR (*P* < 1 × 10^−4^)

GENE	MAF Threshold	N Variant	Variant Type	*P* Value	*P* Value, Adjusted for HbA1c	FinnDiane GWAS Replication	UKBB DR WES
*AFAP1L1*	0.01	9	PAV	1.56 × 10^−5^	7.12 × 10^−5^	NA	MAF ≤ 0.001%: *P* = 0.03
*ST6GAL1*	0.05	4	PAV	1.58 × 10^−5^	1.07 × 10^−5^	NA	NA
*SLC30A9*	0.05	7	PAV	3.62 × 10^−5^	0.0002	rs149368642 (*P* = 0.04)	Singleton: *P* = 0.04
*HPS3*	0.05	9	PAV	4.93 × 10^−5^	5.44 × 10^−5^	NA	MAF ≤ 1%, ≤ 0.1%: *P* = 0.02
*PELI1*	0.01 and 0.05	3	PAV	7.21 × 10^−5^	0.001	NA	MAF ≤ 0.001%: *P* = 0.049
*UACA*	0.01 and 0.05	2	PTV	7.63 × 10^−5^	0.008	NA	NA
*USP15*	0.01 and 0.05	4	PAV	7.92 × 10^−5^	0.002	NA	NA

NA, not applicable.

UKBB DR WES, UK Biobank Whole Exome Sequencing results for DR based on Backman et al.^21^

Gene burden replication in the UK Biobank study with putative LoF variants (pLOF) (M1) and pLOF + deleterious missense variants (M3). Genes with PAVs were replicated with M3 and genes with PTVs with M1 in the UK Biobank study.

The *UACA* gene with two PTVs had a close to significant burden of SDR-associated PTVs (*P* = 7.63 × 10^−5^). Three additional genes not detected in the main analysis were suggestively associated when additionally adjusted for weighted mean HbA1c (Supplementary Table S5 and S6).

Replication of the seven gene-aggregate test lead genes was tested in the UK Biobank WES gene-aggregate data.^21^ Evidence of replication was found for *AFAP1L1* with a missense variant model (MAF ≤ 0.001%: *P* = 0.032), as well as for *SLC30A9*, *HPS3*, and *PELI1* (Table 3). *UACA* did not replicate for DR, but interestingly, was associated with hypertension in the burden analyses in the UK Biobank (MAF ≤ 0.1%: *P* = 0.029).^40^

Only a few of the PAVs in the associated genes were found in the FinnDiane GWAS data, mostly owing to their rareness; however, one missense variant (rs149368642) in *SLC30A9* replicated in the FinnDiane GWAS (*P* = 0.036) and was predicted deleterious (low confidence) or probably damaging by SIFT and PolyPhen algorithms (Supplementary Table S4).

### Sliding Window and Regulatory Region Analyses

To increase statistical power to identify noncoding genetic variants, we performed sliding window aggregate analyses weighted by functional annotations. We discovered a genetic region within the *CSMD2* gene with significant burden of low-frequency SDR-associated variants (*P* = 6.84 × 10^−8^) (Table 4, Supplementary Fig. S3). An intronic variant in this gene was also among the strongest single variant associations (*P* = 5.78 × 10^−6^) (Supplementary Table S7).

**Table 4.** Sliding Window-Based Test Results

Chromosome:Position	N Variant	MAC	*P* Value	Gene
1:33550001–33554000	15	111	6.84 × 10^−8^	*CSMD2* ^*^
1:33548001–33552000	11	110	9.48 × 10^−8^	*CSMD2* ^*^
7:148668001–148672000	17	95	3.18 × 10^−6^	*CUL1*
9:118980001–118984000	20	136	4.37 × 10^−6^	*BRINP1*
9:118718001–118722000	28	146	6.53 × 10^−6^	*BRINP1*
18:2794001–2798000	18	137	7.12 × 10^−6^	*SMCHD1* ^*^
20:55134001–55138000	29	129	7.22 × 10^−6^	*DOK5*
1:9236001–9240000	21	93	7.38 × 10^−6^	*H6PD* ^*^
8:6232001–6236000	27	169	7.87 × 10^−6^	*MCPH1*
4:131746001–131750000	166	2258	8.53 × 10^−6^	*PCDH10*
9:119000001–119004000	31	245	8.59 × 10^−6^	*BRINP1*
18:880001–884000	16	74	9.00 × 10^−6^	*ADCYAP1*
20:55132001–55136000	30	137	9.34 × 10^−6^	*DOK5*

N variant, number of variants in the region.

Gene is the closest protein coding gene.

Significant (*P* < 2.6 × 10^−7^) and suggestively significant sliding-window regions (*P* < 1 × 10^−^^5^).

* Inside the gene.

To further focus on the noncoding regions with known regulatory function, we performed aggregate analysis of low-frequency variants (MAF < 5%) on FANTOM5 enhancer and promoter regions. Variants in two enhancer regions, within *RETREG1* (Reticulophagy Regulator 1) and near *CBX4* (Chromobox 4), showed suggestive enrichment for SDR-associated variants (*P* < 1 × 10^−4^) (Table 5, Supplementary Fig. S4, Supplementary Table S8).

**Table 5.** Regulatory Region (Enhancers and Promoters) Aggregate Analysis Results for Variants With MAF <5% and With Regional Enrichment *P* Value < 1 × 10^−4^

Chromosome:Position	N Variant	CMAC	*P* Value	Gene
Enhancer regions				
5:16540456–16540738	2	34	2.97 × 10^−5^	*RETREG1* ^*^
17:79841509–79841692	2	43	6.20 × 10^−5^	*CBX4*
Promoter regions				
3:51958535–51959535	5	13	1.22 × 10^−5^	*PCBP4*
4:39961204–39962204	4	5	1.42 × 10^−5^	*PDS5A*
5:16539644–16540644	6	74	2.17 × 10^−5^	*RETREG1*
15:38130526–38131526	6	81	3.17 × 10^−5^	*NA*
1:3020470–3021470	6	75	3.97 × 10^−5^	*ACTRT2* ^†^
10:26566128–26567128	6	41	4.39 × 10^−5^	*APBB1IP*
7:116239248–116240248	2	37	4.45 × 10^−5^	*TES*
14:52313816–52314816	8	31	4.88 × 10^−5^	*PTGER2*
1:11847209–11848209	8	86	5.12 × 10^−5^	*NPPA* ^†^
5:14212115–14213115	3	13	6.45 × 10^−5^	*TRIO*
16:85899010–85900010	2	8	6.79 × 10^−5^	*IRF8*
21:21507855–21508855	6	30	8.07 × 10^−5^	*NCAM2*
15:77046423–77047423	5	28	8.36 × 10^−5^	*TSPAN3*

CMAC, cumulative MAC across all included variants within the region; N variants, number of variants in the region; NA, not applicable.

Gene is the closest protein coding gene/the gene closest to the enhancer, or the corresponding gene for the gene promoter regions.

*P* value: Bonferroni-corrected significance threshold of *P* < 2 × 10^−6^ for enhancers and *P* < 3 × 10^−7^ for promoters.

* Inside the gene.

† One locus reported.

The strongest signal in the promoter analysis was on the *PCBP4* (Poly(RC) Binding Protein 4) promoter region (*P* = 1.22 × 10^−5^) (Table 5, Supplementary Fig. S5). *PCBP4* is highly expressed in the retina (Supplementary Fig. S7). Promoters for altogether 13 genes reached a suggestive *P* value of less than 1 × 10^−4^, including the *RETREG1* promoter (*P* = 2.17 × 10^−5^) partly overlapping with the *RETREG1* enhancer region, and multiple partly overlapping promoter regions for *NPPA* (*P* = 5.12 × 10^−5^) gene transcripts (Supplementary Fig. S5); two variants on *NPPA* promoter regions replicated in the FinnDiane GWAS (*P* < 0.05). Lead variant rs198370 in the *NPPA* gene was associated with *NPPA-AS1* expression (*P* = 1.46 × 10^−14^) in the retinal eQTL data as well as with *NPPA-AS1*, *NPPA*, *MTHFR*, and *CLCN6* in the eQTL data for whole blood (Supplementary Table S9). Another *NPPA* promoter lead variant rs5064 demonstrated functional evidence in RegulomeDB, and was a *trans*-eQTL for retinal *ACBD5* expression on chromosome 10 (*P* = 1.93 × 10^−14^); of note, we observed also retinal *cis*-eQTL association (*P* = 0.05) between *ACBD5* and an *APBB1IP* promoter region variant rs10829025 associated with SDR. *ACBD5* encodes acyl-coenzyme A binding domain containing protein 5, expressed in retinal cells, whereby mutations in the gene cause *ACBD5*-related retinal dystrophy (*ACBD5*, OMIM 616618).^41^ Therefore, our findings suggest that less severe regulatory variants affecting retinal *ACBD5* expression are linked to SDR.

To assess the potential influence of sequencing artifacts, we evaluated the genomic context of lead variants and regions. Four single variants were located on repeat-rich or noise-prone regions on chromosomes 16p11.2 and 8p23.1, which are known for segmental duplications, copy number variation, and lower mapping quality (Supplementary Table S10).

### CACNB2 Validation

The *CACNB2* gene encodes a subunit of a voltage-dependent L-type calcium channel, and we previously showed that the gene regulates VEGF expression and secretion from retinal pigment epithelial cells.^42^ In the current WGS data, the strongest association within ±500 kb from the previous lead variant rs11014284 was observed for an intronic variant rs7099509 in *CACNB2* (*P* = 0.0015). Within other subunits of the L-type calcium channel, PAVs in three genes (*CACNA1C* [*P* = 0.001, MAF < 1%], *CACNG7* [*P* = 0.007, MAF < 1% and MAF < 5%], and *CACNB3* [*P* = 0.04, MAF < 5%]) were associated with SDR in gene aggregate analyses.

### Gene Set Enrichment Analysis

We additionally examined whether retinal disease genes were overrepresented among the SDR-associated genes in our data (Supplementary Table S11). The “genes with elevated expression in the retina compared to other tissues” were significantly enriched for association with SDR, as 37 out of 785 genes were associated (*P* < 0.05) with SDR in our gene aggregate meta-analysis (binomial test *P* = 0.0033).

### Pathway Enrichment Analysis

PAN-GO pathway enrichment analysis of lead promoter regions indicated overrepresentation of biological processes related to response to alcohol and ketone, and prostaglandin E response (false discovery rate < 0.05) (Supplementary Table S12). Furthermore, g:Profiler analysis of the lead genes from the promoter analysis indicated enrichment of KEGG Renin secretion pathway (false discovery rate = 0.0469) (Supplementary Table S13). This pathway is involved in vascular regulation and may contribute to the pathogenesis of SDR through mechanisms related to blood pressure and endothelial function. Finally, STRING network analysis identified protein-protein interactions between *ADCYAP1* (sliding window), *PTGER2*, and *NPPA* (promoters) (Supplementary Fig. S6), all involved in vascular signaling and neuroendocrine regulation.

## Discussion

Although only a few genetic variants have been thus far associated with DR, previous studies suggest that rare variants account for much of the heritability of DR^15^ and that genetics play a more important role in the development of severe forms of DR.^15^^,^^43^ Therefore, the aim of this study was to identify rare and low frequency variants associated with SDR in 1071 individuals with T1D, consisting of 490 individuals with WES data and 581 individuals with WGS data. By performing single variant, gene aggregate, and regulatory region aggregate tests, we identified several putative associations with SDR.

In the single variant analysis, the strongest evidence for association (*P* = 5.72 × 10^−7^) was found for a common intergenic variant near the *IRF8* gene*. IRF8* (Interferon Regulatory Factor 8) is a transcription factor of the IRF family. Despite the lack of replication for this finding, the promoter region on *IRF8* was also identified in the regulatory regions analysis. Notably, *IRF8* has been implicated in the development of pathological neovascularization in the eye^44^ and also plays a role in immune regulation,^45^ which is closely linked to DR pathogenesis.

Gene aggregate analyses detected multiple genes with burden of PAVs or PTVs suggestively associated with SDR. Evidence of replication was found for *AFAP1L1*, *SLC30A9*, *HPS3*, and *PELI1*. In addition, *AFAP1L1* and *HPS3* remained suggestively associated with SDR when adjusted for HbA1c. All these genes are expressed in the retina (Supplementary Fig. S7). Furthermore, one out of seven PAVs in *SLC30A9* replicated for SDR in T1D (FinnDiane GWAS) and was predicted deleterious and probably damaging by the SIFT and PolyPhen algorithms. *SLC30A9* (Solute Carrier Family 30 Member 9) encodes a zinc transporter linked to Birk–Landau–Perez syndrome, a rare disorder with ocular and renal symptoms.^46^^,^^47^ *SLC30A9* may influence retinal oxidative stress, because zinc imbalance is associated with retinal degeneration and diabetic complications. *SLC30A9* shows moderate to high expression across several retinal cell types (Supplementary Fig. S7). Also, in the *AFAP1L1* (Actin Filament Associated Protein 1 Like 1) gene, the strongest variant out of the nine PAVs in the gene (rs766943760) was predicted deleterious or possibly damaging. *AFAP1L1* promotes cell proliferation and prevents apoptosis in lung cancer cells, and its important paralog, *AFAP1*, is associated with glaucoma.^48^ *AFAP1L1* is expressed in retina, and especially on Muller glial cells in the eye scRNA-seq data (Supplementary Fig. S7). Interestingly, hypoxia-induced *AFAP1L1* regulates pathological retinal neovascularization in mice and is presented as a promising therapeutic target to slow the progression of neovascular eye disease.^49^

*HPS3* (HPS3 Biogenesis of Lysosomal Organelles Complex 2 Subunit 1) is associated with the Hermansky–Pudlak syndrome 3 (HPS3) causing visual impairment among other symptoms.^50^ *PELI1* (Pellino E3 Ubiquitin Protein Ligase 1) is an inflammation-related gene expressed in retinal tissue, involved in immune signaling and regulation of RPE cell survival.^51^ Its link to microvascular injury in diabetes suggests a role in the inflammatory processes underlying DR.^52^

The sliding window aggregate analysis, also covering the noncoding regions of the genome, discovered a significant genomic window in the *CSMD2* gene (CUB And Sushi Multiple Domains 2) (*P* = 6.84 × 10^−8^). This gene is involved in the regulation of the complement cascade of the immune system,^53^ which also plays an important role in SDR. The *CSMD2* encoded protein is localized in the synaptic layers of the retina which suggests a role in neurovascular interactions.^54^

Among the promoter analysis lead findings, two variants in the *NPPA* gene replicated in the FinnDiane GWAS. *NPPA* encodes atrial natriuretic peptide/factor (ANP), a cardiac signaling molecule that acts as an antigrowth factor in endothelial cells, counteracting the angiogenic and permeability actions of VEGF.^55^ ANP has been shown to inhibit VEGF-induced vascular leakage and angiogenesis in vivo.^56^ High levels of ANP in membranes and vitreous humor in individuals with PDR have been measured by semiquantitative RT-PCR and RIA,^57^ supporting a role for ANP in PDR.

Finally, pathway enrichment analysis of the promoter regions suggested involvement of prostaglandin E2 in SDR. Of note, studies in rodents suggest that prostaglandin E2 and its receptor signaling pathway promotes DR.^58^ Although nonsteroidal anti-inflammatory drugs reduce the production of prostaglandins and other prostanoids through inhibition of the upstream arachidonic acid metabolism, their effect on DR remains inconclusive, and more specific prostaglandin receptor inhibitors have been suggested for the treatment of DR and other retinopathies.^59^

Our study is unique both regarding the T1D population and the use of both WES and WGS data focusing on rare and low-frequency variants associated with SDR. Recently, DR was analyzed in a UK Biobank WES study of 454,787 participants across 3994 traits.^21^ However, the approach of the registry-based definition of DR, no specific data on SDR, and inclusion of nondiabetic controls may miss variants effective only in a hyperglycemic/diabetic environment and could falsely point toward diabetes-associated variants.

Although our discovery study participants have well-defined T1D, one limitation of our study is the partial reliance on registry-based treatment and diagnostic codes for case and control classification, which may lack uniform ophthalmic confirmation. Given the heterogeneity of these data sources and limited clinical confirmation of PDR, SDR was used as a proxy for advanced retinopathy, enabling consistent classification without assuming uniform diagnostic certainty. Nonetheless, we believe our multisource approach provides a robust and clinically meaningful SDR phenotype for genetic association analysis.

Owing to the initial extreme sample selection strategy,^23^ the WES controls had significantly longer T1D duration, and consequently, an earlier T1D onset year on average. Given the improvements in diabetes treatment and the decreasing incidence of PDR,^60^ this makes the controls even more extreme survivors. However, there may also be generational differences between the SDR cases and controls, and the genetic predisposition to SDR may interact with the changing epigenetic and environmental influences that could modulate the disease progression. In addition, rising diabetes prevalence and potential shifts in SDR incidence over time may affect the generalizability of our findings and highlight the need for longitudinal, population-specific studies.

Although, to date, our study is the largest next-generation sequencing study on SDR in T1D, we still had limited power to detect low-frequency variants with moderate effect sizes. To overcome the limited power, we have used aggregate tests both within the gene coding regions, as well as in the noncoding regions with an emphasis on the functionally relevant regions such as known gene promoter and enhancer regions. Nevertheless, each method has limitations that should be acknowledged. The single-variant analysis is well-powered to detect associations with common variants but lacks sensitivity for rare variants unless the effect sizes are large. The gene aggregate tests, although more powerful for detecting the cumulative effects of rare variants within genes, are sensitive to the choice of aggregation strategy and variant filtering criteria. They may also miss associations driven by a single strong variant or by regulatory variants outside the coding regions. The regulatory region and sliding window analyses broaden the search to noncoding regions, but are more exploratory and prone to false positives owing to the large number of tests and less well-characterized functional annotations. Therefore, findings from each method should be interpreted in the context of these limitations, and larger studies are still needed to robustly identify both common and rare genetic factors underlying SDR.

Our replication strategy included GWAS data in individuals with T1D (FinnDiane GWAS) and sequencing data in the general population (UK Biobank WES).^21^ Neither dataset provides optimal replication: the UK Biobank lacks SDR-specific phenotypes, includes nondiabetic individuals, and is predominantly composed of type 2 diabetes cases, whereas the rarity of many variants in the FinnDiane GWAS limits statistical power. However, to our knowledge no other large-scale sequencing-based data exist for individuals with T1D and with detailed retinal phenotyping. Future efforts should prioritize replication in T1D-specific cohorts to validate these findings and support clinical translation.

A key limitation of our study is the lack of experimental validation to confirm the pathogenicity of the identified rare variants. Although our approach was designed as a discovery-phase genetic association study, we acknowledge that statistical associations alone are insufficient to establish causality, particularly for rare variants with predicted or unknown functions. Furthermore, our analyses were limited to germline variation and did not incorporate epigenomic datasets such as ATAC-seq. Although we used eye scRNA-seq data to annotate lead genes and retinal eQTL data to assess the regulatory impact of identified common variants—providing functional support for several loci—future studies integrating multi-omics data such as retinal ATAC-seq and other epigenomic datasets will be essential to elucidate the molecular mechanisms underlying the findings. Finally, functional studies will be needed to validate top candidate genes in relevant cellular models and, where feasible, in vivo using established DR models. These efforts are essential to move from association to mechanism and assessing therapeutic relevance.

To summarize, identifying genetic variants that contribute to the risk of SDR is crucial for gaining deeper insights into the molecular mechanisms underlying this severe diabetic complication and for early identification of individuals at high risk of vision loss. This study revealed rare and low-frequency SDR-associated variants using WES and WGS in individuals with T1D, highlighting in particular the role of *AFAP1L1*, *SLC30A9*, *HPS3*, *PELI1*, and *CSMD2*; further studies are needed to confirm their role and the underlying molecular mechanisms in SDR.

## Supplementary Material

Supplement 1
