# Supplementary material for: Exome and Genome Sequencing Reveals Novel Variants for Severe Diabetic Retinopathy in Type 1 Diabetes
Source: Invest Ophthalmol Vis Sci. 2025 Oct 23;66(13):36. doi: 10.1167/iovs.66.13.36 (PMC12553471; doi:10.1167/iovs.66.13.36)
Supplement: Supplement 1 [file iovs-66-13-36_s001.docx]

***Exome and genome sequencing reveals novel variants for severe diabetic retinopathy in type 1 diabetes***

Nadja Vuori, Anni Antikainen, Jani Haukka, Valma Harjutsalo, Per-Henrik Groop, Niina Sandholm, on behalf of the FinnDiane Study Group

**Supplementary material**

[**Supplementary Methods** 2](#_Toc211846191)

[**Supplementary Table S1. The inclusion and exclusion criteria for each cohort used in the study** 5](#_Toc211846192)

[**Supplementary Table S2:** **Other retinal disease diagnoses among SDR cases and controls** 6](#_Toc211846193)

[**Supplementary Table S3. HbA1c adjusted single variant association results for SDR (P < 1×10^-5^)** 7](#_Toc211846194)

[**Supplementary Table S4. Single variant association results for each gene aggregate test lead gene** 9](#_Toc211846195)

[**Supplementary Table S5. HbA1c adjusted gene aggregate analysis results for SDR (P < 1×10^-4^)** 11](#_Toc211846196)

[**Supplementary Table S6. HbA1c adjusted single variant results for each gene aggregate test lead gene** 12](#_Toc211846197)

[**Supplementary Table S7. Variants in the discovered genomic windows (STAAR-O) with replication in type 1 diabetes (FinnDiane), RegulomeDB probability rankings, and target genes from Retina eQTL and eQTLgen** 13](#_Toc211846198)

[**Supplementary Table S8. Variants in the discovered enhancers (STAAR-O) with replication in type 1 diabetes (FinnDiane) and available Retina eQTL data and eQTLgen** 25](#_Toc211846199)

[**Supplementary Table S9. Variants in the discovered promoters (STAAR-O) with replication in type 1 diabetes (FinnDiane) and available Retina eQTL data and eQTLgen** 26](#_Toc211846200)

[**Supplementary Table S10: Evaluation of the lead loci and regions for overlap with repeat-rich or noise-prone regions** 30](#_Toc211846201)

[**Supplementary Table S11. Enrichment analysis of A. Inherited retinal disease-causing genes, Voltage-dependent Calcium channel genes, and B. Genes with elevated expression in the retina compared to other tissues in the gene aggregate SKAT-O meta-analysis for SDR. SDR-associated genes at MAF < 0.05 and MAF < 0.01** 31](#_Toc211846202)

[**Supplementary Table S12. Significantly enriched pathways (false discovery rate, FDR < 0.05) from the PAN-GO PANTHER Overrepresentation Test for the 12 promoter analysis lead genes** 34](#_Toc211846203)

[**Supplementary Table S13. g:Profiler Enrichment Summary** 35](#_Toc211846204)

[**Supplementary Table S14: FinnDiane Study Centers** 36](#_Toc211846205)

[**Supplementary Figure S1. Statistical power to detect suggestive associations (P < 1 ×10^-5^) with SDR** 39](#_Toc211846206)

[**Supplementary Figure S2. Manhattan plot of single variant results** 40](#_Toc211846207)

[**Supplementary Figure S3. Manhattan plot for sliding window-based tests (STAAR)** 40](#_Toc211846208)

[**Supplementary Figure S4. Manhattan plot for enhancer regions with low-frequency variants (MAF < 5%) (STAAR)** 41](#_Toc211846209)

[**Supplementary Figure S5. Manhattan plot for promoter regions with low-frequency variants (MAF < 5%) (STAAR)** 41](#_Toc211846210)

[**Supplementary Figure S6. STRING network analysis** 42](#_Toc211846211)

[**Supplementary Figure S7. Single-cell tissue expression for genes associated with SDR: *IRF8* (A), *ZNF367* (B), *AFAP1L1* (C), *SLC30A9* (D), *HPS3* (E), *PELI1* (F), *CSMD2* (G), and *PCBP4* (H)** 43](#_Toc211846212)

[**References** 49](#_Toc211846213)

**Supplementary Methods**

**Study participants**

Study participants are part of the Finnish Diabetic Nephropathy Study (FinnDiane), an ongoing nationwide prospective study established in 1997 to identify risk factors for long-term diabetic complications. The study included 490 non-related individuals with WES data and 581 with WGS data. SDR was defined as the first record of proliferative diabetic retinopathy, laser treatment (retinal photocoagulation), or a vitrectomy event. Data on Severe diabetic retinopathy (SDR) were collected from the medical records and study questionnaires regarding the first year of laser treatment, and from the ophthalmologist’s records for proliferative diabetic retinopathy defined as ETDRS score 60 or higher, or from the Finnish Care Register for Health Care using the ICD codes for proliferative retinopathy (ICD-9: 3620B; ICD-10: H3603) and Nordic Classification of Surgical Procedure codes for laser treatment or combined vitrectomy and retinal procedure (CKC10, CKC12, CKC15, CKC50, CKD40, CKD92, CKD93, 3724, 3721) by the end of 2017. The registry data were available for all, while the other types of data were available only for a subset of participants. Controls were required to have at least a 20-year type 1 diabetes duration without any records of SDR. Participants were originally chosen for the sequencing studies based on their diabetic kidney disease status (cases vs controls, 50% each).^1^

**Sequencing material**

WES was performed for 498 individuals using the Illumina HiSeq2000 platform, with an average requirement of 20× target capture with 80% coverage, as described earlier.^1,2^ WGS was performed with the Illumina HiSeq X platform (Macrogen Inc., Rockville, MD, USA) with a requirement of >30× average coverage for mapped reads.^1^ The WES and WGS data were processed following the Genome Analysis Toolkit 4 Golden Standard pipeline (GATK4) as described earlier. In short, Fastq reads were trimmed with Trimmomatic, sample quality was reassessed with FastQC, and the results were aggregated with MultiQC. The reads were aligned by lanes with Burrows-Wheeler Aligner (BWA), sorted, and duplicates were removed with Picard’s SortSam and MarkDuplicates tools, and then recalibrated by chromosome with GATK’s VQSR and ApplyVQSR tools. Variants were called with the HaplotypeCaller tool’s ERC mode into a single sample GVCF-file with GATK’s CombineGVCFs and GenotypeGVCFs tools and filtered with an excess heterozygosity threshold of 54.69, truth sensitivity level of 99.7%, and GATK’s recommended tranche thresholds for SNPs and insertion/ deletion variants (indels). The variants with <98% call rate and HWE p-value <10×10-10 were excluded, and all variants were annotated with SnpEff v5.0e.

**Statistical material**

**Single variant analyses**

The single variant associations were tested using rvtests (v.2.1.0) with an additive model adjusted for sex, age at diabetes onset, and the first two genomic principal components, or alternatively, with an additional adjustment for weighted mean HbA1c. Variants that were found only in the WGS data were analyzed with Firth regression, and variants with minor allele count (MAC) <5 were excluded from the analysis. Variants that were also found in the WES data were analyzed using a score test, followed by meta-analysis of the WES and WGS results conducted using fixed-effect inverse variance-based meta-analysis implemented in the METAL software v2011-03-25.^3,4^ The variants were required to have MAC≥2 both in WES and WGS, and total MAC≥5. Variants with P-value <5×10^-8^ were considered genome-wide significant, P <1×10^−7^ exome-wide significant, and P <1×10^-5^ suggestive.

*Power calculation*: Power to detect single-variant association with α=1×10^-5^ significance level was calculated with the R genpwr package v.1.0.4, assuming an additive model and WES and WGS samples pooled together.

**Gene aggregate analysis**

To study the association between SDR and the protein-coding variants within each gene, we performed gene aggregate analysis with a sequence kernel association omnibus test (SKAT-O).^5^ SKAT-O meta-analysis of WES and WGS was conducted using the MetaSKAT version 0.81 package, adjusting for the same covariates as for the single variant analysis. Protein-altering variants (PAV) and protein-truncating variants (PTV) were analyzed separately using two minor allele frequency (MAF) thresholds, 5% (“low frequency”) and 1% (“rare”).

Variants with a MAC≥1 were included in the analysis, but the genes were required to entail at least two variants and a cumulative MAC ≥5. Multiple testing corrected threshold for statistical significance in the PAV analyses was a p-value <3×10^-6^ for both rare and low frequency variants (MAF≤1%:15,052 genes; MAF≤5%:15,570 genes), and P <3×10^-5^ for rare PTVs (1,662 genes) and p<1×10^-5^ for low frequency PTVs (3,754 genes). Gene aggregate results with P <1×10^-4^ were considered suggestive.

*Variant type classification:* PTVs in our analysis included variants that are predicted to shorten the coded protein length, also considered as putative loss-of-function mutations, including the start lost variants, stop gained or lost variants, frameshift variants, exon loss variants, and splice acceptor and donor variants. PAVs in our analysis included variants that alter the amino acid sequence of the coded protein. In addition to classical missense mutations, these also included 5’ UTR premature start codon gain variants, 5’ UTR truncation & exon loss variants, in-frame insertions and deletions, and all PTVs.

**Sliding window analysis**

We performed regional variant aggregate tests on the non-coding region with the functional annotation weighted sliding window analyses using WGS data and the STAAR R package 0.9.6.^6^ Variants were annotated based on the Combined Annotation-Dependent Depletion (CADD) v1.6 GRCh38 data^7^, and associations within the regional windows were tested using an omnibus test.^6^ Variants were weighted with MAF, CADD score, and the first annotation principal components (aPC) from multiple annotation classes as described earlier^8^ and transformed to the PHRED scale with appropriate direction. The first principal component from each PCA, which was calculated with CADD data as described earlier^8^ was considered as the aPC score.^1,6^ Aggregate analysis of low frequency (MAF<5%) variants was performed for 4,000 bp windows separated by 2,000 bp skips across the genome, i.e., 50% overlapping with the previous window, requiring at least two variants per window (CMAC>5) and covering 1,322,789 regions. Regions with P < 2.6 × 10^−7^ were considered significant.

To study the gene regulatory regions, we performed STAAR analysis to detect low-frequency variants (MAF<5%) within transcribed enhancer and promoter regions from the FANTOM5 CAGE sequencing data on GRCh38 coordinates. We further extended the promoter regions 1000 bp downstream of the CAGE TSS to capture a wider promoter region. Variants were weighted with MAF, requiring at least two variants. Altogether 184,515 promoters and 22,864 enhancers resulted in multiple testing correction thresholds of 2,7×10^-7^ and 2.2×10^-6^ for promoters and enhancers, respectively. Both analyses were adjusted for sex, age at diabetes onset, and the first two genomic principal components. Promoter and enhancer region tissue and cell-type specificity was investigated for the lead regions from the FANTOM5 Zenbu database (https://fantom.gsc.riken.jp/5/).

**Replication**

*FinnDiane GWAS:* The samples were genotyped with Illumina HumanCoreExome Bead arrays, genotypes called with the zCall algorithm, and initial quality control performed at the University of Virginia as described earlier.^9^ Genotyping data were converted from GRCh37/hg19 to build version 38 (GRCh38/hg38) and merged from four batches. Variants with high missingness (>2%), low HWE p-value (<10^-6^), or minor allele count (MAC) <3 were removed. Individuals with high genotype missingness (>5%) and excess heterozygosity (±4 standard deviations) were excluded. Samples were pre-phased with Eagle 2.3.5, and genotype imputation was performed with Beagle 4.1 (version 08Jun17.d8b) based on the population-specific SISu v3 imputation reference panel with WGS data for 3,775 Finnish individuals as described earlier.^10^ Variants were limited to those with a high imputation quality of r^2^>0.8.

##

## **Supplementary Table S1. The inclusion and exclusion criteria for each cohort used in the study**

| **Cohort** | **Data type** | **Diabetes definition** | **SDR case definition** | **Control definition** |
| --- | --- | --- | --- | --- |
| Discovery Study |  | T1D diagnosis AND diabetes onset age <40 AND insulin initiated within one calendar year from the diabetes diagnosis | SDR defined by PDR (ETDRS≥60; or ICD-9: 3620B; ICD-10: H3603), laser treatment or vitrectomy (CKC10, CKC12, CKC15, CKC50, CKD40, CKD92, CKD93, 3724, 3721) | No registry data or other data for PDR by the end of follow-up at 31/12/2017 AND  ≥20 years T1D duration at the end of follow-up |
|  | WES (N=490) |  | N=393 | N=97 |
|  | WGS (N=581) |  | N=407 | N=147 |
| Replication 1  (single variant and gene aggregate test results) | FinnDiane GWAS (N=6449 after QC) | Same as Discovery.  N=5334  (WES and WGS participants excluded) | Same as Discovery. N=2827 | No registry data or other data for PDR by the end of follow-up at 31/12/2017 AND  ≥30 years T1D duration at the end of follow-up.  N=2507 |
| Replication 2 (single variant and gene aggregate test results) | UK Biobank WES (N=454787) | NA | Record for Diabetic Retinopathy.  N=1748 | All participants of European ancestry with no records of DR (including individuals without diabetes).  N=386182 |

## **Supplementary Table S2:** **Other retinal disease diagnoses among SDR cases and controls**

|  | **WES (N=490)** | | **WGS (N=581)** | |
| --- | --- | --- | --- | --- |
| **Retinal diseases** | **N** | **ETDRS Confirmed PDR (N, %)** | **N** | **ETDRS Confirmed PDR (N, %)** |
| **SDR cases (records of PDR, laser treatment, or vitrectomy)** | | | | |
| All SDR cases | 393 | 154 (39%) | 407 | 107 (26%) |
| SDR cases without other retinal disorders | 367 | 150 (41%) | 375 | 105 (28%) |
| SDR cases with RVO | 6 | 2 (33%) | 5 | 0 (0%) |
| SDR cases with AMD | 16 | 1 (6%) | 14 | 0 (0%) |
| SDR cases with optic neuropathy | 4 | 1 (25%) | 13 | 2 (15%) |
|  | **N** | **ETDRS scoring available (N, %)** | **N** | **ETDRS scoring available (N, %)** |
| **Non-SDR controls** | | | | |
| All non-SDR controls | 97 | 27 (28%) | 174 | 41 (24%) |
| Controls with RVO | 0 | 0 (0%) | 3 | 0 (0%) |
| Controls with AMD | 3 | 0 (0%) | 4 | 0 (0%) |
| Controls with optic neuropathy | 0 | 0 (0%) | 3 | 0 (0%) |

ETDRS confirmed PDR: retinal records or fundus photographs available confirming PDR, defined as ETDRS ≥60. RVO, retinal vein occlusion; AMD, age-related macular degeneration.

**Supplementary Table S3. HbA1c adjusted single variant association results for SDR (P < 1×10^-5^)**

Multiple variants per gene are shown.

| **Variant** | **Position** | **Data/**  **Direction** | **REF/ALT** | **ALT AF** | **Potential Consequence** | **Gene** | **beta** | **SE** | **p-value** | **Het *I^2^*** | **Het χ^2^** | **Het P-Val** | **FinnDiane GWAS p-value** | **Ukbb DR WES p-value** | **RegulomeDB** |
| --- | --- | --- | --- | --- | --- | --- | --- | --- | --- | --- | --- | --- | --- | --- | --- |
| rs9940767 | 16:86117778 | WGS | C/T | 0.78 | Intergenic variant | nearest gene *IRF8* | 0.83 | 0.16 | 2.53x10^-7^ | - | - | - | 0.25 | - | 2b |
| rs59623545 | 7:155883231 | WGS | T/C | 0.18 | Intergenic variant | *SHH** | -0.86 | 0.18 | 1.13x10^-6^ | - | - | - | 0.40 | - | 4 |
| rs1427435362 | 1:123347043 | WGS | G/C | - | - | - | -1.53 | 0.32 | 2.39x10^-6^ | - | - | - | - | - | 7 |
| rs41274480 | 1:10419734 | WGS+WES/++ | G/A | 0.047 | Synonymous Variant | *PGD* | 1.25 | 0.27 | 2.65x10^-6^ | 0.0 | 0.006 | 0.9408 | 0.31 | - | 1f |
| rs80319203 | 17:14348429 | WGS | T/C | 0.063 | Non-Coding Transcript Variant | *HS3ST3B1* | 1.62 | 0.35 | 2.99x10^-6^ | - | - | - | 0.78 | - | 1f |
| rs72867441 | 1:10380979 | WGS+WES/-- | A/G | 0.048 | 3 Prime UTR Variant | *KIF1B* | -1.21 | 0.26 | 4.96x10^-6^ | 0.0 | 0.000 | 0.9832 | 0.22 | - | 1f |
| rs17034660 | 1:10282464 | WGS+WES/++ | G/A | 0.048 | Synonymous Variant | *KIF1B* | 1.21 | 0.26 | 4.96x10^-6^ | 0.0 | 0.000 | 0.9832 | 0.22 | - | 7 |
| rs2229688 | 1:10413143 | WGS+WES/++ | G/A | 0.048 | Missense Variant | *PGD* | -1.21 | 0.26 | 4.96x10^-6^ | 0.0 | 0.000 | 0.9832 | 0.22 | 0.88 | 1f |
| rs2297881 | 1:10337509 | WGS+WES/-- | A/G | 0.048 | Missense Variant | *KIF1B* | 2.76 | 0.26 | 4.96x10^-6^ | 0.0 | 0.000 | 0.9832 | 0.22 | 0.28 | 1f |
| rs189785327 | 17:82601563 | WGS+WES/++ | C/T | 0.009 | Non-Coding Transcript Variant | *FOXK2* | 1.19 | 0.61 | 5.23x10^-6^ | 0.0 | 0.390 | 0.5321 | 0.27 | - | 3a |
| rs10015765 | 4:153195608 | WGS | A/G | 0.34 | Intron Variant | *TRIM2* | -0.63 | 0.14 | 5.63x10^-6^ | - | - | - | 0.53 | - | 7 |
| rs1368944065 | 15:101288265 | WGS | G/T | 0.035 | Intron Variant | *SNRPA1* | -1.55 | 0.34 | 6.32x10^-6^ | - | - | - | - | - | 6 |
| rs9570472 | 13:61123303 | WGS | G/T | 0.12 | - | *-* | -0.98 | 0.22 | 6.58x10^-6^ | - | - | - | 0.17 | - | 1f |
| rs62251066 | 3:56867133 | WGS | G/A | 0.11 | Intron Variant | *ARHGEF3* | -0.99 | 0.22 | 6.95x10^-6^ | - | - | - | 0.45 | - | 4 |
| rs2360964 | 1:199085011 | WGS | T/G | 0.38 | - | *-* | 0.65 | 0.15 | 7.83x10^-6^ | - | - | - | 0.98 | - | 5 |
| rs61760196 | 1:10130517 | WGS+WES/++ | G/C | 0.047 | Missense Variant | *UBE4B* | 1.19 | 0.27 | 8.08x10^-6^ | 0.0 | 0.001 | 0.9751 | 0.31 | - | 1f |
| rs13110620 | 4:113901423 | WGS+WES/-- | G/A | 0.76 | 3 Prime UTR Variant | *ARSJ* | -0.56 | 0.12 | 8.43x10^-6^ | 0.0 | 0.009 | 0.9253 | 0.66 | - | 7 |
| rs4452507 | 4:113901449 | WGS+WES/++ | A/C | 0.76 | 3 Prime UTR Variant | *ARSJ* | 0.56 | 0.12 | 8.43x10^-6^ | 0.0 | 0.009 | 0.9253 | 0.66 | - | 6 |
| rs79448557 | 17:14344445 | WGS | T/C | 0.058 | Intron Variant | *HS3ST3B1* | 1.61 | 0.36 | 8.69x10^-6^ | - | - | - | 0.99 | - | 1f |
| rs60109222 | 1:199099687 | WGS | G/GTA | 0.37 | 500B Downstream Variant | *LOC107985243* | 0.65 | 0.15 | 9.03x10^-6^ | - | - | - | - | - | 7 |
| rs1041596561 | 1:10376864 | WGS+WES/-- | CACAT/C | 0.048 | 3 Prime UTR Variant | *KIF1B* | -1.19 | 0.27 | 9.05x10^-6^ | 0.0 | 0.001 | 0.9692 | - | - | 2b |
| rs1511181 | 2:79690075 | WGS | G/A | 0.55 | Intron Variant | *CTNNA2* | -0.60 | 0.14 | 9.48x10^-6^ | - | - | - | 0.60 | - | 7 |
| rs10919623 | 1:199122116 | WGS | G/T | 0.38 | Intron Variant | *LOC107985243* | 0.65 | 0.15 | 9.55x10^-6^ | - | - | - | 0.97 | - | 7 |
| rs10800609 | 1:199122571 | WGS | T/G | 0.38 | Intron Variant | *LOC107985243* | 0.65 | 0.15 | 9.55x10^-6^ | - | - | - | 0.97 | - | 2b |
| rs762416428 | 4:84447419 | WGS | G/GTA | 0.069 | - | *-* | 1.51 | 0.34 | 9.57x10^-6^ | - | - | - | 0.29 | - | 5 |
| rs773940091 | 4:84447420 | WGS | GCA/G | 0.069 | - | *-* | 1.51 | 0.34 | 9.57x10^-6^ | - | - | - | 0.29 | - | 5 |
| rs17008935 | 4:84449373 | WGS | C/T | 0.069 | - | *-* | 1.51 | 0.34 | 9.57x10^-6^ | - | - | - | 0.28 | - | 5 |

Chromosomal position given as chromosome:base pairs using the GRCh38 genome build. Abbreviations: REF, Reference allele; ALT, Alternative allele; ALT AF, Alternative Allele Frequency; SE, Standard error; Het I^2,^ Het χ2, Het P-Val: Heterogeneity *I^2^* and *χ2* estimates and *p*-value. Gene: Underlying or closest (protein coding) gene/genes; Ukbb DR WES, UK Biobank Diabetic Retinopathy Whole Exome Sequencing Study.

+ +Meta-analysis with positive minor allele effect direction, -- Meta-analysis with negative minor allele effect direction.

RegulomeDB ranking score

Score Supporting data

1a eQTL/caQTL + TF binding + matched TF motif + matched Footprint + chromatin accessibility peak

1b eQTL/caQTL + TF binding + any motif + Footprint + chromatin accessibility peak

1c eQTL/caQTL + TF binding + matched TF motif + chromatin accessibility peak

1d eQTL/caQTL + TF binding + any motif + chromatin accessibility peak

1e eQTL/caQTL + TF binding + matched TF motif

1f eQTL/caQTL + TF binding / chromatin accessibility peak

2a TF binding + matched TF motif + matched Footprint + chromatin accessibility peak

2b TF binding + any motif + Footprint + chromatin accessibility peak

2c TF binding + matched TF motif + chromatin accessibility peak

3a TF binding + any motif + chromatin accessibility peak

3b TF binding + matched TF motif

4 TF binding + chromatin accessibility peak

5 TF binding or chromatin accessibility peak

6 Motif hit

7 Other

## **Supplementary Table S4.** **Single variant association results for each gene aggregate test lead gene**

| **GENE** | **Variant type** | **Position** | **Variant** | **REF/ALT** | **SIFT** | **Polyphen** | **Data** | **MAF*** | **WES, WGS score p-value** | **FinnDiane GWAS p-value** |
| --- | --- | --- | --- | --- | --- | --- | --- | --- | --- | --- |
| *AFAP1L1* | PAV 1% | 5:149306327  5:149316264  -”-  5:149317776  5:149302443  5:149317845  5:149322633  5:149322678  5:149322706  5:149332861 | rs200939762  rs190469835  -”-  rs768819246  rs776465096  rs753270486  rs1222650442  rs114805975  rs750418239  rs766943760 | C/T  C/A  -“-  C/T  C/G  G/A  G/T  C/T  G/T  G/T | Deleterious  Tolerated  -“-  Tolerated  Deleterious  Tolerated  Tolerated  Deleterious  Deleterious  Deleterious | Probably_damaging  Benign  -“-  Benign  Probably_damaging  Benign  Benign  Probably_damaging  Probably_damaging  Possibly_damaging | WGS  WGS  WES  WGS  WES  WES  WES  WES  WES  WES | MAC≤3  MAC≤3  MAC≤3  MAC≤3  MAC≤3  MAC≤3  MAC≤3  MAC≤3  MAC≤3  0.00408 | 0.05002  0.41820  0.51793  0.19662  0.73465  0.11174  0.51807  0.72127  0.10914  1.65×10^-5^ | -  0.445  -”-  -  -  -  -  0.595  -  - |
| *ST6GAL1* | PAV 5% | 3:186930704  3:186963801  -”-  3:187042926  3:187074272  -”- | rs765720308  rs3733000  -”-  rs151284888  rs184402390  -”- | C/T  T/C  -“-  C/T  C/T  -“- | -  -  -“-  Deleterious  -  -“- | -  -  -“-  Benign  -  -“- | WGS  WGS  WES  WGS  WGS  WES | MAC≤3  0.03959  0.02755  MAC≤3  MAC≤3  MAC≤3 | -  3.19×10^-5^  0.03109  0.61378  0.56816  0.22916 | -  0.104  -”-  -  0.498  -”- |
| *SLC30A9* | PAV 5% | 4:42001660  -”-  4:42018125  -”-  4:42022902  4:42078282  4:42038998  4:42039004 | rs149368642  -”-  rs2581423  -”-  rs780740348  -  -  - | C/T  -“-  G/A  -“-  G/A  A/T  A/G  T/A | Deleterious_lc  -“-  -  -“-  Deleterious  Deleterious  Tolerated  Tolerated | Probably_damaging  -“-  -  -“-  Possibly_damaging  Probably_damaging  Benign  Benign | WGS  WES  WGS  WES  WGS  WGS  WES  WES | MAC≤3  MAC≤3  0.02324  0.02653  MAC≤3  MAC≤3  MAC≤3  MAC≤3 | 0.00681  0.00424  0.47089  0.01428  0.17794  0.21512  0.09812  0.09812 | 0.036  -”-  0.775  -”-  -  -  -  - |
| *HPS3* | PAV 5% | 3:149129895  3:149145378  3:149153578  3:149162248  3:149162256  -”-  3:149140378  3:149141356  3:149145519  3:149157401 | -  rs778630258  rs138303522  rs749184890  rs78336249  -”-  rs144990171  rs764381762  rs149620802  rs780982970 | T/C  A/C  C/A  C/A  G/A  -“-  G/A  C/G  C/T  C/T | Deleterious  Tolerated  Tolerated  Tolerated  Deleterious  -“-  Tolerated  Deleterious  Deleterious  Deleterious | Probably_damaging  Benign  Probably_damaging  Benign  Benign  -“-  Benign  Probably_damaging  Probably_damaging  Probably_damaging | WGS  WGS  WGS  WGS  WGS  WES  WES  WES  WES  WES | MAC≤3  MAC≤3  MAC≤3  MAC≤3  0.01377  0.00816  MAC≤3  MAC≤3  MAC≤3  MAC≤3 | 0.21549  0.27066  0.01480  0.31002  0.02966  0.55458  0.12400  0.24692  0.10581  0.71987 | -  -  0.181  -  0.935  -”-  -  -  -  - |
| *PELI1* | PAV 1% & 5% | 2:64096526  2:64095198  2:64108259 | rs200658046  rs747065672  - | C/T  C/A  C/A | Tolerated_lc  Deleterious_lc  Tolerated_lc | Benign  Probably_damaging  Probably_damaging | WGS  WES  WES | MAC≤3  MAC≤3  MAC≤3 | 0.09283  0.00426  0.00397 | -  -  - |
| *UACA* | PTV 1% & 5% | 15:70667338  -”-  15:70679607 | rs781623644  -”-  rs185763236 | G/A  -”-  C/A | -  -”-  - | -  -”-  - | WGS  WES  WGS | MAC≤3  MAC≤3  MAC≤3 | 0.00571  0.01846  0.00571 | 0.261  -”-  - |
| *USP15* | PAV 1% & 5% | 12:62260437  12:62325932  12:62391426 | rs199606121  rs1275290495  - | A/C  A/G  G/T | -  Deleterious_lc  Tolerated_lc | -  Probably_damaging  Possibly_damaging | WES  WES  WES | MAC≤3  MAC≤3  MAC≤3 | 0.12780  0.00485  0.00171 | -  -  - |

The chromosomal position given as chromosome:base pairs using the GRCh38 genome build. Abbreviations: REF, Reference allele; ALT, Alternative allele; MAF, Minor allele frequency.

Pathogenicity prediction tools: SIFT and PolyPhen. * Minor allele count (MAC) is reported instead of minor allele frequency (MAF) for extremely rare variants (MAC≤3) to protect rare variant carriers’ privacy and enhance the table readability.

## **Supplementary Table S5. HbA1c adjusted gene aggregate analysis results for SDR (P < 1×10^-4^)**

| **GENE** | **P-value** | **MAF threshold** | **N variant** | **Variant type** | **FinnDiane GWAS replication** | **Ukbb DR WES**  **replication** |
| --- | --- | --- | --- | --- | --- | --- |
| *SCPEP1* | 1.02×10^-5^ | 0.01 & 0.05 | 4 | PAV | - | - |
| *ST6GAL1* | 1.07×10^-5^ | 0.05 | 4 | PAV | - | - |
| *WAC* | 1.19×10^-5^ | 0.01 & 0.05 | 6 | PAV | - | - |
| *HPS3* | 5.44×10^-5^ | 0.05 | 9 | PAV | - | MAF ≤ 1%, ≤ 0.1%:  p=0.02 |
| *AFAPIL1* | 7.12×10^-5^ | 0.01 | 9 | PAV | - | MAF ≤ 0.001%:  p=0.03 |
| *ARV1* | 8.39×10^-5^ | 0.01 & 0.05 | 5 | PAV | - | - |

Abbreviations: MAF, Minor allele frequency. Gene burden replication in the UK Biobank study with putative LoF variants (pLOF) (M1) and pLOF + deleterious missense variants (M3).

Genes with PAVs were replicated with M3, and genes with PTVs with M1.

## **Supplementary Table S6. HbA1c adjusted single variant results for each gene aggregate test lead gene**

| **GENE** | **Variant type** | **Position** | **Variant** | **REF/ALT** | **Data** | **MAF** **†** | **WES, WGS score p-value** | **FinnDiane GWAS p-value** |
| --- | --- | --- | --- | --- | --- | --- | --- | --- |
| *OBSCN-AS1* | PAV 1% & 5% | 1:228212550  1:228212564 | rs1226153147  rs2037784302 | G/C  G/A | WES  WES | MAC≤3  MAC≤3 | -  - | -  - |
| *SCPEP1* | PAV 1% & 5% | 17:56981197  -”-  17:56985381  17:56978218  17:56987702 | rs140119129  -”-  rs201362915  rs1910971011  rs376039843 | C/A  -“-  G/A  T/C  C/A | WGS  WES  WGS  WES  WES | 0.00430  MAC≤3  MAC≤3  MAC≤3  MAC≤3 | 1.1× 10^-6^  0.492  -  -  - | 0.463  -“-  -  -  - |
| *ST6GAL1** | PAV 5% |  |  |  |  |  |  |  |
| *WAC* | PAV 1% & 5% | 10:28583492  10:28589844  10:28583438  10:28583450  10:28583460  10:28583492  10:28589806 | rs559564372  rs753401903  rs1839641557  rs1365886112  rs559564372  rs766802511 | A/G  C/T  G/A  G/A  A/T  A/G  G/A | WGS  WGS  WES  WES  WES  WES  WES | MAC≤3  MAC≤3  MAC≤3  MAC≤3  MAC≤3  MAC≤3  MAC≤3 | 0.014  -  -  -  -  -  - | -  -  -  -  -  -  - |
| *HPS3** | PAV 5% |  |  |  |  |  |  |  |
| *AFAP1L1** | PAV 1% |  |  |  |  |  |  |  |
| *ARV1* | PAV 1% & 5% | 1:230979169  -”-  1:230979178  -”-  1:230990114  1:230990164  1:230979122 | rs143532693  -”-  rs140251959  -”-  rs772391110  rs201097730  rs754195455 | A/T  -“-  G/T  -“-  A/C  T/C  G/A | WGS  WES  WGS  WES  WGS  WGS  WES | 0.00602  MAC≤3  MAC≤3  MAC≤3  MAC≤3  MAC≤3  MAC≤3 | 0.001  0.450  -  -  -  -  - | 0.133  -”-  0.339  -”-  -  -  - |

*Available in the unadjusted model

Abbreviations: REF, Reference allele; ALT, Alternative allele; MAF, Minor allele frequency. † Minor allele count (MAC) is reported instead of minor allele frequency (MAF) for extremely rare variants (MAC≤3) to protect rare variant carriers’ privacy and enhance the table readability.

## **Supplementary Table S7. Variants in the discovered genomic windows (STAAR-O) with replication in type 1 diabetes (FinnDiane), RegulomeDB probability rankings, and target genes from Retina eQTL and eQTLgen**

| **Window** | **Closest gene** | **Position** | **Variant** | **REF/ALT** | **Beta** | **SE** | **p-value** | **FinnDiane GWAS p-value** | **Regulome DB** | **Retina eQTL target gene (p-value)** | **eQTLgen (p-value)** |
| --- | --- | --- | --- | --- | --- | --- | --- | --- | --- | --- | --- |
| 1:33550001-33554000 | *CSMD2* | 1:33551192 | rs1426512591 | G/C | 0.59 | 2.12 | 0.78 | 0.807 | 5 | n | n |
|  |  | 1:33551522 | rs72660173 | A/G | -2.60 | 0.57 | 5.67E-06 | 0.722 | 5 | n | n |
|  |  | 1:33551738 | rs1356724620 | T/C | 0.04 | 2.12 | 0.99 | n | 5 | n | n |
|  |  | 1:33551905 | rs188539040 | G/A | -0.45 | 1.23 | 0.71 | 0.412 | 4 | n | n |
|  |  | 1:33551925 | rs61801994 | G/A | 0.06 | 0.33 | 0.85 | 0.455 | 2b | n | n |
|  |  | 1:33552502 | rs115277139 | T/C | 0.35 | 2.12 | 0.87 | 0.597 | 5 | n | n |
|  |  | 1:33552704 | rs146064267 | C/A | -1.15 | 0.93 | 0.22 | 0.121 | 5 | n | n |
|  |  | 1:33552811 | rs561507151 | C/T | -2.63 | 1.99 | 0.19 | n | 5 | n | n |
|  |  | 1:33552872 | rs182209154 | G/A | 0.16 | 0.61 | 0.80 | 0.397 | 5 | n | n |
|  |  | 1:33553156 | rs115953178 | C/G | -0.89 | 1.48 | 0.55 | 0.941 | 6 | n | n |
|  |  | 1:33553191 | rs918121381 | G/A | 0.82 | 2.12 | 0.70 | 0.740 | 7 | n | n |
|  |  | 1:33553594 | rs534853887 | C/T | 0.77 | 2.12 | 0.715 | 0.637 | 7 | n | n |
|  |  | 1:33553768 | rs142272146 | T/C | 0.68 | 0.96 | 0.48 | 0.232 | 5 | n | n |
|  |  | 1:33553771 | - | C/A | -1.91 | 2.12 | 0.37 | n | n | n | n |
|  |  | 1:33553942 | rs1657708432 | C/T | 0.19 | 2.12 | 0.93 | n | n | n | n |
| 1:33548001-33552000 | *CSMD2* | 1:33548175 | - | CTGT/C | 0.59 | 0.73 | 0.42 | 0.807 | n | n | n |
|  |  | 1:33548284 | rs138812986 | A/G | 0.59 | 0.73 | 0.42 | 0.807 | 4 | n | n |
|  |  | 1:33548520 | rs144731188 | G/A | 0.60 | 1.01 | 0.55 | 0.743 | 2a | n | n |
|  |  | 1:33548968 | - | T/A | 1.10 | 2.12 | 0.61 | n | n | n | n |
|  |  | 1:33549175 | rs955215979 | C/T | 0.82 | 2.12 | 0.70 | 0.743 | 7 | n | n |
|  |  | 1:33549815 | - | G/C | -1.50 | 2.12 | 0.48 | n | n | n | n |
| 7:14866800-148672000 | *CUL1* | 7:148668324 | rs538400799 | T/G | -2.22 | 2.12 | 0.30 | 0.612 | 5 | n | n |
|  |  | 7:148668445 | rs1013454852 | A/G | -1.39 | 2.12 | 0.51 | n | 4 | n | n |
|  |  | 7:148668548 | rs76561970 | G/A | -1.85 | 2.12 | 0.382 | 0.79 | 7 | n | n |
|  |  | 7:148668729 | rs1797211252 | T/C | -2.02 | 2.12 | 0.341 | n | n | n | n |
|  |  | 7:148668907 | rs534171416 | G/A | -1.37 | 1.42 | 0.334 | 0.482 | 4 | n | n |
|  |  | 7:148668981 | rs562047921 | A/G | -0.65 | 0.50 | 0.196 | 0.001 | 5 | n | n |
|  |  | 7:148669744 | rs180757684 | C/T | -0.19 | 0.70 | 0.791 | 0.888 | 4 | n | n |
|  |  | 7:148669824 | rs773220558 | C/T | 0.53 | 2.12 | 0.801 | 0.588 | 5 | n | n |
|  |  | 7:148669963 | rs189305961 | C/T | -0.62 | 1.44 | 0.667 | 0.310 | 4 | n | n |
|  |  | 7:148670121 | rs113036123 | A/C | -1.26 | 0.61 | 0.040 | 0.099 | 4 | n | n |
|  |  | 7:148670714 | rs142569585 | A/G | -2.37 | 1.90 | 0.21 | 0.458 | 4 | n | n |
|  |  | 7:148670848 | rs556663423 | T/C | -2.81 | 2.13 | 0.19 | 0.572 | 4 | n | n |
|  |  | 7:148670980 | rs149836279 | G/A | -0.47 | 0.45 | 0.29 | 0.876 | 2b | n | n |
|  |  | 7:148671013 | rs117292046 | A/G | -0.97 | 0.49 | 0.049 | 0.074 | 4 | n | RP4-800G7.2 (0.0000027) |
|  |  | 7:148671607 | rs1300182850 | A/C | -2.72 | 2.12 | 0.20 | n | 4 | n | n |
|  |  | 7:148671651 | rs146551265 | G/C | 0.40 | 2.12 | 0.85 | n | 4 | n | n |
|  |  | 7:148671754 | rs1037373137 | A/G | -1.97 | 1.22 | 0.10 | n | 4 | n | n |
| 9:118980001-118984000 | *BRINP1* | 9:118980008 | rs1048651490 | T/G | -0.10 | 1.42 | 0.95 | n | 6 | n | n |
|  |  | 9:118980009 | rs865892704 | T/G | -0.07 | 0.37 | 0.85 | n | 6 | n | n |
|  |  | 9:118980422 | rs574273763 | G/A | 0.47 | 2.12 | 0.83 | n | 7 | n | n |
|  |  | 9:118980599 | rs74467275 | A/G | -2.93 | 0.80 | 0.00027 | 0.602 | 7 | n | n |
|  |  | 9:118981433 | rs909682268 | G/A | -2.64 | 2.12 | 0.21 | n | 5 | n | n |
|  |  | 9:118981640 | rs556946472 | A/G | -1.87 | 2.12 | 0.38 | n | 7 | n | n |
|  |  | 9:118982054 | rs2807706 | C/T | 2.93 | 0.80 | 0.00027 | 0.602 | 5 | n | n |
|  |  | 9:118982181 | - | T/C | 0.46 | 2.12 | 0.83 | n | n | n | n |
|  |  | 9:118982637 | rs1363612432 | G/A | 0.12 | 2.13 | 0.95 | n | 7 | n | n |
|  |  | 9:118983409 | rs62572698 | A/G | 0.25 | 0.63 | 0.69 | 0.746 | 7 | n | n |
|  |  | 9:118983477 | rs768850816 | T/A | -1.61 | 2.12 | 0.45 | n | 5 | n | n |
|  |  | 9:118983538 | rs1829818821 | A/G | -0.16 | 2.12 | 0.94 | n | n | n | n |
|  |  | 9:118983539 | 0 | AAC/A | -0.90 | 1.43 | 0.53 | n | n | n | n |
|  |  | 9:118983553 | rs62572699 | C/T | 0.74 | 0.94 | 0.43 | n | 5 | n | n |
|  |  | 9:118983558 | rs1407586788 | G/GCA | 0.61 | 1.91 | 0.75 | n | 5 | n | n |
|  |  | 9:118983564 | rs62572700 | A/G | -0.02 | 0.61 | 0.98 | n | 5 | n | n |
|  |  | 9:118983566 | rs62572701 | A/G | -2.78 | 2.12 | 0.19 | n | 5 | n | n |
|  |  | 9:118983673 | rs2807709 | A/C | 2.93 | 0.80 | 0.00027 | 0.602 | 6 | n | n |
|  |  | 9:118983767 | rs78296364 | C/T | -3.50 | 1.61 | 0.030 | 0.914 | 7 | n | n |
|  |  | 9:118983931 | rs576371534 | C/T | -2.87 | 2.12 | 0.18 | 0.888 | 5 | n | n |
| 9:118718001-118722000 | *BRINP1* | 9:118718624 | rs10984191 | G/T | -2.74 | 0.94 | 0.0037 | 0.825 | 5 | n | n |
|  |  | 9:118718844 | - | A/G | 0.64 | 2.12 | 0.76 | n | n | n | n |
|  |  | 9:118718919 | rs1213849316 | A/C | 0.63 | 1.96 | 0.75 | 0.107 | 5 | n | n |
|  |  | 9:118718946 | rs12235868 | T/C | -2.69 | 0.97 | 0.0056 | 0.825 | 5 | n | n |
|  |  | 9:118718968 | rs1485773983 | A/G | -0.59 | 2.13 | 0.78 | 0.041 | 5 | n | n |
|  |  | 9:118719020 | rs12235164 | C/T | -2.69 | 0.97 | 0.0056 | 0.825 | 5 | n | n |
|  |  | 9:118719031 | rs1056927563 | T/C | -2.60 | 1.94 | 0.18 | n | 5 | n | n |
|  |  | 9:118719112 | rs12235170 | C/A | -2.69 | 0.97 | 0.0056 | 0.825 | 7 | n | n |
|  |  | 9:118719165 | rs1346814299 | A/G | -2.07 | 2.12 | 0.33 | n | 7 | n | n |
|  |  | 9:118719167 | rs12235180 | G/A | -2.69 | 0.97 | 0.0056 | 0.825 | 7 | n | n |
|  |  | 9:118719259 | - | AG/A | 0.65 | 2.12 | 0.76 | n | n | n | n |
|  |  | 9:118719479 | rs190335436 | A/C | -2.07 | 2.12 | 0.33 | 0.936 | 7 | n | n |
|  |  | 9:118719503 | rs10984192 | C/G | -2.69 | 0.97 | 0.0056 | 0.825 | 7 | n | n |
|  |  | 9:118719645 | rs765394678 | C/G | -0.65 | 1.43 | 0.65 | 0.201 | 7 | n | n |
|  |  | 9:118719873 | rs138640528 | A/G | -2.74 | 0.86 | 0.0015 | 0.328 | 7 | n | n |
|  |  | 9:118720178 | rs189848293 | C/T | 0.87 | 0.94 | 0.35 | 0.246 | 7 | n | n |
|  |  | 9:118720521 | rs537804966 | T/C | -0.41 | 0.87 | 0.64 | 0.282 | 7 | n | n |
|  |  | 9:118720847 | rs916280226 | T/C | -0.48 | 0.78 | 0.54 | 0.277 | 6 | n | n |
|  |  | 9:118720891 | rs776034238 | G/A | -2.83 | 1.94 | 0.14 | n | 7 | n | n |
|  |  | 9:118720942 | rs548382464 | C/G | 0.00 | 2.12 | 1.00 | 0.224 | 6 | n | n |
|  |  | 9:118721017 | - | CA/C | 0.88 | 2.12 | 0.68 | n | n | n | n |
|  |  | 9:118721022 | rs187463922 | A/T | -0.72 | 0.54 | 0.18 | n | 6 | n | n |
|  |  | 9:118721030 | rs1972071 | A/T | -2.74 | 0.94 | 0.0037 | n | 6 | n | n |
|  |  | 9:118721034 | rs1013677825 | T/A | -1.59 | 2.12 | 0.45 | n | 6 | n | n |
|  |  | 9:118721331 | rs7038555 | T/A | -2.74 | 0.94 | 0.0037 | 0.825 | 5 | n | n |
|  |  | 9:118721453 | rs546511928 | C/T | -0.06 | 0.52 | 0.91 | 0.293 | 4 | n | n |
|  |  | 9:118721897 | - | T/A | 0.64 | 2.12 | 0.76 | n | n | n | n |
|  |  | 9:118721942 | rs773563055 | C/G | 1.31 | 2.13 | 0.54 | 0.794 | 2b | n | n |
|  |  | 9:118721996 | rs142954240 | C/T | 1.23 | 1.76 | 0.48 | 0.909 | 4 | n | n |
| 18:2794001-2798000 | *SMCHD1* | 18:2794055 | rs141269187 | G/T | -1.40 | 1.02 | 0.17 | 0.418 | 5 | n | n |
|  |  | 18:2794260 | - | G/A | -0.07 | 2.12 | 0.97 | n | n | n | n |
|  |  | 18:2794357 | - | TTTGCCTGTAATCCGAGCTA/T | 1.70 | 2.07 | 0.41 | 0.186 | n | n | n |
|  |  | 18:2794401 | rs764769704 | G/A | 1.19 | 2.12 | 0.58 | n | 4 | n | n |
|  |  | 18:2794541 | rs186865940 | T/C | -1.69 | 2.12 | 0.43 | n | 5 | n | n |
|  |  | 18:2794820 | - | CT/C | 1.24 | 2.12 | 0.56 | n | n | n | n |
|  |  | 18:2795114 | rs528297752 | C/T | 1.25 | 1.61 | 0.43 | 0.113 | 7 | n | n |
|  |  | 18:2795582 | rs138120280 | T/G | -0.66 | 1.44 | 0.65 | 0.384 | 3a | n | n |
|  |  | 18:2795721 | rs189706349 | A/C | 0.02 | 0.79 | 0.98 | 0.472 | 4 | n | n |
|  |  | 18:2796591 | rs779168930 | G/GT | 0.75 | 2.12 | 0.73 | n | 5 | n | n |
|  |  | 18:2796656 | rs144395718 | G/C | 1.05 | 0.83 | 0.20 | 0.238 | 5 | n | n |
|  |  | 18:2796706 | rs147416786 | G/A | 0.24 | 0.71 | 0.74 | 0.715 | 1f | SMCHD1 (0.00156331) | METTL4 (8.47E-10). NDC80(0.000002) |
|  |  | 18:2797093 | rs2076277772 | G/C | -2.33 | 2.13 | 0.27 | n | n | n | n |
|  |  | 18:2797207 | rs12960488 | G/T | 0.43 | 0.42 | 0.30 | 0.721 | 7 | n | n |
|  |  | 18:2797420 | rs117272425 | A/C | 0.24 | 0.71 | 0.74 | 0.715 | 7 | SMCHD1 (0.00156399) | METTL4 (1.21E-9). NDC80(0.0000023) |
|  |  | 18:2797491 | rs56170448 | G/A | -0.06 | 1.05 | 0.95 | 0.466 | 7 | n | n |
|  |  | 18:2797803 | rs150960954 | G/C | -1.69 | 0.38 | 9.39E-06 | 0.264 | 1f | METTL4 (0.00588108). SMCHD1 (0.0165408). MYL12B (0.0177765) | n |
| 20:55134001-55138000 | *DOK5* | 20:55134061 | rs79177272 | G/A | 0.02 | 2.13 | 0.99 | 0.789 | 7 | n | n |
|  |  | 20:55134114 | rs34307986 | A/C | 0.02 | 2.13 | 0.99 | 0.504 | 7 | n | n |
|  |  | 20:55134161 | rs6023893 | T/G | 0.02 | 2.13 | 0.99 | 0.504 | 7 | n | n |
|  |  | 20:55134232 | - | ACACACACACACG/A | 0.02 | 2.13 | 0.99 | n | n | n | n |
|  |  | 20:55134246 | rs992036971 | A/G | -1.81 | 2.12 | 0.39 | n | 5 | n | n |
|  |  | 20:55134343 | rs73624181 | C/T | -0.35 | 0.36 | 0.33 | 0.995 | 5 | n | n |
|  |  | 20:55134402 | rs993525560 | G/A | -1.78 | 2.12 | 0.40 | 0.370 | 5 | n | n |
|  |  | 20:55134454 | rs185867225 | A/G | -2.66 | 2.12 | 0.21 | n | 5 | n | n |
|  |  | 20:55134467 | rs6023895 | A/T | 0.02 | 2.13 | 0.99 | 0.506 | 5 | n | n |
|  |  | 20:55134513 | rs6023896 | G/A | 0.02 | 2.13 | 0.99 | n | 5 | n | n |
|  |  | 20:55134573 | rs570302005 | C/T | -1.57 | 1.21 | 0.20 | n | 7 | n | n |
|  |  | 20:55134634 | - | AACAC/A | -0.14 | 2.13 | 0.95 | n | n | n | n |
|  |  | 20:55134635 | - | ACACAG/A | 0.41 | 1.95 | 0.83 | n | n | n | n |
|  |  | 20:55134654 | rs533600421 | C/T | 0.85 | 1.91 | 0.66 | n | 7 | n | n |
|  |  | 20:55134699 | rs1173445232 | T/G | -2.10 | 0.50 | 3.19E-05 | n | 6 | n | n |
|  |  | 20:55134794 | rs1241819495 | T/G | -1.14 | 1.43 | 0.43 | n | 6 | n | n |
|  |  | 20:55134865 | rs553762042 | A/G | 0.09 | 1.20 | 0.94 | 0.776 | 6 | n | n |
|  |  | 20:55134953 | rs1432773304 | G/GA | -0.67 | 1.06 | 0.52 | n | n | n | n |
|  |  | 20:55134975 | rs1327092580 | T/A | 0.10 | 0.84 | 0.90 | n | 6 | n | n |
|  |  | 20:55135073 | rs141931052 | C/T | -0.49 | 1.03 | 0.64 | 0.723 | 6 | n | n |
|  |  | 20:55135103 | rs536195816 | T/G | 0.06 | 0.84 | 0.95 | n | 7 | n | n |
|  |  | 20:55135196 | rs147787114 | C/T | -2.46 | 2.12 | 0.25 | 0.760 | 7 | n | n |
|  |  | 20:55135310 | rs140186626 | A/T | -0.49 | 1.03 | 0.64 | 0.723 | 7 | n | n |
|  |  | 20:55136837 | rs189145100 | C/T | 0.89 | 0.86 | 0.30 | 0.978 | 3a | n | n |
|  |  | 20:55137147 | rs2426551 | T/G | 0.02 | 2.13 | 0.99 | 0.803 | 5 | n | n |
|  |  | 20:55137330 | rs566966131 | A/G | -0.39 | 1.21 | 0.75 | 0.508 | 7 | n | n |
|  |  | 20:55137402 | rs371006253 | T/TTG | -1.36 | 1.24 | 0.27 | 0.778 | 6 | n | n |
|  |  | 20:55137409 | rs183790092 | T/A | -2.31 | 2.12 | 0.28 | 0.642 | 7 | n | n |
|  |  | 20:55137577 | rs375202436 | A/G | -0.67 | 1.50 | 0.65 | n | 4 | n | n |
| 1:9236001-9240000 | *H6PD* | 1:9236242 | rs1640846082 | G/A | -2.07 | 2.12 | 0.33 | n | n | n | n |
|  |  | 1:9236400 | rs1004287298 | G/A | -2.34 | 2.12 | 0.27 | n | 4 | n | n |
|  |  | 1:9236771 | rs528487082 | A/G | -1.33 | 0.88 | 0.13 | 0.510 | 5 | n | n |
|  |  | 1:9236861 | rs140481872 | T/G | -1.93 | 1.12 | 0.084 | 0.697 | 3b | n | n |
|  |  | 1:9237068 | rs80066406 | C/T | -1.02 | 0.62 | 0.10 | 0.771 | 4 | n | n |
|  |  | 1:9237688 | rs1640891639 | A/G | -1.12 | 1.43 | 0.43 | n | n | n | n |
|  |  | 1:9237695 | - | GCT/G | -0.34 | 0.39 | 0.38 | 0.469 | n | n | n |
|  |  | 1:9237755 | - | A/G | -1.15 | 2.13 | 0.59 | n | n | n | n |
|  |  | 1:9237871 | rs1470106043 | G/A | 0.62 | 2.12 | 0.77 | n | 5 | n | n |
|  |  | 1:9237888 | rs561198981 | C/T | 0.97 | 2.11 | 0.65 | 0.302 | 5 | n | n |
|  |  | 1:9237981 | rs185732248 | G/A | 0.79 | 1.91 | 0.68 | 0.613 | 4 | n | n |
|  |  | 1:9238012 | rs190144830 | C/T | -1.21 | 0.73 | 0.095 | 0.033 | 4 | n | n |
|  |  | 1:9238084 | rs181041995 | A/G | -1.41 | 1.42 | 0.32 | 0.858 | 4 | n | n |
|  |  | 1:9238142 | - | TAGTG/T | -1.15 | 2.13 | 0.59 | 0.231 | n | n | n |
|  |  | 1:9238382 | - | CG/C | -1.48 | 0.87 | 0.091 | 0.290 | n | n | n |
|  |  | 1:9238988 | rs148541851 | A/T | -2.07 | 2.12 | 0.33 | n | 5 | n | n |
|  |  | 1:9239053 | rs34078161 | G/GT | -1.47 | 1.21 | 0.22 | n | 5 | n | n |
|  |  | 1:9239390 | rs780402980 | G/A | 1.31 | 2.13 | 0.54 | 0.989 | 4 | n | n |
|  |  | 1:9239774 | rs1381573205 | C/T | -1.76 | 2.12 | 0.40 | n | 2b | n | n |
|  |  | 1:9239780 | rs183081098 | G/A | -0.98 | 0.92 | 0.285 | 0.450 | 4 | n | n |
|  |  | 1:9239929 | rs966395316 | C/T | -2.04 | 2.12 | 0.34 | n | 4 | n | n |
| 8:6232001-6236000 | *MCPH1* | 8:6232158 | rs555191565 | T/C | -1.16 | 2.12 | 0.59 | 0.781 | 7 | n | n |
|  |  | 8:6232267 | rs1471705583 | A/T | -0.40 | 2.13 | 0.85 | 0.573 | 6 | n | n |
|  |  | 8:6232329 | rs1285135562 | G/T | 0.48 | 2.12 | 0.82 | n | 6 | n | n |
|  |  | 8:6232332 | rs546198145 | G/T | -1.37 | 2.12 | 0.52 | n | 6 | n | n |
|  |  | 8:6232395 | - | G/T | 0.66 | 2.12 | 0.75 | n | n | n | n |
|  |  | 8:6232494 | rs531210720 | G/A | -0.07 | 2.12 | 0.98 | 0.347 | 6 | n | n |
|  |  | 8:6232528 | rs548557181 | C/T | -1.04 | 0.49 | 0.035 | 0.240 | 7 | n | n |
|  |  | 8:6232642 | rs529868803 | A/C | -1.66 | 2.11 | 0.43 | 0.019 | 7 | n | n |
|  |  | 8:6232793 | rs79691026 | T/C | -0.67 | 0.41 | 0.10 | 0.244 | 7 | n | n |
|  |  | 8:6232842 | rs140784213 | G/A | -1.28 | 0.62 | 0.038 | 0.687 | 7 | n | n |
|  |  | 8:6232849 | rs149670511 | C/T | -2.61 | 2.12 | 0.22 | 0.224 | 6 | n | n |
|  |  | 8:6233177 | rs1384534652 | T/C | 0.06 | 2.12 | 0.98 | n | 7 | n | n |
|  |  | 8:6233726 | rs79601337 | T/A | -1.68 | 2.12 | 0.43 | 0.591 | 5 | n | n |
|  |  | 8:6233862 | rs998533584 | A/G | -0.29 | 2.12 | 0.89 | n | 6 | n | n |
|  |  | 8:6234230 | rs181802195 | T/A | -2.28 | 2.12 | 0.28 | 0.219 | 7 | n | n |
|  |  | 8:6234451 | rs116645364 | C/T | -2.17 | 1.91 | 0.25 | 0.913 | 7 | n | n |
|  |  | 8:6234521 | - | TA/T | 0.68 | 2.12 | 0.75 | n | n | n | n |
|  |  | 8:6234654 | rs111804510 | A/AT | -1.61 | 2.12 | 0.45 | n | 7 | n | n |
|  |  | 8:6234745 | rs573175087 | T/C | -2.69 | 2.13 | 0.21 | n | 5 | n | n |
|  |  | 8:6234766 | rs145958994 | C/T | -1.12 | 0.46 | 0.014 | 0.299 | 5 | n | n |
|  |  | 8:6234823 | rs954568843 | G/A | -2.96 | 2.12 | 0.16 | n | 5 | n | n |
|  |  | 8:6234917 | rs140131792 | G/A | -1.28 | 0.62 | 0.038 | 0.667 | 5 | n | n |
|  |  | 8:6234931 | rs1797278126 | G/C | 0.61 | 2.12 | 0.77 | n | n | n | n |
|  |  | 8:6235262 | - | TTG/T | -0.36 | 0.79 | 0.65 | n | n | n | n |
|  |  | 8:6235300 | rs149850270 | T/C | -0.67 | 0.41 | 0.10 | n | 5 | n | n |
|  |  | 8:6235447 | rs147092967 | C/T | -0.66 | 0.41 | 0.11 | 0.035 | 5 | n | n |
|  |  | 8:6235883 | rs978453653 | G/C | -1.15 | 2.13 | 0.59 | n | 7 | n | n |
| 4:131746001-131750000 | *PCDH10* | 4:131747008 | rs1240549703 | T/A | -0.47 | 2.12 | 0.82 | n | 7 | n | n |
|  |  | 4:131747016 | rs1406522297 | C/T | -0.10 | 0.30 | 0.75 | n | 7 | n | n |
|  |  | 4:131747018 | rs1177721152 | G/A | -0.37 | 2.12 | 0.86 | n | 7 | n | n |
|  |  | 4:131747024 | rs1334499306 | T/C | -0.37 | 2.12 | 0.86 | n | 7 | n | n |
|  |  | 4:131747031 | rs1447343344 | C/T | 0.85 | 0.58 | 0.14 | n | 7 | n | n |
|  |  | 4:131747032 | rs1278906043 | G/T | -0.31 | 0.88 | 0.72 | n | 7 | n | n |
|  |  | 4:131747038 | rs1348573582 | A/T | -0.06 | 0.34 | 0.87 | n | 7 | n | n |
|  |  | 4:131747056 | rs1031563432 | G/A | 0.47 | 0.43 | 0.27 | n | 7 | n | n |
|  |  | 4:131747064 | rs1468118128 | C/T | -0.84 | 0.73 | 0.25 | n | 7 | n | n |
|  |  | 4:131747068 | rs1469019199 | C/A | -0.62 | 1.04 | 0.55 | n | 7 | n | n |
|  |  | 4:131747092 | rs868253293 | A/T | -0.69 | 0.86 | 0.42 | n | 7 | n | n |
|  |  | 4:131747111 | rs1453882107 | C/G | 0.05 | 2.12 | 0.98 | n | 7 | n | n |
|  |  | 4:131747121 | rs1246716976 | C/A | -0.14 | 1.22 | 0.91 | n | 6 | n | n |
|  |  | 4:131747129 | rs1479833921 | G/A | 0.86 | 1.60 | 0.59 | n | 6 | n | n |
|  |  | 4:131747138 | rs1196779650 | G/C | -0.03 | 2.13 | 0.99 | n | 6 | n | n |
|  |  | 4:131747139 | rs1425372385 | G/C | -0.14 | 1.22 | 0.91 | n | 6 | n | n |
|  |  | 4:131747143 | rs1478008627 | C/T | -0.03 | 2.13 | 0.99 | n | 7 | n | n |
|  |  | 4:131747151 | rs866898230 | A/T | 0.81 | 0.41 | 0.048 | n | 7 | n | n |
|  |  | 4:131747156 | rs867674227 | G/A | 0.05 | 2.12 | 0.98 | n | 7 | n | n |
|  |  | 4:131747235 | rs1411917030 | A/G | 0.54 | 1.94 | 0.78 | n | 7 | n | n |
|  |  | 4:131747245 | rs1316516651 | T/C | 1.86 | 1.98 | 0.35 | n | 7 | n | n |
|  |  | 4:131747247 | rs1342589093 | G/C | 0.96 | 0.89 | 0.28 | n | 7 | n | n |
|  |  | 4:131747250 | rs1449261251 | C/A | -1.37 | 2.12 | 0.52 | n | 7 | n | n |
|  |  | 4:131747251 | rs1314927422 | C/T | -0.25 | 0.36 | 0.49 | n | 7 | n | n |
|  |  | 4:131747283 | rs1160527076 | G/T | -1.20 | 0.54 | 0.027 | n | 5 | n | n |
|  |  | 4:131747302 | rs1258545227 | C/T | 0.05 | 2.12 | 0.98 | n | 5 | n | n |
|  |  | 4:131747303 | rs1346743852 | C/T | 0.05 | 2.12 | 0.98 | n | 5 | n | n |
|  |  | 4:131747306 | rs1262344216 | G/C | 0.18 | 1.05 | 0.86 | n | 5 | n | n |
|  |  | 4:131747308 | rs878949847 | G/T | 0.11 | 0.36 | 0.77 | n | 5 | n | n |
|  |  | 4:131747317 | rs1404294848 | C/A | 0.10 | 1.91 | 0.96 | n | 5 | n | n |
|  |  | 4:131747360 | rs1302113404 | C/G | -2.13 | 2.12 | 0.31 | n | 5 | n | n |
|  |  | 4:131747363 | - | AGCC/A | -2.13 | 2.12 | 0.31 | n | n | n | n |
|  |  | 4:131747378 | rs879283396 | G/A | -0.85 | 1.43 | 0.55 | n | 5 | n | n |
|  |  | 4:131747411 | rs1216694803 | C/T | 0.09 | 1.00 | 0.93 | n | 5 | n | n |
|  |  | 4:131747424 | rs1444634228 | G/C | 0.13 | 0.70 | 0.86 | n | 5 | n | n |
|  |  | 4:131747434 | rs1344276830 | G/A | -0.44 | 0.87 | 0.62 | n | 5 | n | n |
|  |  | 4:131747454 | rs1224434924 | C/T | 0.07 | 0.37 | 0.85 | n | 5 | n | n |
|  |  | 4:131747462 | rs1212679555 | C/A | -0.37 | 1.22 | 0.76 | n | 5 | n | n |
|  |  | 4:131747464 | rs1264070822 | A/T | 0.01 | 0.38 | 0.97 | n | 5 | n | n |
|  |  | 4:131747465 | rs1461159151 | G/T | -0.25 | 0.42 | 0.55 | n | 5 | n | n |
|  |  | 4:131747468 | rs1240608290 | C/T | -0.42 | 0.55 | 0.45 | n | 5 | n | n |
|  |  | 4:131747469 | rs1470863340 | C/A | 1.16 | 0.79 | 0.14 | n | 5 | n | n |
|  |  | 4:131747474 | rs77527335 | C/T | -0.29 | 0.44 | 0.52 | n | 5 | n | n |
|  |  | 4:131747475 | rs1406768673 | G/A | 0.39 | 0.34 | 0.25 | n | 5 | n | n |
|  |  | 4:131747478 | rs1178147730 | G/T | -0.37 | 1.22 | 0.76 | n | 5 | n | n |
|  |  | 4:131747479 | rs1406469967 | C/A | -0.22 | 0.44 | 0.61 | n | 5 | n | n |
|  |  | 4:131747481 | rs1334559850 | T/G | 1.22 | 0.77 | 0.11 | n | 7 | n | n |
|  |  | 4:131747491 | rs1323231035 | T/G | -0.88 | 0.54 | 0.10 | n | 7 | n | n |
|  |  | 4:131747524 | - | A/C | -1.93 | 2.12 | 0.36 | n | n | n | n |
|  |  | 4:131747533 | rs867796620 | G/A | 0.43 | 1.12 | 0.70 | n | 7 | n | n |
|  |  | 4:131747570 | rs1320545770 | G/A | 0.37 | 2.12 | 0.86 | n | 5 | n | n |
|  |  | 4:131747584 | rs1285953326 | C/G | 0.51 | 1.92 | 0.79 | n | 5 | n | n |
|  |  | 4:131747589 | rs377133773 | C/T | -0.54 | 0.46 | 0.24 | n | 5 | n | n |
|  |  | 4:131747644 | rs371056470 | G/A | 0.00 | 0.34 | 0.99 | n | 7 | n | n |
|  |  | 4:131747657 | rs1408698138 | C/G | 0.04 | 0.35 | 0.90 | n | 6 | n | n |
|  |  | 4:131747673 | rs1325367072 | G/T | 0.21 | 2.12 | 0.92 | n | 7 | n | n |
|  |  | 4:131747681 | rs796371069 | G/A | -0.15 | 0.35 | 0.67 | n | 7 | n | n |
|  |  | 4:131747688 | rs866904362 | C/T | 0.65 | 0.70 | 0.36 | n | 7 | n | n |
|  |  | 4:131747701 | rs1305761826 | G/T | -0.06 | 0.40 | 0.87 | n | 6 | n | n |
|  |  | 4:131747703 | rs1347058193 | G/T | 0.10 | 1.91 | 0.96 | n | 7 | n | n |
|  |  | 4:131747704 | rs1405315626 | C/A | -0.07 | 0.63 | 0.91 | n | 7 | n | n |
|  |  | 4:131747711 | rs1271306046 | G/A | 0.69 | 2.12 | 0.74 | n | 7 | n | n |
|  |  | 4:131747712 | rs374111106 | G/A | -0.18 | 0.38 | 0.63 | n | 7 | n | n |
|  |  | 4:131747714 | rs1198975908 | G/A | 0.77 | 2.12 | 0.72 | n | 7 | n | n |
|  |  | 4:131747717 | rs531053225 | C/A | 0.77 | 2.12 | 0.715 | n | 7 | n | n |
|  |  | 4:131747721 | rs1166237225 | T/G | 1.44 | 1.48 | 0.33 | n | 7 | n | n |
|  |  | 4:131747725 | rs1405117838 | T/A | -0.48 | 1.21 | 0.69 | 0.512 | 7 | n | n |
|  |  | 4:131747729 | rs1302172754 | C/T | -0.10 | 0.55 | 0.86 | n | 7 | n | n |
|  |  | 4:131747731 | rs755326584 | C/T | -0.13 | 0.40 | 0.75 | n | 7 | n | n |
|  |  | 4:131747734 | rs1296184439 | A/T | -0.26 | 0.36 | 0.46 | n | 7 | n | n |
|  |  | 4:131747738 | rs1293962546 | A/G | 0.48 | 0.62 | 0.44 | n | 7 | n | n |
|  |  | 4:131747745 | rs1487034164 | T/C | 1.36 | 2.00 | 0.50 | n | 7 | n | n |
|  |  | 4:131747750 | rs1185967934 | A/C | -1.17 | 0.66 | 0.076 | n | 7 | n | n |
|  |  | 4:131747773 | rs569798623 | G/T | -0.01 | 0.50 | 0.99 | n | 7 | n | n |
|  |  | 4:131747784 | rs1260438873 | C/A | 0.14 | 0.82 | 0.86 | n | 7 | n | n |
|  |  | 4:131747787 | rs1463841907 | C/T | -0.72 | 1.23 | 0.56 | n | 7 | n | n |
|  |  | 4:131747814 | rs1406796533 | G/A | -1.16 | 0.56 | 0.039 | n | 7 | n | n |
|  |  | 4:131747832 | rs1327486952 | G/T | 1.14 | 1.60 | 0.48 | n | 7 | n | n |
|  |  | 4:131747833 | rs1225789254 | C/G | 1.14 | 1.60 | 0.48 | n | 7 | n | n |
|  |  | 4:131747866 | rs140913111 | C/T | 0.22 | 0.45 | 0.62 | n | 7 | n | n |
|  |  | 4:131747868 | rs879861724 | G/C | -0.08 | 0.73 | 0.91 | n | 7 | n | n |
|  |  | 4:131747911 | rs546704521 | G/C | 0.02 | 0.71 | 0.98 | n | 6 | n | n |
|  |  | 4:131747971 | - | GTCTCTC/G | -0.73 | 1.43 | 0.61 | n | n | n | n |
|  |  | 4:131747983 | - | C/A | 0.15 | 2.12 | 0.94 | n | n | n | n |
|  |  | 4:131748079 | rs1252863035 | G/C | 0.01 | 2.12 | 1.00 | n | 7 | n | n |
|  |  | 4:131748122 | rs1330442140 | C/T | 0.18 | 0.35 | 0.61 | n | 7 | n | n |
|  |  | 4:131748144 | rs1197679600 | T/A | NA | NA | NA | n | 7 | n | n |
|  |  | 4:131748173 | rs1300477498 | G/T | 0.96 | 2.11 | 0.65 | n | 7 | n | n |
|  |  | 4:131748180 | rs1229523846 | G/T | -1.85 | 2.13 | 0.38 | n | 6 | n | n |
|  |  | 4:131749044 | rs370404878 | G/C | -0.02 | 0.44 | 0.96 | n | 7 | n | n |
|  |  | 4:131749049 | rs1434199075 | G/T | -1.67 | 0.87 | 0.056 | n | 7 | n | n |
|  |  | 4:131749071 | rs1428867552 | G/A | 0.63 | 1.74 | 0.72 | n | 7 | n | n |
|  |  | 4:131749091 | rs866524697 | G/C | 0.33 | 0.54 | 0.54 | n | 6 | n | n |
|  |  | 4:131749112 | rs868709244 | G/C | 0.87 | 0.55 | 0.12 | n | 7 | n | n |
|  |  | 4:131749114 | rs1190024389 | G/T | 0.27 | 0.60 | 0.65 | n | 7 | n | n |
|  |  | 4:131749124 | rs1167909308 | C/A | -0.54 | 1.23 | 0.66 | n | 7 | n | n |
|  |  | 4:131749149 | rs1364571848 | C/G | -2.47 | 2.12 | 0.24 | n | 7 | n | n |
|  |  | 4:131749161 | rs563341443 | C/T | -0.37 | 2.12 | 0.86 | n | 7 | n | n |
|  |  | 4:131749215 | rs1291761377 | G/A | -2.08 | 2.12 | 0.33 | n | 7 | n | n |
|  |  | 4:131749270 | rs1302700831 | C/T | -0.62 | 0.54 | 0.25 | n | 6 | n | n |
|  |  | 4:131749293 | rs1425911860 | C/T | -0.19 | 0.42 | 0.65 | n | 7 | n | n |
|  |  | 4:131749316 | rs865886099 | G/C | 0.16 | 0.31 | 0.60 | n | 7 | n | n |
|  |  | 4:131749335 | - | G/C | 0.58 | 2.12 | 0.78 | n | n | n | n |
|  |  | 4:131749355 | rs1439350913 | G/A | 0.41 | 0.43 | 0.34 | n | 5 | n | n |
|  |  | 4:131749363 | - | CTCTG/C | -0.58 | 0.88 | 0.51 | n | n | n | n |
|  |  | 4:131749377 | rs1465433091 | G/C | -1.56 | 1.91 | 0.41 | n | 5 | n | n |
|  |  | 4:131749419 | rs1470196983 | G/A | -1.06 | 0.69 | 0.13 | n | 5 | n | n |
|  |  | 4:131749422 | rs1382167786 | C/G | -0.03 | 0.34 | 0.94 | n | 5 | n | n |
|  |  | 4:131749424 | rs1415778085 | T/C | -0.03 | 0.34 | 0.94 | n | 5 | n | n |
|  |  | 4:131749425 | rs1313150853 | G/C | 1.21 | 1.60 | 0.45 | n | 5 | n | n |
|  |  | 4:131749499 | rs78665001 | C/T | 0.85 | 0.57 | 0.14 | n | 5 | n | n |
|  |  | 4:131749535 | rs1326192679 | G/A | -0.20 | 0.76 | 0.80 | n | 5 | n | n |
|  |  | 4:131749540 | rs1213640973 | G/A | 0.49 | 1.91 | 0.80 | n | 5 | n | n |
|  |  | 4:131749553 | rs1262063566 | C/G | -0.35 | 1.21 | 0.77 | n | 7 | n | n |
|  |  | 4:131749555 | rs1190678320 | G/T | -0.97 | 0.84 | 0.25 | n | 7 | n | n |
|  |  | 4:131749559 | rs1419781440 | C/G | 0.00 | 0.68 | 1.00 | n | 7 | n | n |
|  |  | 4:131749562 | - | T/A | 0.10 | 0.37 | 0.78 | n | n | n | n |
|  |  | 4:131749586 | rs1485309860 | A/T | -0.11 | 2.12 | 0.96 | n | 7 | n | n |
|  |  | 4:131749592 | rs1415664832 | T/G | 0.91 | 1.93 | 0.64 | n | 6 | n | n |
|  |  | 4:131749593 | rs1420810332 | G/T | -2.34 | 1.99 | 0.24 | n | 6 | n | n |
|  |  | 4:131749595 | rs1363321579 | G/A | 0.17 | 0.57 | 0.77 | n | 6 | n | n |
|  |  | 4:131749600 | rs1451844690 | A/G | 0.06 | 0.82 | 0.94 | n | 6 | n | n |
|  |  | 4:131749604 | rs80015978 | T/A | 0.92 | 2.12 | 0.66 | n | 6 | n | n |
|  |  | 4:131749622 | - | C/T | 0.20 | 2.12 | 0.92 | n | n | n | n |
|  |  | 4:131749625 | rs369792293 | C/T | 0.07 | 0.41 | 0.86 | n | 6 | n | n |
|  |  | 4:131749632 | rs1395990978 | C/G | -0.55 | 0.93 | 0.56 | n | 6 | n | n |
|  |  | 4:131749648 | rs1277872298 | G/A | 1.16 | 1.62 | 0.47 | n | 7 | n | n |
|  |  | 4:131749663 | rs1195506473 | G/A | -0.34 | 0.65 | 0.60 | n | 7 | n | n |
|  |  | 4:131749664 | rs78837673 | G/C | -0.05 | 0.32 | 0.87 | n | 6 | n | n |
|  |  | 4:131749665 | rs1453957224 | A/T | -0.14 | 0.54 | 0.79 | n | 6 | n | n |
|  |  | 4:131749700 | rs1315927058 | G/A | -0.22 | 1.24 | 0.86 | n | 7 | n | n |
|  |  | 4:131749715 | rs1186231929 | G/C | 0.31 | 0.38 | 0.425 | n | 7 | n | n |
|  |  | 4:131749728 | rs879276485 | G/A | 0.72 | 0.94 | 0.445 | n | 7 | n | n |
|  |  | 4:131749735 | rs1431031177 | G/A | -3.00 | 1.49 | 0.044 | n | 7 | n | n |
|  |  | 4:131749738 | rs1269330507 | G/T | 1.23 | 1.76 | 0.49 | n | 7 | n | n |
|  |  | 4:131749740 | rs1338190892 | C/G | 0.17 | 0.82 | 0.84 | n | 7 | n | n |
|  |  | 4:131749741 | rs1235031023 | C/A | -2.87 | 0.67 | 2.03E-05 | n | 7 | n | n |
|  |  | 4:131749751 | rs1286441455 | G/T | -1.08 | 1.24 | 0.38 | n | 7 | n | n |
|  |  | 4:131749763 | rs1417858606 | C/G | -0.71 | 1.20 | 0.56 | n | 7 | n | n |
|  |  | 4:131749768 | rs370988833 | C/G | 0.00 | 0.41 | 0.99 | n | 6 | n | n |
|  |  | 4:131749769 | rs1400892268 | G/T | -0.65 | 1.05 | 0.54 | n | 6 | n | n |
|  |  | 4:131749777 | rs1242539574 | C/T | 0.33 | 0.33 | 0.31 | n | 6 | n | n |
|  |  | 4:131749781 | rs1345652002 | G/A | -0.15 | 0.37 | 0.69 | n | 7 | n | n |
|  |  | 4:131749784 | rs1223411311 | G/A | 1.12 | 1.74 | 0.52 | n | 7 | n | n |
|  |  | 4:131749790 | rs1257278310 | G/T | 0.04 | 0.44 | 0.92 | n | 7 | n | n |
|  |  | 4:131749796 | rs1470246944 | G/A | 0.57 | 0.55 | 0.30 | n | 7 | n | n |
|  |  | 4:131749797 | rs1157623401 | C/G | -2.30 | 2.12 | 0.28 | n | 7 | n | n |
|  |  | 4:131749800 | rs1436209856 | C/A | 0.79 | 0.69 | 0.25 | n | 7 | n | n |
|  |  | 4:131749816 | rs1441650589 | C/T | 1.51 | 1.82 | 0.41 | n | 7 | n | n |
|  |  | 4:131749817 | rs1307184415 | G/A | 0.81 | 0.54 | 0.13 | n | 7 | n | n |
|  |  | 4:131749828 | rs1437904016 | C/A | 0.31 | 1.06 | 0.77 | n | 7 | n | n |
|  |  | 4:131749846 | rs868376013 | G/A | 0.12 | 0.41 | 0.77 | n | 6 | n | n |
|  |  | 4:131749856 | rs1230170151 | A/T | 0.87 | 0.94 | 0.35 | n | 6 | n | n |
|  |  | 4:131749865 | rs1469456602 | G/A | 0.89 | 0.56 | 0.11 | n | 7 | n | n |
|  |  | 4:131749878 | rs865906033 | G/C | 0.52 | 0.75 | 0.49 | n | 6 | n | n |
|  |  | 4:131749892 | rs1217311879 | G/C | 0.47 | 1.05 | 0.65 | n | 6 | n | n |
|  |  | 4:131749894 | - | T/A | 0.33 | 2.12 | 0.88 | n | n | n | n |
|  |  | 4:131749909 | rs1487869941 | G/A | 0.04 | 2.12 | 0.98 | n | 5 | n | n |
|  |  | 4:131749911 | rs1420024826 | C/A | 0.73 | 2.12 | 0.73 | n | 5 | n | n |
|  |  | 4:131749916 | rs1366825046 | C/G | -0.62 | 0.62 | 0.32 | n | 5 | n | n |
|  |  | 4:131749930 | rs1356595813 | C/A | -0.19 | 2.12 | 0.93 | n | 5 | n | n |
|  |  | 4:131749935 | rs1261012690 | C/T | -0.19 | 2.12 | 0.93 | n | 5 | n | n |
|  |  | 4:131749942 | rs1421015566 | C/G | -0.38 | 1.42 | 0.79 | n | 5 | n | n |
|  |  | 4:131749954 | rs1376331087 | C/G | -1.19 | 1.42 | 0.40 | n | 5 | n | n |
|  |  | 4:131749970 | rs1282155455 | G/A | -1.68 | 2.12 | 0.43 | n | 5 | n | n |
|  |  | 4:131749985 | rs75107596 | G/T | 0.43 | 0.36 | 0.23 | n | 5 | n | n |
|  |  | 4:131750000 | rs74453729 | C/T | 0.21 | 0.53 | 0.70 | n | 5 | n | n |
| 9:119000001-119004000 | *BRINP1* | 9:119000392 | rs185750238 | G/A | -0.71 | 1.42 | 0.62 | 0.932 | 7 | n | n |
|  |  | 9:119000635 | rs1023062562 | C/T | 0.17 | 0.78 | 0.83 | 0.989 | 7 | n | n |
|  |  | 9:119000804 | rs931022090 | C/T | -1.35 | 2.12 | 0.53 | n | 7 | n | n |
|  |  | 9:119000966 | rs116851645 | C/T | -0.70 | 0.94 | 0.46 | 0.515 | 5 | n | n |
|  |  | 9:119001163 | - | C/A | -2.38 | 2.12 | 0.26 | n | n | n | n |
|  |  | 9:119001227 | - | C/T | -2.38 | 2.12 | 0.26 | n | n | n | n |
|  |  | 9:119001344 | rs117139412 | A/G | -1.37 | 1.25 | 0.27 | 0.264 | 4 | n | n |
|  |  | 9:119001378 | rs138088873 | T/A | -1.06 | 0.87 | 0.22 | 0.509 | 4 | n | n |
|  |  | 9:119001445 | rs1179291497 | C/T | 0.71 | 2.13 | 0.74 | n | 4 | n | n |
|  |  | 9:119001468 | - | GCA/G | -2.03 | 2.12 | 0.34 | n | n | n | n |
|  |  | 9:119001597 | rs1284955665 | G/T | -0.47 | 1.42 | 0.74 | n | 7 | n | n |
|  |  | 9:119001778 | rs186873336 | A/G | 0.65 | 2.12 | 0.76 | 0.546 | 7 | n | n |
|  |  | 9:119002084 | rs1382078436 | A/G | -1.64 | 2.11 | 0.44 | n | 7 | n | n |
|  |  | 9:119002187 | rs117160909 | G/A | -1.06 | 0.87 | 0.22 | n | 7 | n | n |
|  |  | 9:119002226 | rs1245745621 | G/T | -0.79 | 0.93 | 0.39 | n | 7 | n | n |
|  |  | 9:119002276 | rs545851934 | C/T | -0.13 | 1.21 | 0.91 | n | 6 | n | n |
|  |  | 9:119002428 | rs139336758 | C/T | -2.93 | 0.80 | 0.00027 | 0.601 | 5 | n | n |
|  |  | 9:119002450 | rs554144524 | G/A | -2.87 | 2.12 | 0.18 | 0.560 | 5 | n | n |
|  |  | 9:119002460 | rs896747544 | G/A | -0.28 | 2.12 | 0.89 | n | 5 | n | n |
|  |  | 9:119002603 | rs182476435 | C/T | 2.00 | 2.14 | 0.35 | n | 4 | n | n |
|  |  | 9:119002828 | rs748928043 | A/G | 0.08 | 0.66 | 0.91 | 0.955 | 5 | n | n |
|  |  | 9:119003058 | rs533715012 | G/A | -1.25 | 1.42 | 0.38 | n | 6 | n | n |
|  |  | 9:119003180 | rs78525081 | C/T | -2.64 | 1.74 | 0.13 | 0.022 | 4 | n | n |
|  |  | 9:119003242 | rs118145571 | A/T | 0.56 | 0.32 | 0.086 | 0.921 | 4 | n | n |
|  |  | 9:119003303 | rs193164465 | G/A | 0.55 | 2.12 | 0.79 | n | 4 | n | n |
|  |  | 9:119003376 | - | G/T | -1.06 | 0.87 | 0.22 | n | n | n | n |
| 18:880001-884000 | *ADCYAP1* | 18:880323 | rs570905783 | C/T | -0.57 | 0.95 | 0.55 | 0.987 | 7 | n | n |
|  |  | 18:880758 | - | A/C | -0.05 | 2.12 | 0.98 | n | n | n | n |
|  |  | 18:880995 | rs186354423 | T/C | -2.94 | 1.60 | 0.07 | 0.942 | 7 | n | n |
|  |  | 18:881067 | rs534273948 | A/G | -1.26 | 2.12 | 0.55 | n | 5 | n | n |
|  |  | 18:881378 | rs1389965142 | C/G | 1.90 | 2.14 | 0.37 | 0.592 | 5 | n | n |
|  |  | 18:881496 | rs1383451013 | T/TGC | -1.68 | 2.12 | 0.43 | n | 6 | n | n |
|  |  | 18:881618 | rs535330644 | G/A | -2.21 | 2.13 | 0.30 | n | 7 | n | n |
|  |  | 18:881703 | rs117357962 | T/C | -1.03 | 0.35 | 0.0029 | 0.216 | 7 | THOC1 (0.0479236). TYMSOS (0.0113349). ENOSF1(0.03734) | ENOSF1(1.32E-26) |
|  |  | 18:881714 | rs13381802 | G/C | -1.68 | 0.70 | 0.016 | 0.545 | 7 | n | n |
|  |  | 18:882898 | rs747961623 | G/A | -2.46 | 2.12 | 0.25 | n | 6 | n | n |
|  |  | 18:882949 | rs1171099809 | C/T | -2.46 | 2.12 | 0.25 | n | 7 | n | n |
|  |  | 18:882965 | rs139632677 | C/T | 1.05 | 1.90 | 0.58 | 0.135 | 7 | n | n |
|  |  | 18:882974 | rs1403018448 | G/A | -0.92 | 1.47 | 0.53 | n | 7 | n | n |
|  |  | 18:883065 | rs1212991255 | C/T | -0.71 | 1.21 | 0.56 | n | 7 | n | n |
|  |  | 18:883067 | rs1268306360 | G/A | -0.28 | 2.12 | 0.89 | n | 7 | n | n |
|  |  | 18:883635 | rs749187530 | T/C | 0.74 | 2.12 | 0.73 | n | 5 | n | n |
| 20:55132001-55136000 | *DOK5* | 20:55132282 | - | T/TA | 0.88 | 2.12 | 0.68 | n | n | n | n |
|  |  | 20:55133272 | rs781055852 | A/G | -0.97 | 1.48 | 0.51 | n | 4 | n | n |
|  |  | 20:55133297 | rs75940426 | G/A | -0.85 | 0.66 | 0.19 | n | 4 | n | n |
|  |  | 20:55133322 | rs1359950011 | C/T | -1.01 | 1.47 | 0.49 | n | 4 | n | n |
|  |  | 20:55133418 | rs962986516 | A/G | 0.65 | 2.12 | 0.76 | n | 4 | n | n |
|  |  | 20:55133507 | rs6023892 | A/G | 0.02 | 2.13 | 0.99 | 0.504 | 4 | n | n |
|  |  | 20:55133862 | rs1473312744 | C/T | 1.09 | 1.98 | 0.58 | n | 5 | n | n |
|  |  | 20:55133880 | rs559304211 | G/A | -0.68 | 0.63 | 0.28 | 0.159 | 5 | n | n |

Abbreviations: REF. Reference allele; ALT. Alternative allele; SE. Standard error.

**RegulomeDB ranking score**

Score Supporting data

1a eQTL/caQTL + TF binding + matched TF motif + matched Footprint + chromatin accessibility peak

1b eQTL/caQTL + TF binding + any motif + Footprint + chromatin accessibility peak

1c eQTL/caQTL + TF binding + matched TF motif + chromatin accessibility peak

1d eQTL/caQTL + TF binding + any motif + chromatin accessibility peak

1e eQTL/caQTL + TF binding + matched TF motif

1f eQTL/caQTL + TF binding / chromatin accessibility peak

2a TF binding + matched TF motif + matched Footprint + chromatin accessibility peak

2b TF binding + any motif + Footprint + chromatin accessibility peak

2c TF binding + matched TF motif + chromatin accessibility peak

3a TF binding + any motif + chromatin accessibility peak

3b TF binding + matched TF motif

4 TF binding + chromatin accessibility peak

5 TF binding or chromatin accessibility peak

6 Motif hit

7 Other

## **Supplementary Table S8. Variants in the discovered enhancers (STAAR-O) with replication in type 1 diabetes (FinnDiane) and available Retina eQTL data and eQTLgen**

| **Closest gene** | **CHR** | **Position** | **REF/ALT** | **Variant** | **Beta/Effect** | **SE** | **Pvalue** | **FinnDiane GWAS** | **Regulome DB** | **Retina eQTL target gene** | **eQTLgen** |
| --- | --- | --- | --- | --- | --- | --- | --- | --- | --- | --- | --- |
| *RETREG1* | 5 | 16540476 | G/A | rs1741209432 | -0.06 | 2.12 | 0.98 | - | 0.6091 (4) | - | - |
|  | 5 | 16540633 | G/A | rs10053126 | -1.50 | 0.38 | 9.39E-05 | 0.285 | 0.6091 (4) | - | - |
| *CBX4* | 17 | 79841603 | C/A | rs1232612896 | -2.07 | 2.12 | 0.33 | - | 0.998 (2b) | - | - |
|  | 17 | 79841664 | T/C | rs62075212 | -1.23 | 0.33 | 0.0002 | - | 0.6091 (4) | RP11-353N14.2 (Long noncoding RNA) (p=0.01).  CBX2 (p=0.04). CCDC40 (p=0.03) | - |

Abbreviations: REF. Reference allele; ALT. Alternative allele; SE. Standard error.

**RegulomeDB ranking score**

Score Supporting data

1a eQTL/caQTL + TF binding + matched TF motif + matched Footprint + chromatin accessibility peak

1b eQTL/caQTL + TF binding + any motif + Footprint + chromatin accessibility peak

1c eQTL/caQTL + TF binding + matched TF motif + chromatin accessibility peak

1d eQTL/caQTL + TF binding + any motif + chromatin accessibility peak

1e eQTL/caQTL + TF binding + matched TF motif

1f eQTL/caQTL + TF binding / chromatin accessibility peak

2a TF binding + matched TF motif + matched Footprint + chromatin accessibility peak

2b TF binding + any motif + Footprint + chromatin accessibility peak

2c TF binding + matched TF motif + chromatin accessibility peak

3a TF binding + any motif + chromatin accessibility peak

3b TF binding + matched TF motif

4 TF binding + chromatin accessibility peak

5 TF binding or chromatin accessibility peak

6 Motif hit

7 Other

## **Supplementary Table S9. Variants in the discovered promoters (STAAR-O) with replication in type 1 diabetes (FinnDiane) and available Retina eQTL data and eQTLgen**

| **Closest gene** | **CHR** | **Position** | **REF/ALT** | **Variant** | **Beta** | **SE** | **Pvalue** | **FinnDiane GWAS** | **RegulomeDB** | **Retina eQTL target gene** | **eQTLgen** |
| --- | --- | --- | --- | --- | --- | --- | --- | --- | --- | --- | --- |
| *PCBP4* | 3 | 51958592 | G/A | rs1699949395 | -2.22 | 2.13 | 0.29717 | n | n | n | n |
|  | 3 | 51958684 | G/A | rs1699958136 | -2.21 | 2.13 | 0.29858 | n | n | n | n |
|  | 3 | 51958800 | T/A | n | -2.64 | 2.12 | 0.21225 | n | n | n | n |
|  | 3 | 51959009 | C/T | rs111526907 | -2.33 | 2.13 | 0.27358 | 0.47196 | 0.23589 (5) | n | n |
|  | 3 | 51959215 | C/G | rs151276928 | -1.78 | 0.72 | **0.01306** | 0.68170 | 0.55426 (1f) | n | ITIH4 (0.0000014) |
| *PDS5A* | 4 | 39961214 | T/C | rs4627876 | -2.71 | 1.94 | 0.16187 | 0.47487 | 0.58955 (5) | n | n |
|  | 4 | 39961360 | G/A | rs771767045 | -3.02 | 2.12 | 0.15364 | n | 0.67778 (3a) | n | n |
|  | 4 | 39961695 | T/G | rs1729335361 | -2.80 | 2.12 | 0.18761 | n | n | n | n |
|  | 4 | 39962074 | T/TC | rs1729528230 | -2.81 | 2.13 | 0.18652 | n | n | n | n |
| *RETREG1* | 5 | 16539662 | T/C | rs980849915 | -1.52 | 0.9297 | 0.10158 | 0.15012 | n | n | n |
|  | 5 | 16539697 | T/C | rs16868760 | -1.50 | 0.3836 | **9.39E-05** | 0.41701 | n | n | n |
|  | 5 | 16540212 | T/C | rs1002179285 | 0.95 | 2.1158 | 0.65378 | n | n | n | n |
|  | 5 | 16540425 | G/A | rs1741208414 | 0.19 | 2.1215 | 0.92998 | n | n | n | n |
|  | 5 | 16540476 | G/A | rs1741209432 | -0.05 | 2.1174 | 0.97935 | n | n | n | n |
|  | 5 | 16540633 | G/A | rs10053126 | -1.50 | 0.3836 | **9.39E-05** | 0.28524 | 0.60906 (4) | n | n |
| *TMOD2* | 15 | 38130606 | C/A | rs147304347 | -0.33 | 0.4068 | 0.42016 | 0.80787 | 0.55436 (1f) | n | n |
|  | 15 | 38130620 | A/G | rs1446475173 | -0.14 | 2.1187 | 0.94577 | n | 0.78855 (2b) | n | n |
|  | 15 | 38130710 | G/A | rs1236778760 | 0.81 | 1.9463 | 0.67618 | n | 0.60906 (4) | n | n |
|  | 15 | 38130711 | C/T | rs1483522340 | 0.82 | 1.9490 | 0.67505 | n | 0.60906 (4) | n | n |
|  | 15 | 38131181 | C/T | rs139408279 | -1.34 | 0.3243 | **3.36E-05** | 0.3413 | 0.55436 (1f) | n | SPRED1(2.676E-18). RASGRP1(0.0000058) |
|  | 15 | 38131505 | T/C | rs962432539 | -2.85 | 2.1198 | 0.17862 | n | 0.70497 (4) | n | n |
| *ACTRT2* | 1 | 3020479 | C/T | rs187533623 | -1.33 | 1.2175 | 0.27584 | 0.36042 | 0.49614 (2b) | n | n |
|  | 1 | 3020549 | G/T | rs192458590 | -0.97 | 0.3725 | **0.00939** | 0.64404 | 0.60906 (4) | n | PLCH2(1.6214E-8) |
|  | 1 | 3020550 | A/T | rs184000772 | -0.97 | 0.3725 | **0.00939** | 0.64404 | 0.60906 (4) | n | n |
|  | 1 | 3020664 | G/A | rs61266721 | -2.71 | 2.119 | 0.20156 | 0.77242 | 0.13454 (5) | n | n |
|  | 1 | 3020920 | T/C | rs569320535 | -1.36 | 0.74376 | 0.06665 | 0.17830 | 0.8288 (2b) | n | n |
|  | 1 | 3021120 | A/G | rs139157059 | -2.89 | 2.1182 | 0.17246 | n | 0.13454 (5) | n | n |
| *APBB1IP* | 10 | 26566160 | C/T | rs183098934 | -0.15 | 2.1292 | 0.94480 | n | 0.58955 (5) | n | n |
|  | 10 | 26566206 | G/A | rs528416532 | 0.86 | 2.1197 | 0.68352 | n | 0.58955 (5) | n | n |
|  | 10 | 26566234 | C/A | rs974793092 | 0.9991 | 1.4860 | 0.50137 | 0.8420 | 0.58955 (5) | n | n |
|  | 10 | 26566265 | T/G | rs10829025 | -1.85 | 0.4934 | **0.00018** | 0.7715 | 0.58955 (5) | ACBD5 (0.05) | LINC00264(3.3929E-7) |
|  | 10 | 26566647 | G/A | rs952875383 | 0.68 | 2.1175 | 0.74715 | n | 0.60906 (4) | n | n |
|  | 10 | 26567075 | G/T | rs200232575 | 0.75 | 0.7330 | 0.30770 | 0.35021 | 0.60906 (4) | n | n |
| *TES* | 7 | 116239386 | A/G | rs192056382 | -2.14 | 0.6734 | **0.00148** | 0.10433 | 0.60906 (4) | n | n |
|  | 7 | 116239579 | T/C | rs144416471 | -0.94 | 0.4100 | **0.02174** | 0.48389 | 0.55436 (1f) | n | CAV2(0.0000051) |
| *PTGER2* | 14 | 52313890 | G/C | rs377379323 | -2.62 | 1.9105 | 0.17028 | 0.70018 | 0.60906 (4) | n | n |
|  | 14 | 52314314 | G/T | rs542981572 | -0.05 | 2.1175 | 0.97977 | n | 0.60906 (4) | n | n |
|  | 14 | 52314326 | G/A | rs2033807334 | -2.76 | 2.1170 | 0.19273 | n | n | n | n |
|  | 14 | 52314340 | G/T | rs1171492748 | -2.44 | 2.1154 | 0.24944 | n | 0.60906 (4) | n | n |
|  | 14 | 52314436 | T/G | n | -2.07 | 2.138 | 0.33204 | n | n | n | n |
|  | 14 | 52314623 | A/T | rs1353410 | -1.59 | 0.4851 | **0.00103** | 0.22476 | 0.9975 (1b) | n | ERO1L (6.2671E-7) |
|  | 14 | 52314668 | C/T | rs140005651 | 0.78 | 2.1149 | 0.71200 | n | 0.60906 (4) | n | n |
|  | 14 | 52314795 | T/G | rs111965614 | -0.34 | 1.4389 | 0.81532 | 0.24106 | 0.60906 (4) | n | ERO1L(0.0000012) |
| *NPPA* | 1 | 11847373 | T/G | rs61757261 | -2.48 | 2.1242 | 0.24276 | 0.25150 | 0.60906 (4) | n | n |
|  | 1 | 11847392 | G/A | rs61757262 | 0.71 | 1.9207 | 0.71101 | 0.43126 | 0.51311 (3a) | n | n |
|  | 1 | 11847546 | G/A | rs5064 | -1.33 | 0.3465 | **0.00012** | 0.52453 | 0.55436 (1f) | ACBD5 (1.93E-14) | MTHFR(3.43E-263). NPPA-AS1(6.63E-148). NPPA(1.83E-44). CLCN6(1.15E-8) |
|  | 1 | 11847591 | C/T | rs5063 | 0.079 | 0.3794 | 0.83413 | 0.7573 | 0.55436 (1f) | n | MTHFR(3.27E-310). CLCN6(7.27E-310).KIAA2013(1.054E-15) |
|  | 1 | 11847592 | G/T | rs374257969 | -1.16 | 1.4234 | 0.41489 | n | 0.60906 (4) | n | n |
|  | 1 | 11848081 | C/T | rs887171068 | 0.27 | 2.1296 | 0.90080 | 0.78726 | 0.60906 (4) | n | n |
|  | 1 | 11848089 | C/G | rs41300100 | -0.29 | 1.2198 | 0.81081 | 0.04108 | 0.70497 (4) | n | n |
|  | 1 | 11848181 | T/TCAG | n | -2.64 | 2.1181 | 0.21225 | n | n | n | n |
|  | 1 | 11848241 | C/T | rs761178618 | -1.26 | 2.1221 | 0.55131 | n | 0.60906 (4) | n | n |
|  | 1 | 11846405 | C/T | rs1401425159 | -1.40 | 1.4255 | 0.32977 | 0.52671 | 0.96117 (5) | n | n |
|  | 1 | 11846412 | C/T | rs141308438 | 0.36 | 0.5003 | 0.46888 | 0.57031 | 0.38083 (1f) | SRM (0.0026). CLCN6 (0.016). FBXO2(0.053) | MTHFR(3.27E-310). CLCN6(3.27E-310). KIAA2013(2.26E-15) |
|  | 1 | 11846446 | A/C | rs769740606 | 0.13 | 2.1208 | 0.94948 | n | 0.62064 (5) | n | n |
|  | 1 | 11846537 | G/A | rs182796367 | -1.88 | 1.0429 | 0.07079 | 0.29433 | 0.18412 (7) | n | n |
|  | 1 | 11846552 | C/T | rs12744433 | 0.54 | 0.3695 | 0.14493 | 0.62878 | 0.51392 (7) | NPPA-AS1(2.17E-16). MTHFR (0.00021). MFN2 (0.041). UBIAD1 (0.044) | MFN2(2.21E-98). PLOD1(1.29E-65). NPPA(3.47E-55). NPPA-AS1(5.08E-38). MTHFR(1.61E-26). CLCN6(2.53E-20) |
|  | 1 | 11846636 | G/T | rs1231185324 | 0.18 | 0.4770 | 0.71138 | n | 0.09659 (5) | n | n |
|  | 1 | 11846725 | C/T | rs190267060 | -0.78 | 1.4334 | 0.5881 | 0.96851 | 0.13454 (5) | n | n |
|  | 1 | 11846764 | C/T | rs12744757 | 0.54 | 0.3695 | 0.14493 | 0.59453 | 0.25097 (1f) | NPPA-AS1 (2.23E-16). MTHFR (0.00021). MFN2 (0.041). UBIAD1 (0.043) | NPPA-AS1(6.37E-126). MFN2(1.17E-119). PLOD1(1.13E-84). NPPA(9.20E-42). MTHFR(9.70E-40). CLCN6(1.59E-19). KIAA2013(9.02E-10) |
|  | 1 | 11846856 | C/G | rs198368 | -1.33 | 0.3465 | **0.00012** | 0.51861 | 0.6255 (6) | NPPA-AS1 (1.46E-14) | MTHFR(4.88E-262). NPPA-AS1(4.78E-148). NPPA(2.31E-44). CLCN6(5.53E-9) |
|  | 1 | 11846867 | G/T | rs182836309 | -0.78 | 1.4334 | 0.58807 | 0.96851 | 0.16346 (6) | n | n |
|  | 1 | 11846919 | C/G | rs984766208 | 0.37 | 2.125 | 0.86315 | n | 0.33982 (5) | n | n |
|  | 1 | 11846924 | T/C | rs198370 | -1.33 | 0.3464 | **0.00012** | n | 0.55411 (5) | NPPA-AS1 (1.49E-14) | MTHFR(3.36E-263). NPPA-AS1(3.36E-145). NPPA(1.77E-44). CLCN6(1.09E-8) |
|  | 1 | 11846936 | A/C | n | 0.30 | 2.1201 | 0.88797 | n | n | n | n |
|  | 1 | 11847068 | C/G | rs371836245 | -0.68 | 0.8427 | 0.41683 | 0.04372 | 0.13454 (5) | n | n |
|  | 1 | 11846359 | G/A | rs539728220 | -0.48 | 2.1227 | 0.82246 | n | 0.58955 (5) | n | n |
|  | 1 | 11846337 | T/TTTA | n | 1.08 | 1.9086 | 0.5730 | n | n | n | n |
|  | 1 | 11846358 | C/T | rs577402137 | -0.64 | 0.6499 | 0.32848 | n | 0.58955 (5) | n | n |
| *TRIO* | 5 | 14212841 | T/G | rs140282015 | -2.41 | 1.7449 | 0.16763 | 0.36723 | 0.60906 (4) | n | n |
|  | 5 | 14212939 | T/C | rs191813208 | -2.25 | 1.7411 | 0.19595 | 0.74652 | 0.83588 (2b) | n | n |
|  | 5 | 14213114 | A/T | rs56070744 | -1.96 | 0.8378 | **0.01944** | 0.59591 | 0.48029 (3a) | n | n |
| *IRF8* | 16 | 85899296 | G/T | rs1020524794 | -3.79 | 1.3956 | **0.00666** | 0.56627 | 0.8975 (2a) | n | n |
|  | 16 | 85899785 | G/A | rs529311136 | -1.02 | 1.4814 | 0.49279 | 0.84867 | 0.60906 (4) | n | n |
| *NPPA* | 1 | 11845243 | CCTGG/C | n | -1.33 | 0.3465 | **0.00012** | 0.60745 | n | n | n |
|  | 1 | 11845457 | CCTAA/C | rs1032459143 | -0.02 | 2.1168 | 0.99360 | 0.37698 | 0.58955 (5) | n | n |
|  | 1 | 11845573 | A/T | rs1200455627 | 0.46 | 2.1182 | 0.8291 | 0.92719 | 0.3005 (5) | n | n |
|  | 1 | 11845677 | C/T | rs576810488 | -1.55 | 0.7430 | **0.03676** | 0.51623 | 0.70497 (4) | n | n |
|  | 1 | 11845740 | TCACTTTCAAAC/T | rs1645063546 | 0.54 | 0.3695 | 0.14493 | 0.59453 | n | n | n |
|  | 1 | 11845938 | C/A | rs5066 | 0.54 | 0.3695 | 0.14493 | 0.59419 | 0.035 (1b) | NPPA-AS1(2.03E-16). MTHFR (0.00021). MFN2 (0.041). UBIAD1 (0.046) | NPPA-AS1(1.98E-126).MFN2(3.21E-118).PLOD1(7.42E-84).NPPA(1.63E-42). MTHFR(1.99E-41). CLCN6(1.53E-19). KIAA2013(4.39E-10) |
| *NCAM2* | 21 | 21507970 | C/T | rs908463055 | -2.78 | 2.1206 | 0.19023 | n | 0.18412 (7) | n | n |
|  | 21 | 21508181 | A/G | rs769414668 | -1.43 | 1.0127 | 0.15654 | 0.72638 | 0.18412 (7) | n | n |
|  | 21 | 21508320 | T/G | rs970869699 | -1.78 | 2.1227 | 0.40163 | n | 0.18412 (7) | n | n |
|  | 21 | 21508385 | A/T | rs374638865 | -2.60 | 2.1150 | 0.21837 | n | 0.18412 (7) | n | n |
|  | 21 | 21508441 | A/G | rs117733668 | -0.87 | 0.4520 | 0.05536 | 0.50657 | 0.18412 (7) | n | n |
|  | 21 | 21508836 | T/TTTTTTTTTTTTTTG | n | -2.64 | 2.1160 | 0.21165 | n | n | n | n |

Abbreviations: REF. Reference allele; ALT. Alternative allele; SE. Standard error.

## **Supplementary Table S10: Evaluation of the lead loci and regions for overlap with repeat-rich or noise-prone regions**

| **Region / SNP** | **Genomic Coordinates** | **Repeat-Rich** | **Noise-Prone** | **Notes** |
| --- | --- | --- | --- | --- |
| **Single-variant association lead loci (12 assessed variants)** | | | | |
| **rs9940767** | chr16:86117778 | Yes | Yes | Located in 16p11.2. a region with known CNVs and segmental duplications |
| **rs1186847209** | chr8:102217827 | Yes | Yes | Located in 8p23.1. near defensin gene clusters and assembly gaps |
| **rs11117448** | chr16:86115575 | Yes | Yes | Same 16p11.2 region as rs9940767 |
| **rs7498190** | chr16:86115812 | Yes | Yes | Same 16p11.2 region as rs9940767 |
| **Gene aggregate test results (7 assessed genes)** | | | | |
| *AFAP1L1* gene | chr5:149.306.327–149.332.861 | Likely | Possibly | Near TERT; contains multiple enhancers and transcription factor binding sites |
| *UACA* gene | chr15:70.667.338–70.679.607 | Possibly | Possibly | Contains multiple enhancers and transcription factor binding sites; some regulatory complexity |
| **Promoter/enhancer and sliding window regions (28 assessed regions)** | | | | |
| chr7 region | chr7:148.668.001–148.672.000 | Yes | Yes | Segmental duplications |
| chr9 region | chr9:118.980.001–118.984.000 | Yes | Yes | LINE elements and tandem repeats; low mappability |
| chr5 region | chr5:16.540.456–16.540.738 | Yes | Yes | Overlaps with RepeatMasker annotations and low-complexity elements |
| chr17 region | chr17:79.841.509–79.841.692 | Yes | Yes | Located in a structurally variable region with alternate haplotypes and segmental duplications |

## **Supplementary Table S11. Enrichment analysis of A. Inherited retinal disease-causing genes, Voltage-dependent Calcium channel genes, and B. Genes with elevated expression in the retina compared to other tissues in the gene aggregate SKAT-O meta-analysis for SDR. SDR-associated genes at MAF < 0.05 and MAF < 0.01**

**A.**

| **GENE** | **PAV p-value MAF<0.05** | **PAV p-value (MAF<0.01)** | **PTV p-value MAF<0.05** | **PAV p-value (MAF<0.01)** |
| --- | --- | --- | --- | --- |
| Age-related macular degeneration (AMD) | | |  |  |
| *HTRA1* | 0.083 | 0.083 | **0.0395** | **0.0395** |
| *TLR3* | **0.006** | **0.006** | **-** | **-** |
| *HMCN1* | **0.044** | 0.124 | **-** | **-** |
| Bardet-Biedl syndrome. Autosomal recessive | | | | |
| *ARL6IP1* | **0.004** | **0.004** | 0.340 | 0.340 |
| Chorioretinal atrophy or degeneration. Autosomal dominant | | | | |
| Cone or cone-rod dystrophy. Autosomal dominant/autosomal recessive | | | | |
| *RPGRIP1* | **0.027** | 0.422 | **-** | **-** |
| Cone or cone-rod dystrophy. X-linked | | | | |
| Congenital stationary night blindness. Autosomal dominant/autosomal recessive | | | | |
| *GRK1* | **0.003** | **0.003** | **-** | **-** |
| Congenital stationary night blindness. X-linked | | | | |
| Deafness alone or syndromic. Autosomal dominant/autosomal recessive | | | | |
| Leber congenital amaurosis. Autosomal dominant/autosomal recessive | | | | |
| *RPGRIP1* | **0.027** | 0.423 | **-** | **-** |
| Macular degeneration. Autosomal dominant/autosomal recessive | | | | |
| *HMCN1* | **0.044** | 0.124 | **-** | **-** |
| Macular degeneration. X-linked | | | | |
| Ocular-retinal developmental disease. Autosomal dominant | | | | |
| Optic atrophy. Autosomal dominant/autosomal recessive | | | | |
| *OPA1* | **0.007** | 0.108 | **-** | **-** |
| Optic atrophy. X-linked | | | | |
| Other retinopathy. Autosomal dominant/autosomal recessive | | | | |
| *MAPKAPK3* | **0.011** | **0.011** | **-** | **-** |
| *LRP5* | **0.008** | 0.105 | **-** | **-** |
| *CYP4V2* | 0.893 | 0.389 | **0.044** | **0.044** |
| Other retinopathy. Mitochondrial | | | | |
| Other retinopathy. X-linked | | | | |
| Retinitis pigmentosa. Autosomal dominant/autosomal recessive | | | | |
| *ZNF513* | **0.044** | **0.044** | **-** | **-** |
| *USH2A* | 0.259 | **0.050** | 0.520 | 0.520 |
| *CYP4V2* | 0.893 | 0.389 | **0.044** | **0.044** |
| Retinitis pigmentosa. X-linked | | | | |
| Retinopathy of prematurity (ROP) | | | | |
| Syndromic/systemic diseases with retinopathy. Autosomal dominant/autosomal recessive | | | | |
| *TUBGCP4* | **0.014** | **0.014** | **-** | **-** |
| *LRP5* | **0.008** | 0.105 | **-** | **-** |
| Syndromic/systemic diseases with retinopathy. X-linked | | | | |
| Usher syndrome. Autosomal recessive | | | | |
| *USH2A* | 0.259 | **0.050** | 0.520 | 0.520 |
| Voltage-dependent Calcium channel genes | | | | |
| *CACNG7* | **0.006** | **0.006** | **-** | **-** |
| *CACNB3* | **0.021** | 0.326 | **-** | **-** |
| *CACNA1C* | **0.008** | **0.0008** | **-** | **-** |
| *CACNA1I* | 0.494 | **0.005** | **-** | **-** |

Abbreviations: PAV. Protein Altering Variants; PTV. Protein Truncating Variants; MAF. Minor Allele Frequency.

Nominally (P<0.05) significant genes were highlighted among all the tested genes in different categories.

**B.**

| **GENE** | **PAV p-value MAF<0.05** | **PAV p-value (MAF<0.01)** | **PTV p-value MAF<0.05** | **PAV p-value (MAF<0.01)** |
| --- | --- | --- | --- | --- |
| Genes with elevated expression in the retina compared to other tissues | | | | |
| *GRK1* | **0.003** | **0.003** | n | n |
| *TNRC6C* | **0.006** | 0.614 | 0.325 | 0.328 |
| *ZDHHC2* | **0.009** | **0.009** | n | n |
| *ZFR2* | **0.01** | 0.060 | n | n |
| *LRTM1* | **0.011** | 0.140 | **0.010** | n |
| *TRPC3* | **0.012** | **0.012** | n | n |
| *MSI1* | **0.013** | **0.013** | n | n |
| *FOXH1* | **0.014** | **0.014** | n | n |
| *MAP6* | **0.014** | 0.737 | n | n |
| *GABRB3* | **0.015** | **0.015** | n | n |
| *GPR61* | **0.018** | **0.018** | n | n |
| *BHLHE41* | **0.020** | **0.020** | n | n |
| *KIF1B* | **0.020** | 0.866 | n | n |
| *ZNF625-ZNF20* | **0.021** | **0.010** | n | n |
| *FAIM* | **0.024** | n | n | n |
| *GDPD4* | **0.025** | 0.625 | n | n |
| *GSC2* | **0.026** | **0.026** | n | n |
| *RPGRIP1* | **0.027** | 0.423 | n | n |
| *FAM169A* | **0.028** | **0.021** | n | n |
| *C16orf92* | **0.029** | **0.029** | n | n |
| *C16orf74* | **0.037** | **0.037** | n | n |
| *SCG3* | **0.042** | 0.607 | n | n |
| *NT5E* | **0.045** | **0.045** | n | n |
| *GNB1* | **0.046** | **0.046** | n | n |
| *EPB41* | **0.047** | 0.382 | n | n |
| *RALGPS2* | **0.048** | **0.048** | n | n |
| *MGARP* | **0.050** | **0.050** | n | n |
| *OR10AD1* | 0.387 | **0.012** | n | n |
| *CCSAP* | 0.348 | **0.020** | n | n |
| *SLC1A7* | 0.522 | **0.023** | n | n |
| *DNAH17* | 0.350 | **0.027** | 1 | 1 |
| *SYDE2* | 0.392 | **0.030** | 0.613 | **0.023** |
| *NTM* | 0.069 | **0.030** | n | n |
| *PLA2G4C* | 0.068 | **0.041** | 0.803 | n |
| *MSI2* | 0.058 | **0.047** | n | n |
| *USH2A* | 0.259 | **0.050** | 0.520 | 0.520 |
| *KCNAB3* | 0.268 | 0.268 | **0.036** | **0.036** |

Abbreviations: PAV. Protein Altering Variants; PTV. Protein Truncating Variants; MAF. Minor Allele Frequency.

Nominally (P<0.05) significant genes were highlighted among all the tested genes in different categories.

## **Supplementary Table S12. Significantly enriched pathways (false discovery rate, FDR < 0.05) from the PAN-GO PANTHER Overrepresentation Test for the 12 promoter analysis lead genes**

| **PAN-GO biological process** | **N genes in the pathway** | **N genes associated with SDR** | **Expected** | **Enrichment direction** | **Fold Enrichment** | **Raw P-value** | **FDR-adjusted P-value** |
| --- | --- | --- | --- | --- | --- | --- | --- |
| cellular response to prostaglandin E stimulus (GO:0071380) | 2 | 2 | .00 | + | > 100 | 3.59E-07 | 2.79E-03 |
| cellular response to prostaglandin stimulus (GO:0071379) | 2 | 2 | .00 | + | > 100 | 3.59E-07 | 1.40E-03 |
| response to prostaglandin E (GO:0034695) | 2 | 2 | .00 | + | > 100 | 3.59E-07 | 9.31E-04 |
| response to prostaglandin (GO:0034694) | 2 | 2 | .00 | + | > 100 | 3.59E-07 | 6.99E-04 |
| cellular response to alcohol (GO:0097306) | 2 | 2 | .00 | + | > 100 | 3.59E-07 | 5.59E-04 |
| cellular response to ketone (GO:1901655) | 4 | 2 | .00 | + | > 100 | 2.15E-06 | 2.79E-03 |
| response to alcohol (GO:0097305) | 6 | 2 | .00 | + | > 100 | 5.37E-06 | 5.98E-03 |
| response to ketone (GO:1901654) | 7 | 2 | .00 | + | > 100 | 7.52E-06 | 7.32E-03 |

## **Supplementary Table S13. g:Profiler Enrichment Summary**

| **Input Genes/Regions** | **Analysis type** | **Key Findings** | **Query genes contributing to enrichment** | **FDR-adjusted P-value** |
| --- | --- | --- | --- | --- |
| Lead genes from promoter analysis | KEGG pathway enrichment | Renin secretion (KEGG:04924) – vascular regulation and endothelial function | *PTGER2. NPPA* | 0.0469 |
| Closest genes to sliding window regions | miRNA enrichment | hsa-miR-494 (FDR = 9.87×10⁻³); hsa-miR-603 (FDR = 1.14×10⁻²) – post-transcriptional regulation | *CUL1. SMCHD1. DOK5. H6PD. MCPH1. PCDH10; CSMD2. CUL1. SMCHD1. MCPH1. PCDH1. ADCYAP1* | 0.0099;  0.0114 |
| Closest genes to sliding window regions | CORUM complex enrichment | Ubiquitin E3 ligase complex (CHEK1, CUL1) – protein degradation and cell cycle regulation | *CUL1* | 0.0499 |

## **Supplementary Table S14: FinnDiane Study Centers**

| **FinnDiane Study Centers** | **Physicians and nurses** |
| --- | --- |
| **Anjalankoski Health Center** | S.Koivula. T.Uggeldahl |
| **Central Finland Central Hospital. Jyväskylä** | T.Forslund. A.Halonen. A.Koistinen. P.Koskiaho. M.Laukkanen. J.Saltevo. M.Tiihonen |
| **Central Hospital of Åland Islands. Mariehamn** | M.Forsen. H.Granlund. A.-C.Jonsson. B.Nyroos |
| **Central Hospital of Kanta-Häme. Hämeenlinna** | P.Kinnunen. A.Orvola. T.Salonen. A.Vähänen |
| **Central Hospital of Kymenlaakso. Kotka** | R.Paldanius. M.Riihelä. L.Ryysy |
| **Central Hospital of Länsi-Pohja. Kemi** | H.Laukkanen. P.Nyländen. A.Sademies |
| **Central Ostrobothnian Hospital District. Kokkola** | S.Anderson. B.Asplund. U.Byskata. P.Liedes. M.Kuusela. T.Virkkala |
| **City of Espoo Health Center:** |  |
| **Espoonlahti** | A.Nikkola. E.Ritola |
| **Tapiola** | M.Niska. H.Saarinen |
| **Samaria** | E.Oukko-Ruponen. T.Virtanen |
| **Viherlaakso** | A.Lyytinen |
| **City of Helsinki Health Center:** |  |
| **Puistola** | H.Kari. T.Simonen |
| **Suutarila** | A.Kaprio. J.Kärkkäinen. B.Rantaeskola |
| **Töölö** | P.Kääriäinen. J.Haaga. A-L.Pietiläinen |
| **City of Hyvinkää Health Center** | S.Klemetti. T.Nyandoto. E.Rontu. S.Satuli-Autere |
| **City of Vantaa Health Center:** |  |
| **Korso** | R.Toivonen. H.Virtanen |
| **Länsimäki** | R.Ahonen. M.Ivaska-Suomela. A.Jauhiainen |
| **Martinlaakso** | M.Laine. T.Pellonpää. R.Puranen |
| **Myyrmäki** | A.Airas. J.Laakso. K.Rautavaara |
| **Rekola** | M.Erola. E.Jatkola |
| **Tikkurila** | R.Lönnblad. A.Malm. J.Mäkelä. E.Rautamo |
| **Heinola Health Center** | P.Hentunen. J.Lagerstam |
| **Helsinki University Hospital. Department of Medicine. Division of Nephrology** | T.Claesson. A.Dufva. N.Elonen. M.Eriksson. J.Fagerudd. M.Feodoroff. D.Gordin. P.-H.Groop. O.Heikkilä. K.Hietala. S.Hägg-Holmberg. F.Jansson Sigfrids. M.Korolainen. J.Kytö. S.Lindh. H.Paajanen. K.Pettersson-Fernholm. K.Rimpeläinen. M.Rosengård-Bärlund. M.Rönnback. L.Salovaara. A.Sandelin. M.Saraheimo. S.Satuli-Autere. R.Simonsen. P.Smidtslund. L.Thorn. H.Tikkanen. J.Tuomikangas. A.Tynjälä. K.Uljala. T.Vesisenaho. J.Wadén. A.Ylinen |
| **Herttoniemi Hospital. Helsinki** | V.Sipilä |
| **Hospital of Lounais-Häme. Forssa** | T.Kalliomäki. J.Koskelainen. R.Nikkanen. N.Savolainen. H.Sulonen. E.Valtonen |
| **Hyvinkää Hospital** | L. Norvio. A.Hämäläinen |
| **Iisalmi Hospital** | E.Toivanen |
| **Jokilaakso Hospital. Jämsä** | A.Parta. I.Pirttiniemi |
| **Jorvi Hospital. Helsinki University Central Hospital** | S.Aranko. S.Ervasti. R.Kauppinen-Mäkelin. A.Kuusisto. T.Leppälä. K.Nikkilä. L.Pekkonen |
| **Jyväskylä Health Center. Kyllö** | K.Nuorva. M.Tiihonen |
| **Kainuu Central Hospital. Kajaani** | S.Jokelainen. K.Kananen. M.Karjalainen. P.Kemppainen. A-M.Mankinen. A.Reponen. M.Sankari |
| **Kerava Health Center** | H.Stuckey. P.Suominen |
| **Kirkkonummi Health Center** | A.Lappalainen. M.Liimatainen. J.Santaholma |
| **Kivelä Hospital. Helsinki** | A.Aimolahti. E.Huovinen |
| **Koskela Hospital. Helsinki** | V.Ilkka. M.Lehtimäki |
| **Kotka Health Center** | E.Pälikkö-Kontinen. A.Vanhanen |
| **Kouvola Health Center** | E.Koskinen. T.Siitonen |
| **Kuopio University Hospital** | E.Huttunen. R.Ikäheimo. P.Karhapää. P.Kekäläinen. M.Laakso. T.Lakka. E.Lampainen. L.Moilanen. S. Tanskanen. L.Niskanen. U.Tuovinen. I.Vauhkonen. E.Voutilainen |
| **Kuusamo Health Center** | T.Kääriäinen. E.Isopoussu |
| **Kuusankoski Hospital** | E.Kilkki. I.Koskinen. L.Riihelä |
| **Laakso Hospital. Helsinki** | T.Meriläinen. P.Poukka. R.Savolainen. N.Uhlenius |
| **Lahti City Hospital** | A.Mäkelä. M.Tanner |
| **Lapland Central Hospital. Rovaniemi** | L.Hyvärinen. K.Lampela. S.Pöykkö. T.Rompasaari. S.Severinkangas. T.Tulokas |
| **Lappeenranta Health Center** | P. Erola. L.Härkönen. P.Linkola. T.Pekkanen. I.Pulli. E.Repo |
| **Lohja Hospital** | T.Granlund. K.Hietanen. M.Porrassalmi. M.Saari. T.Salonen. M.Tiikkainen. |
| **Länsi-Uusimaa Hospital. Tammisaari** | I.-M.Jousmaa. J.Rinne |
| **Loimaa Health Center** | A.Mäkelä. P.Eloranta |
| **Malmi Hospital. Helsinki** | H.Lanki. S.Moilanen. M.Tilly-Kiesi |
| **Mikkeli Central Hospital** | A.Gynther. R.Manninen. P.Nironen. M.Salminen. T.Vänttinen |
| **Mänttä Regional Hospital** | I.Pirttiniemi. A-M.Hänninen |
| **North Karelian Hospital. Joensuu** | U-M.Henttula. P.Kekäläinen. M.Pietarinen. A.Rissanen. M.Voutilainen |
| **Nurmijärvi Health Center** | A.Burgos. K.Urtamo |
| **Oulaskangas Hospital. Oulainen** | E.Jokelainen. P-L.Jylkkä. E.Kaarlela. J.Vuolaspuro |
| **Oulu Health Center** | L.Hiltunen. R.Häkkinen. S.Keinänen-Kiukaanniemi |
| **Oulu University Hospital** | R.Ikäheimo |
| **Päijät-Häme Central Hospital** | H.Haapamäki. A.Helanterä. S.Hämäläinen. V.Ilvesmäki. H.Miettinen |
| **Palokka Health Center** | P.Sopanen. L.Welling |
| **Pieksämäki Hospital** | V.Sevtsenko. M.Tamminen |
| **Pietarsaari Hospital** | M-L.Holmbäck. B.Isomaa. L.Sarelin |
| **Pori City Hospital** | P.Ahonen. P.Merisalo. E.Muurinen. K.Sävelä |
| **Porvoo Hospital** | M.Kallio. B.Rask. S.Rämö |
| **Raahe Hospital** | A.Holma. M.Honkala. A.Tuomivaara. R.Vainionpää |
| **Rauma Hospital** | K.Laine. K.Saarinen. T.Salminen |
| **Riihimäki Hospital** | P.Aalto. E.Immonen. L.Juurinen |
| **Salo Hospital** | A.Alanko. J.Lapinleimu. P.Rautio. M.Virtanen |
| **Satakunta Central Hospital. Pori** | M.Asola. M.Juhola. P.Kunelius. M.-L.Lahdenmäki. P.Pääkkönen. M.Rautavirta |
| **Savonlinna Central Hospital** | T.Pulli. P.Sallinen. M.Taskinen. E.Tolvanen. T.Tuominen. H.Valtonen. A.Vartia. S-L.Viitanen |
| **Seinäjoki Central Hospital** | O.Antila. E.Korpi-Hyövälti. T.Latvala. E.Leijala. T.Leikkari. M.Punkari N.Rantamäki. H.Vähävuori |
| **South Karelia Central Hospital. Lappeenranta** | T.Ensala. E.Hussi. R.Härkönen. U.Nyholm. J.Toivanen |
| **Tampere Health Center** | A.Vaden. P.Alarotu. E.Kujansuu. H.Kirkkopelto-Jokinen. M.Helin. S.Gummerus. L.Calonius. T.Niskanen. T.Kaitala. T.Vatanen |
| **Tampere University Hospital** | P. Hannula. I.Ala-Houhala. R.Kannisto. T.Kuningas. P.Lampinen. M.Määttä.H.Oksala. T.Oksanen. A.Putila. H.Saha. K.Salonen. H.Tauriainen. S.Tulokas |
| **Tiirismaa Health Center. Hollola** | T.Kivelä. L.Petlin. L.Savolainen |
| **Turku Health Center** | A.Artukka. I.Hämäläinen. L.Lehtinen. E.Pyysalo. H.Virtamo. M.Viinikkala. M.Vähätalo |
| **Turku University Central Hospital** | K.Breitholz. R.Eskola. K.Metsärinne. U.Pietilä. P.Saarinen. R.Tuominen. S.Äyräpää |
| **Vaajakoski Health Center** | K.Mäkinen. P.Sopanen |
| **Valkeakoski Regional Hospital** | S.Ojanen. E.Valtonen. H.Ylönen. M.Rautiainen. T.Immonen |
| **Vammala Regional Hospital** | I.Isomäki. R.Kroneld. L.Mustaniemi. M.Tapiolinna-Mäkelä |
| **Vasa Central Hospital** | S.Bergkulla. U.Hautamäki. V-A.Myllyniemi. I.Rusk |

**Supplementary Figure S1. Statistical power to detect suggestive associations (P < 1 ×10^-5^) with SDR**

Power calculated for MAF of 0.5%. 1%. 2%. 3%. 4%. 5%. 10%. and 20%. Calculated with the R genpwr package. α=1×10^-5^. N cases=795. N controls =276. N total=1071.


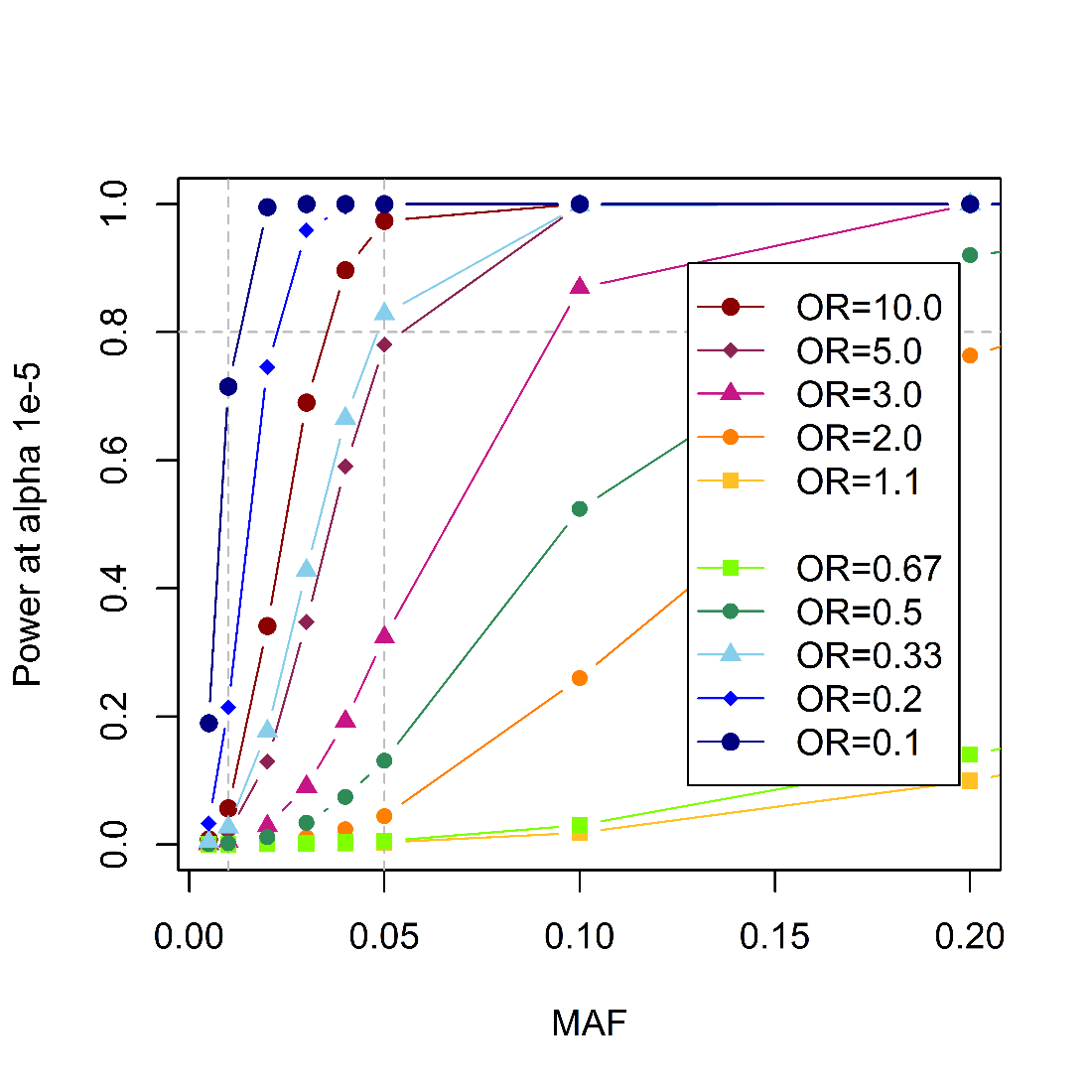


## **Supplementary Figure S2. Manhattan plot of single variant results**


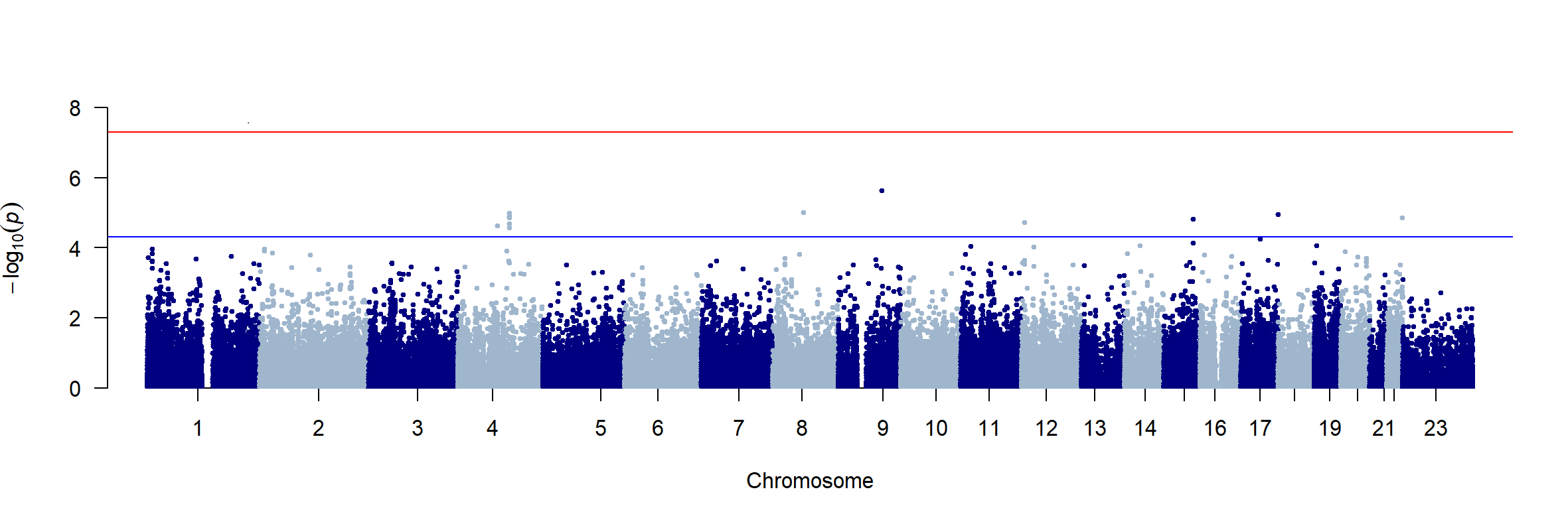


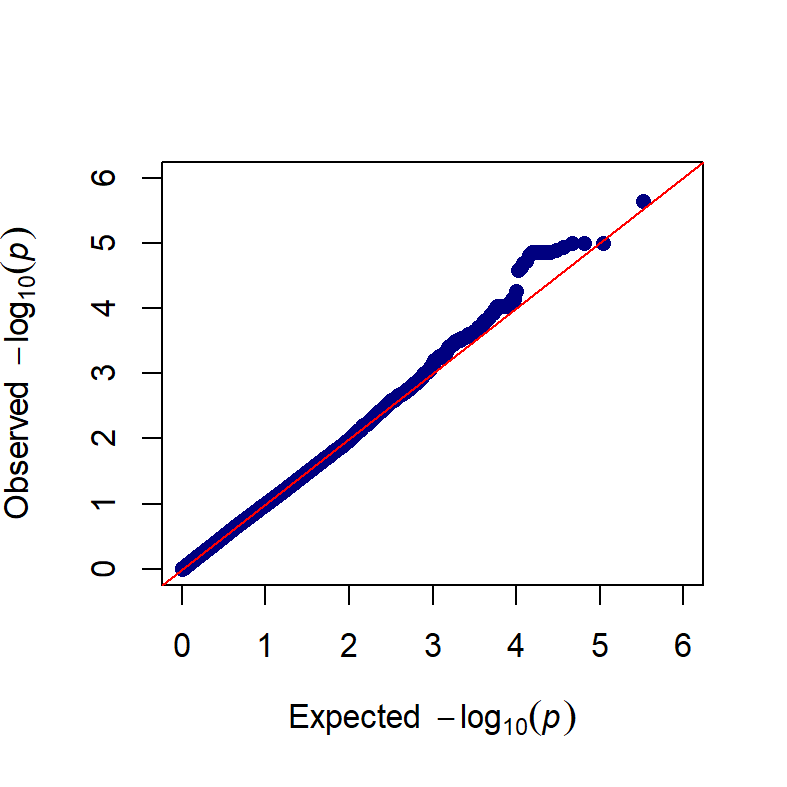


**Supplementary Figure S3. Manhattan plot for sliding window-based tests (STAAR)**

1:33550001-33554000 (***CSMD2)***


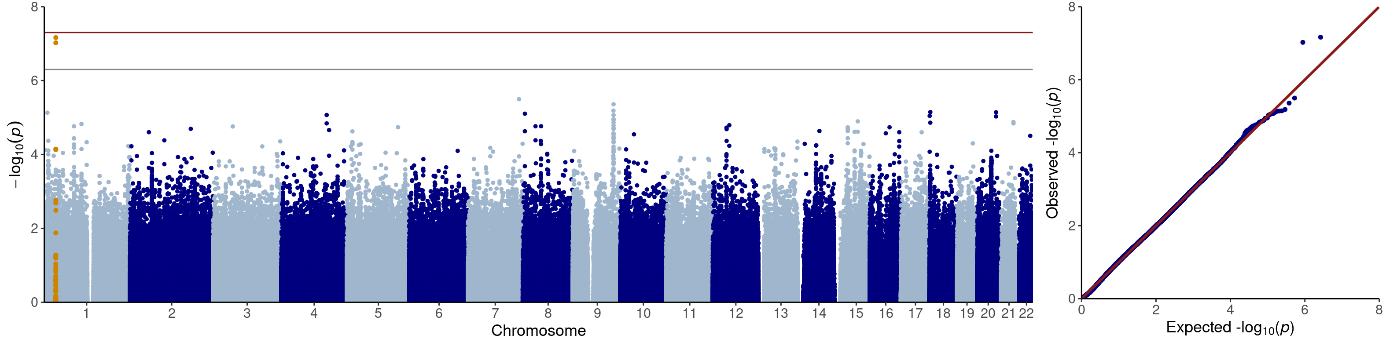


**Supplementary Figure S4. Manhattan plot for enhancer regions with low-frequency variants (MAF < 5%) (STAAR)**

Significance thresholds were calculated according to the number of conducted tests; for SDR, the threshold was 2×10^-6^.


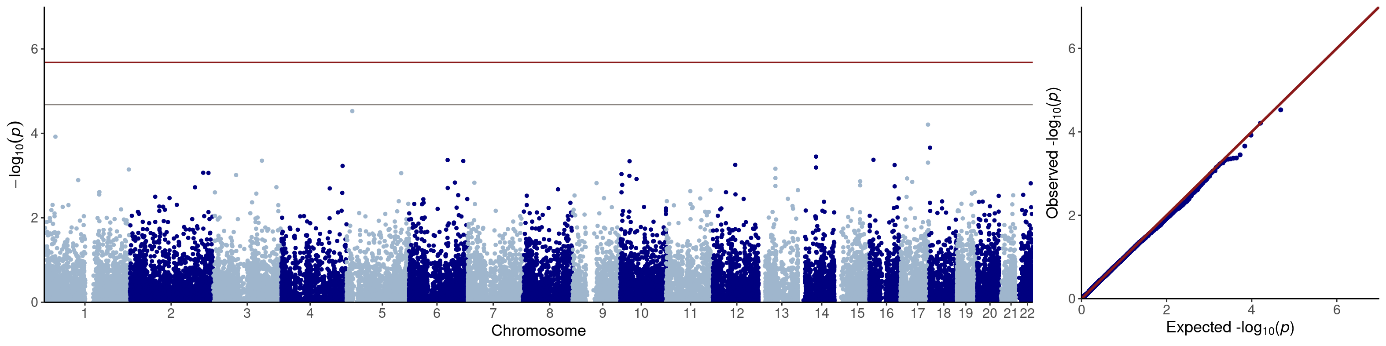


**Supplementary Figure S5. Manhattan plot for promoter regions with low-frequency variants (MAF < 5%) (STAAR)**

Significance thresholds were calculated according to the number of conducted tests; for SDR the threshold was 3×10^-7^.


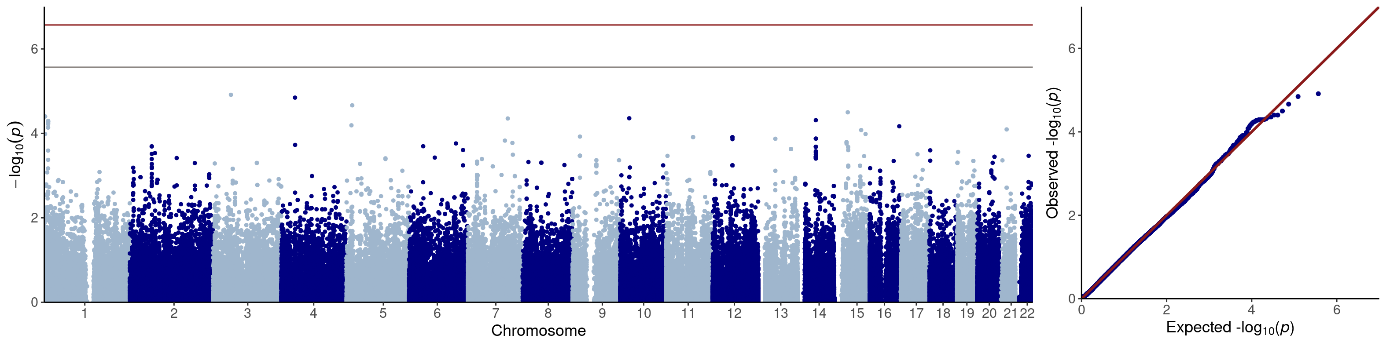


## **Supplementary Figure S6. STRING network analysis**


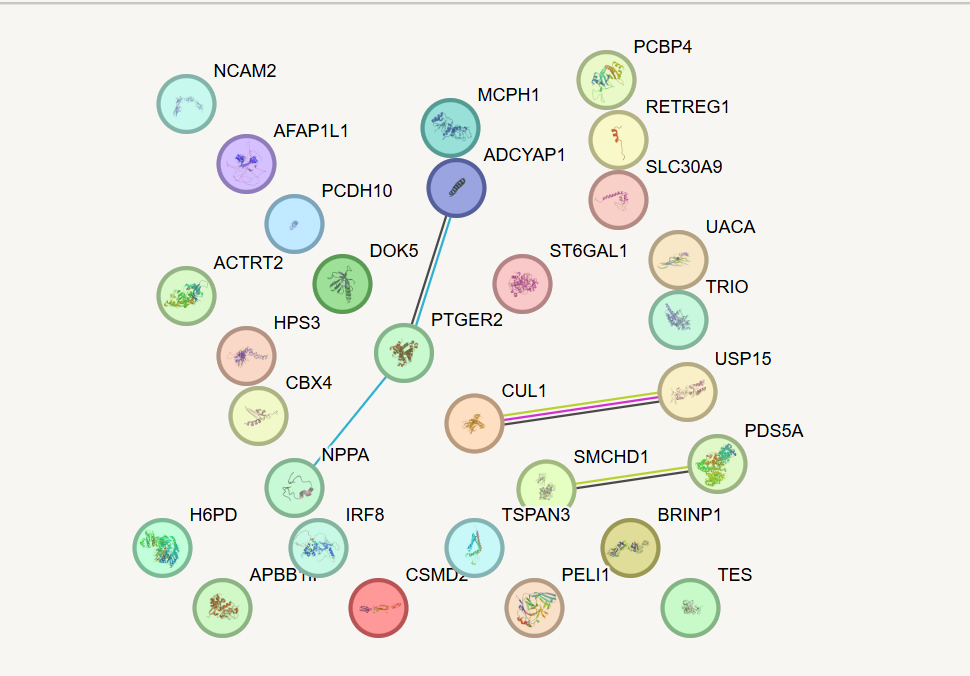


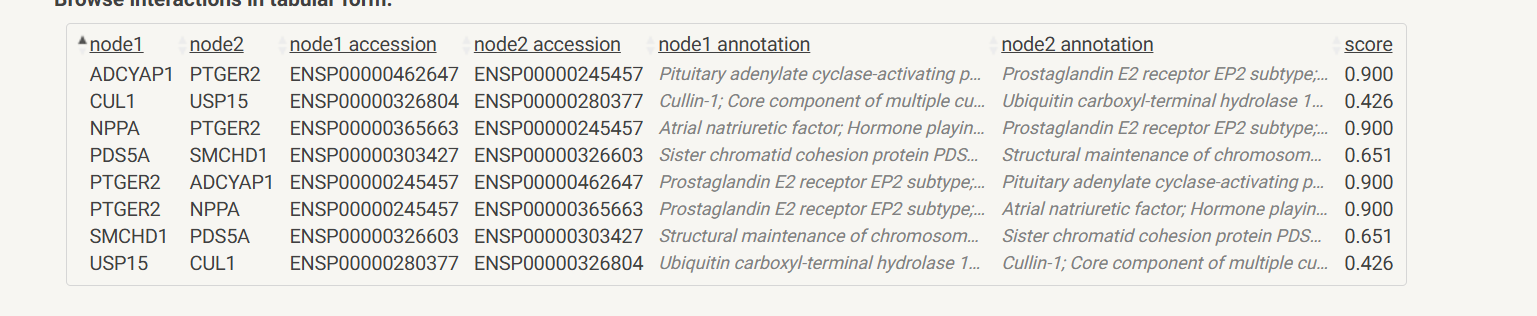


## **Supplementary Figure S7. Single-cell tissue expression for genes associated with SDR: *IRF8* (A), *ZNF367* (B), *AFAP1L1* (C), *SLC30A9* (D), *HPS3* (E), *PELI1* (F), *CSMD2* (G), and *PCBP4* (H)**

**A.** Eye: *IRF8*

**
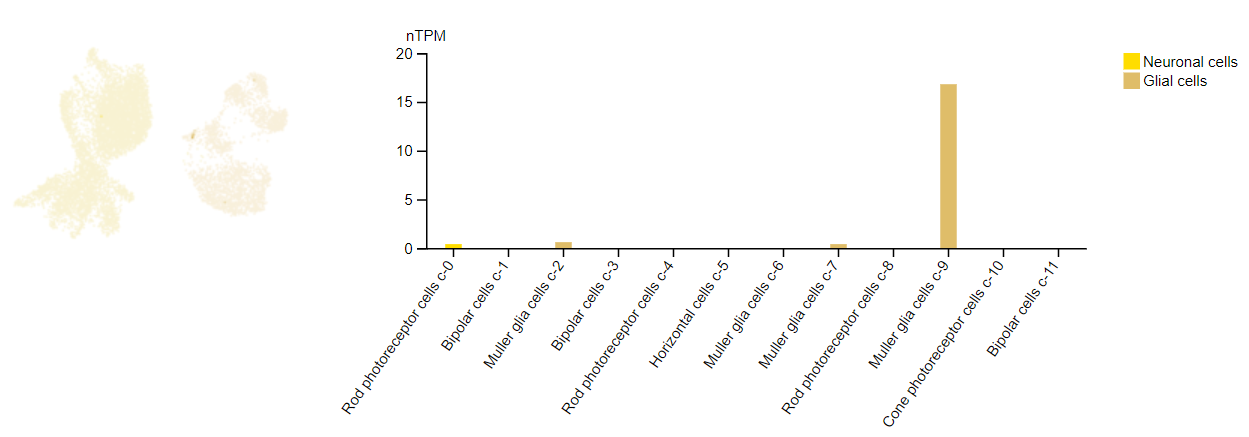
**

**
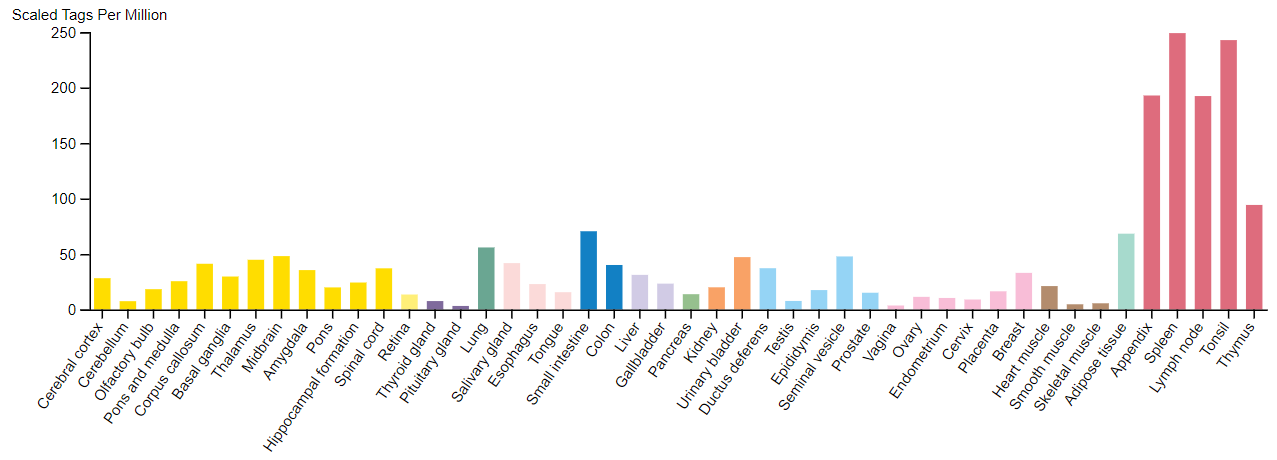
**

*IRF8* expression overview based on FANTOM5 CAGE data

**B.** Eye: *ZNF367*


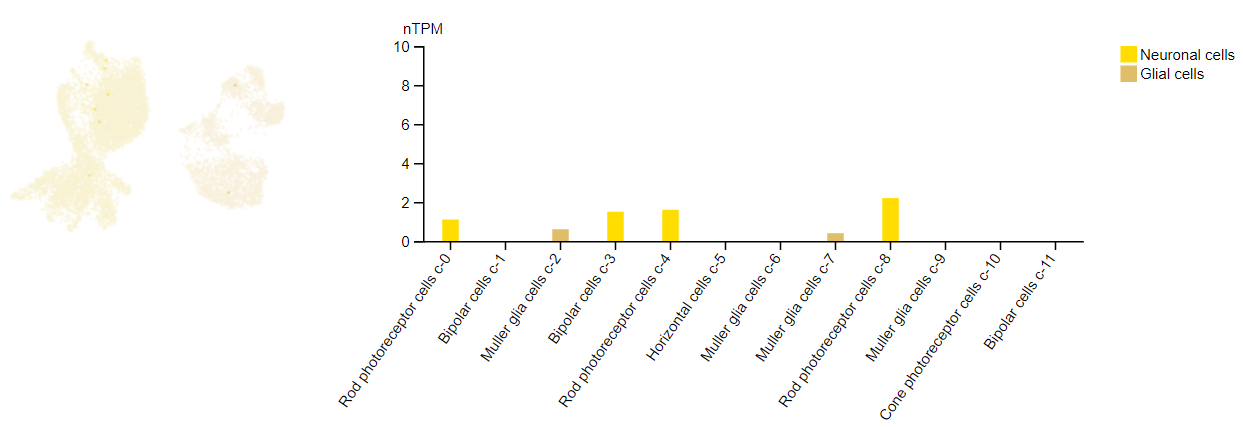


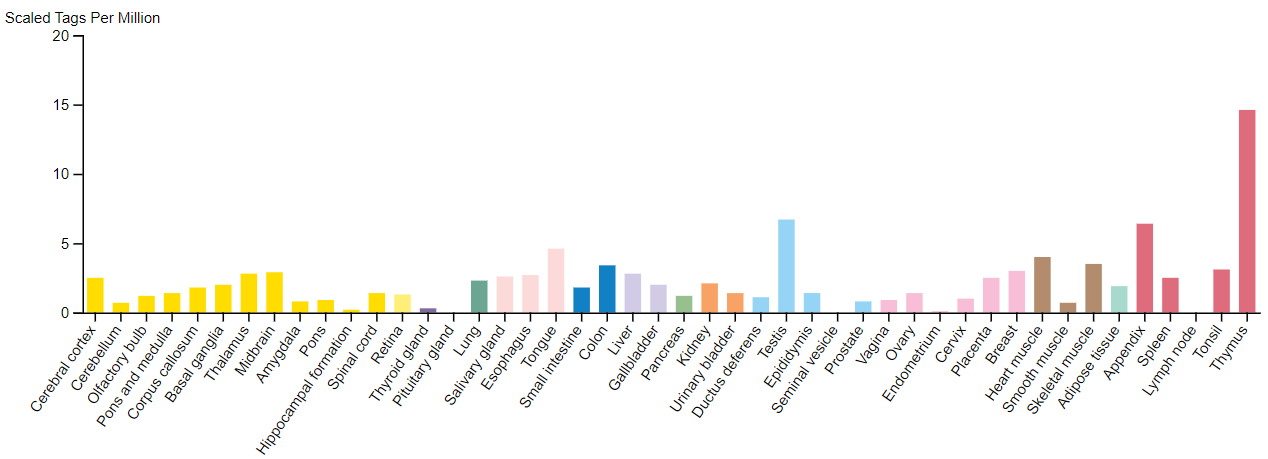


*ZNF367* expression overview based on FANTOM5 CAGE data

**C.** Eye: *AFAP1L1*


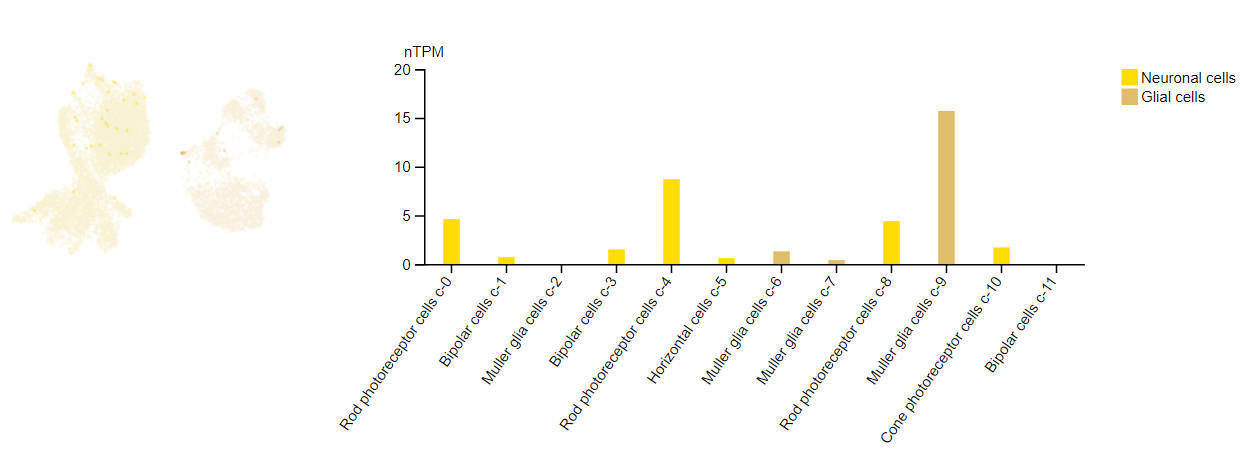


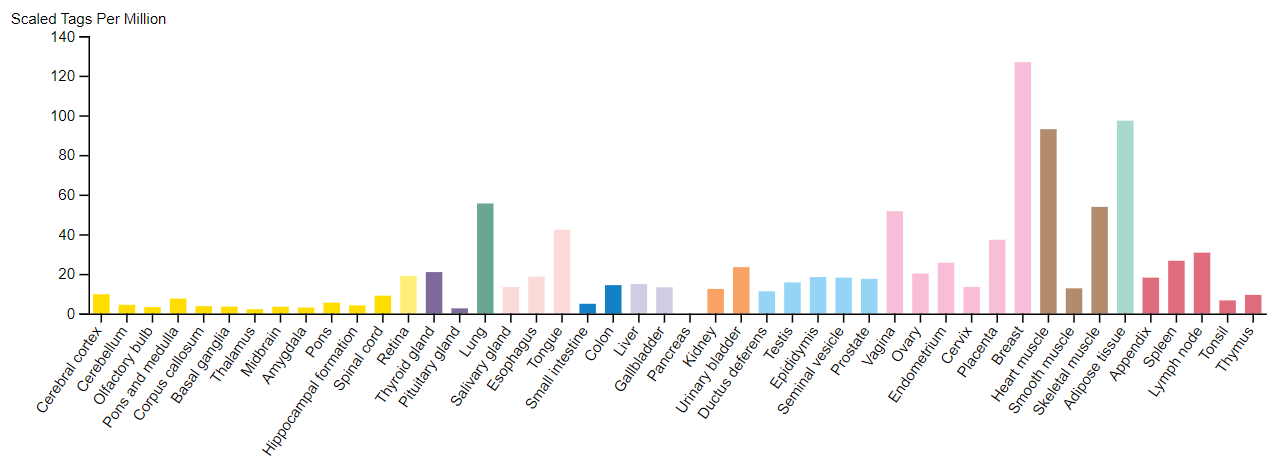


*AFAP1L1* expression overview based on FANTOM5 CAGE data

**D.** Eye: *SLC30A9*


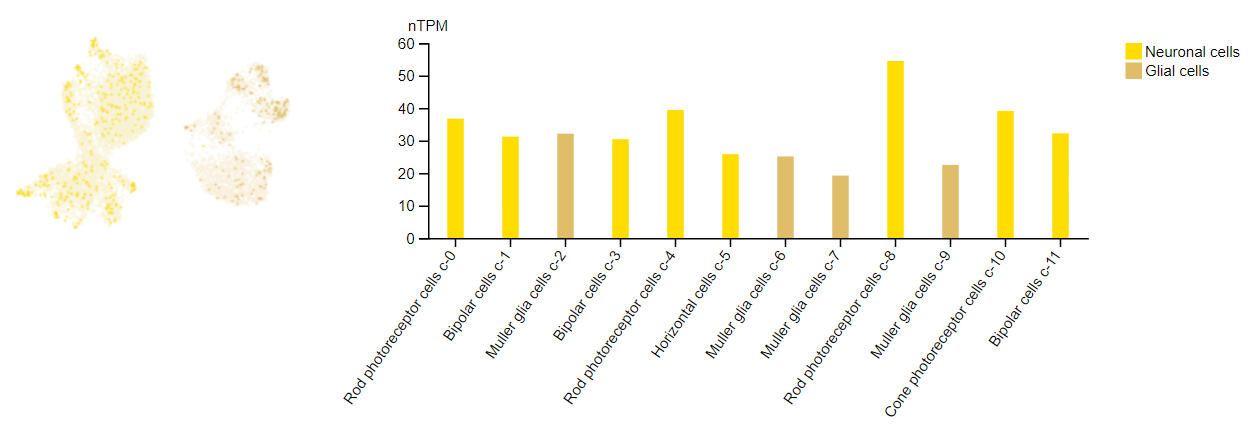


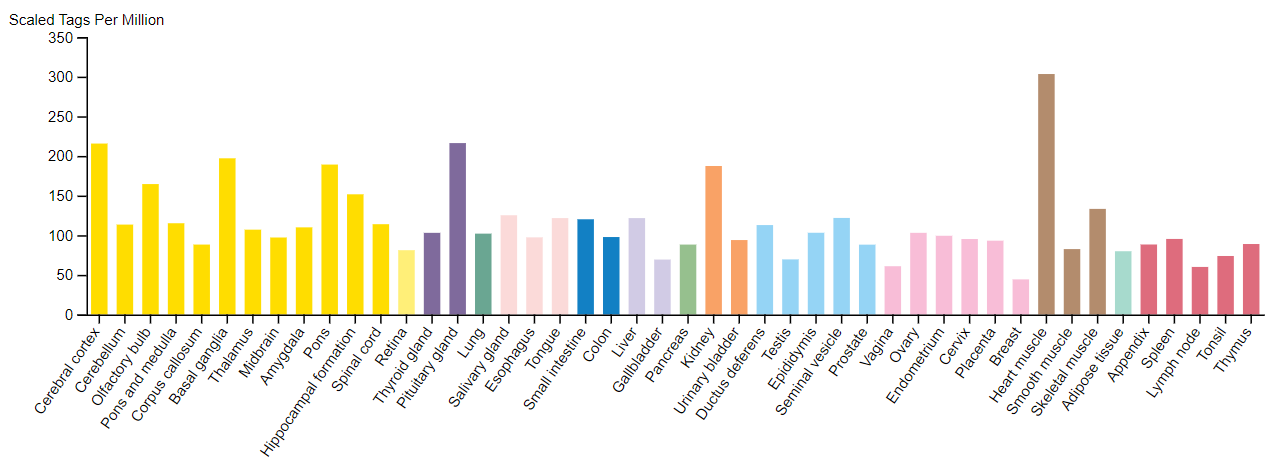


*SLC30A9* expression overview based on FANTOM5 CAGE data

**E.** Eye: *HPS3*


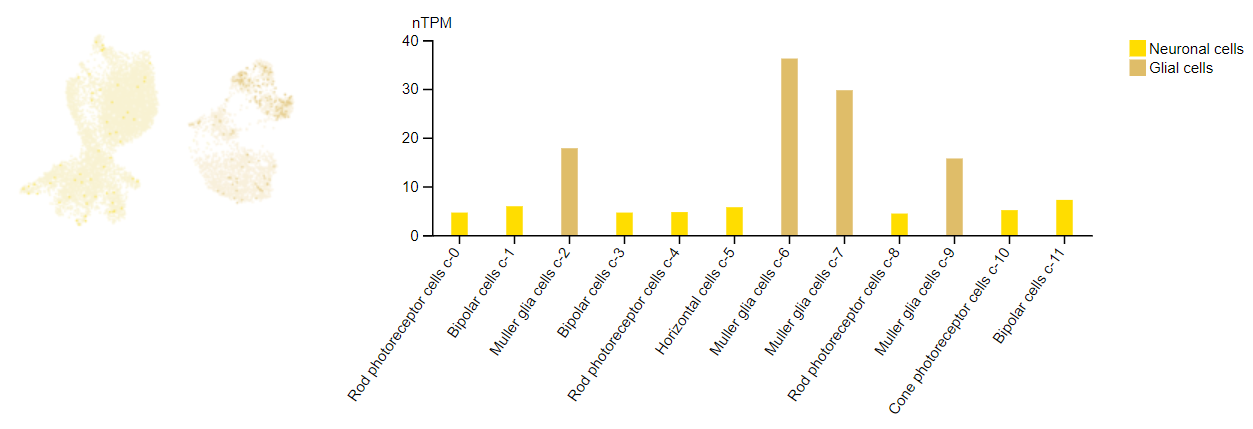


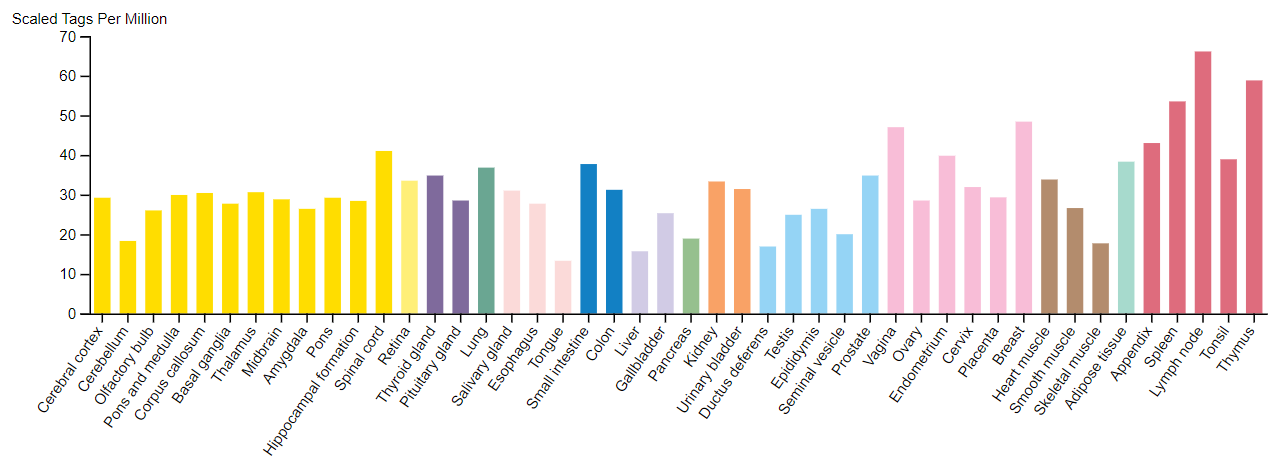


*HPS3* expression overview based on FANTOM5 CAGE data

**F.** Eye: *PELI1*


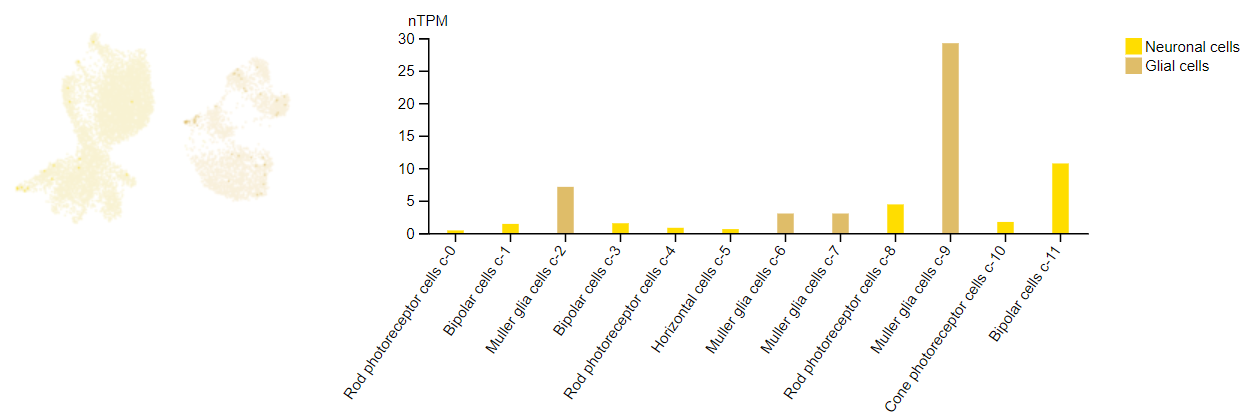


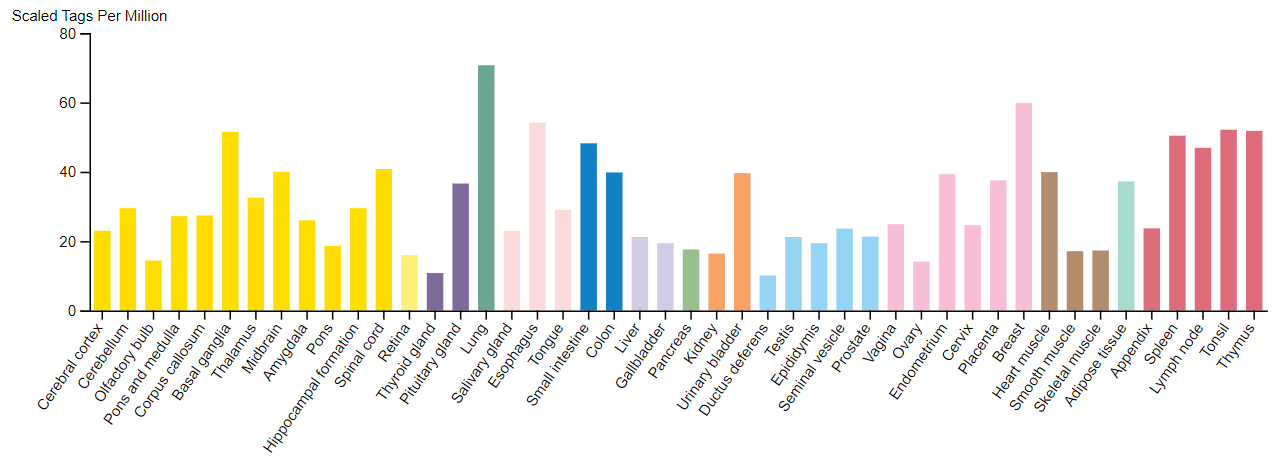


*PELI1* expression overview based on FANTOM5 CAGE data

**G.** Eye: *CSMD2*


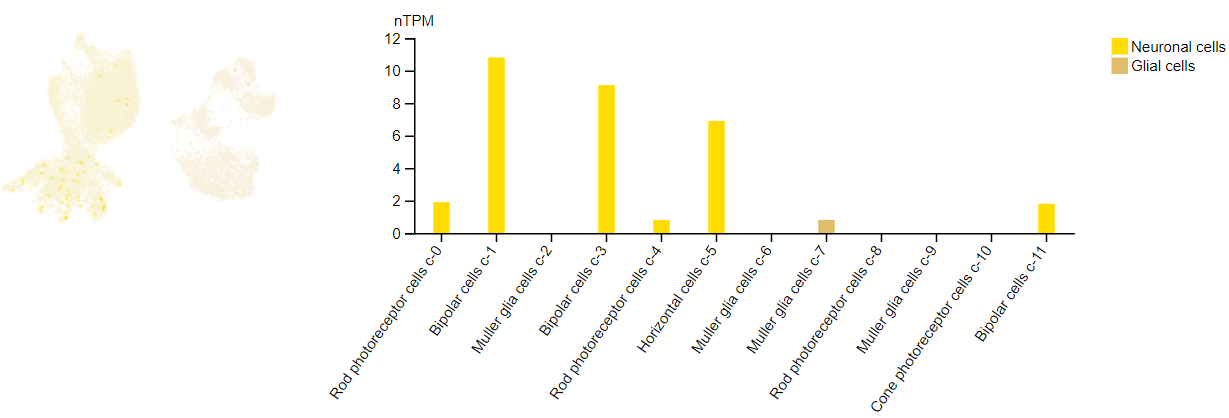


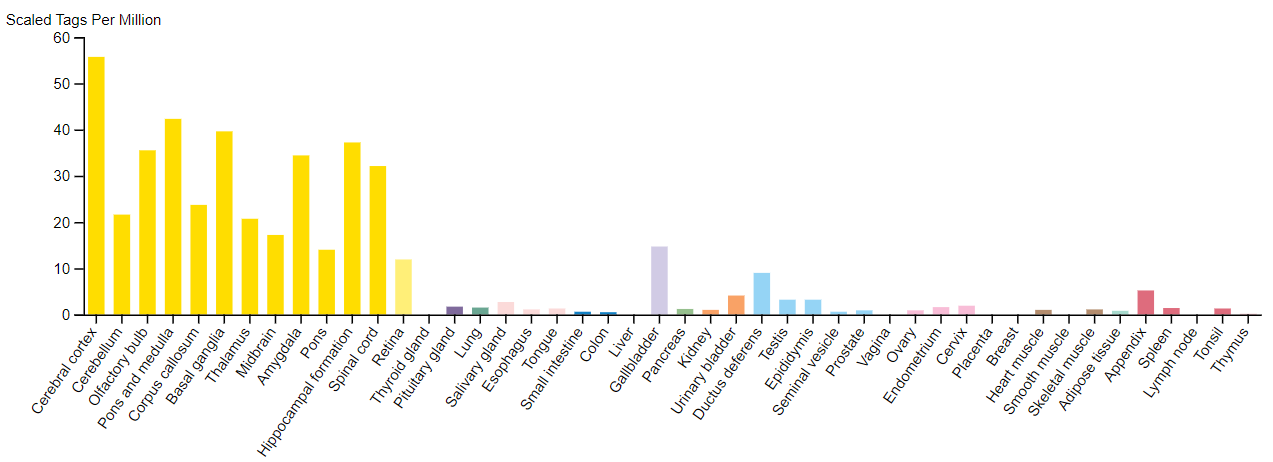


*CSMD2* expression overview based on FANTOM5 CAGE data

**H.** Eye: *PCBP4*


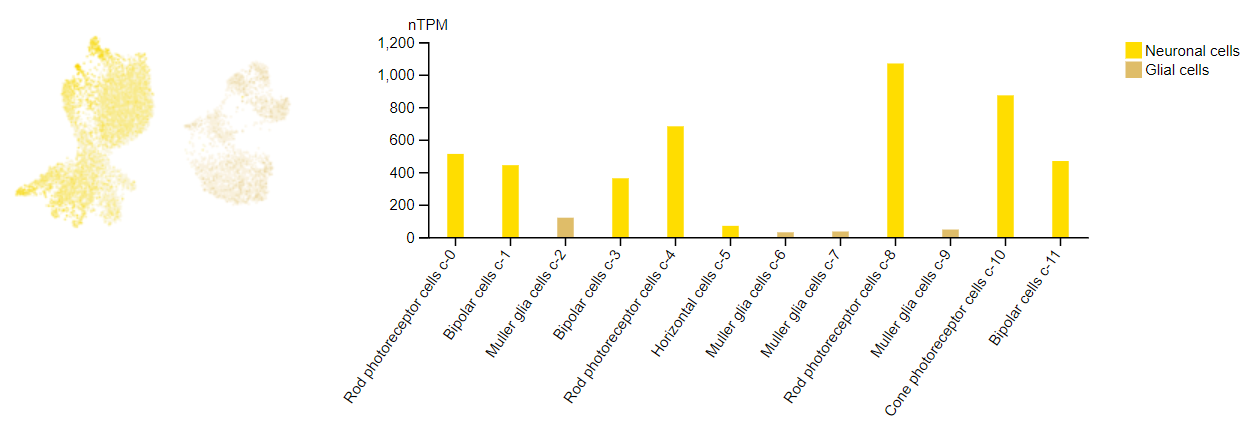


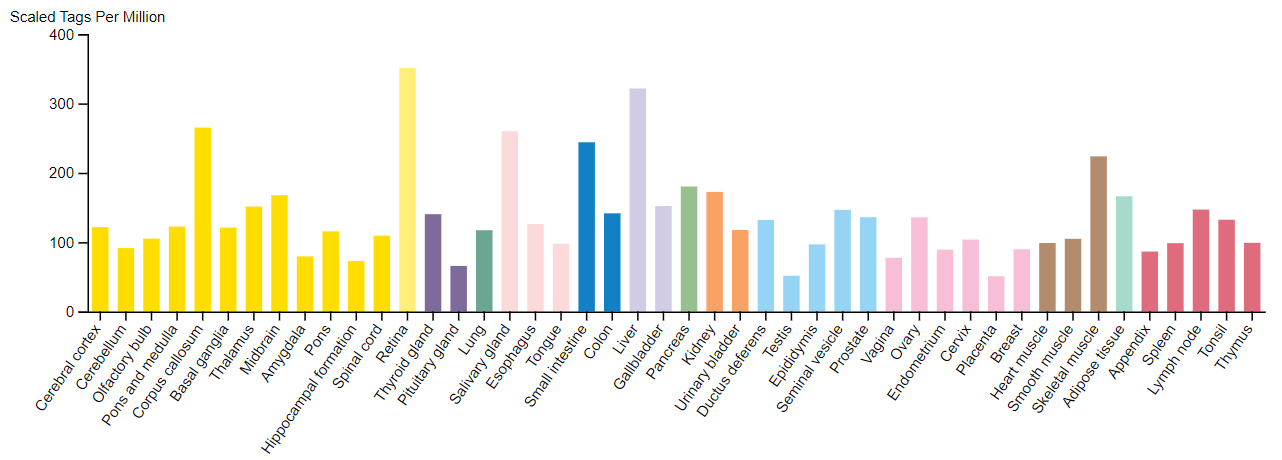


*PCBP4* expression overview based on FANTOM5 CAGE data

## **References**

1. Haukka JK. Antikainen AA. Valo E. et al. Whole-exome and whole-genome sequencing of 1064 individuals with type 1 diabetes reveals novel genes for diabetic kidney disease. *Diabetologia*. Published online August 6. 2024. doi:10.1007/s00125-024-06241-1

2. Sandholm N. Van Zuydam N. Ahlqvist E. et al. The Genetic Landscape of Renal Complications in Type 1 Diabetes. *J Am Soc Nephrol*. 2017;28(2):557-574. doi:10.1681/ASN.2016020231

3. Zhan X. Hu Y. Li B. Abecasis GR. Liu DJ. RVTESTS: an efficient and comprehensive tool for rare variant association analysis using sequence data. *Bioinformatics*. 2016;32(9):1423-1426.

4. Willer CJ. Li Y. Abecasis GR. METAL: fast and efficient meta-analysis of genomewide association scans. *Bioinformatics*. 2010;26(17):2190-2191. doi:10.1093/bioinformatics/btq340

5. Lee S. Teslovich TM. Boehnke M. Lin X. General framework for meta-analysis of rare variants in sequencing association studies. *Am J Hum Genet*. 2013;93(1):42-53. doi:10.1016/j.ajhg.2013.05.010

6. Li X. Li Z. Zhou H. et al. Dynamic incorporation of multiple in silico functional annotations empowers rare variant association analysis of large whole-genome sequencing studies at scale. *Nat Genet*. 2020;52(9):969-983. doi:10.1038/s41588-020-0676-4

7. Rentzsch P. Witten D. Cooper GM. Shendure J. Kircher M. CADD: predicting the deleteriousness of variants throughout the human genome. *Nucleic Acids Res*. 2019;47(D1):D886-D894. doi:10.1093/nar/gky1016

8. Antikainen AA. Haukka JK. Kumar A. et al. Whole-genome sequencing identifies variants in ANK1. LRRN1. HAS1. and other genes and regulatory regions for stroke in type 1 diabetes. *Sci Rep*. 2024;14(1):13453. doi:10.1038/s41598-024-61840-7

9. Salem RM. Todd JN. Sandholm N. et al. Genome-Wide Association Study of Diabetic Kidney Disease Highlights Biology Involved in Glomerular Basement Membrane Collagen. *J Am Soc Nephrol*. 2019;30(10):2000-2016. doi:10.1681/ASN.2019030218

10. Sandholm N. Hotakainen R. Haukka JK. et al. Whole-exome sequencing identifies novel protein-altering variants associated with serum apolipoprotein and lipid concentrations. *Genome Med*. 2022;14(1):132. doi:10.1186/s13073-022-01135-6
